# Molecular Genetics of Abnormal Redox Homeostasis in Type 2 Diabetes Mellitus

**DOI:** 10.3390/ijms24054738

**Published:** 2023-03-01

**Authors:** Iuliia Azarova, Alexey Polonikov, Elena Klyosova

**Affiliations:** 1 Department of Biological Chemistry, Kursk State Medical University, 3 Karl Marx Street, 305041 Kursk, Russia; 2 Laboratory of Biochemical Genetics and Metabolomics, Research Institute for Genetic and Molecular Epidemiology, Kursk State Medical University, 18 Yamskaya Street, 305041 Kursk, Russia; 3 Laboratory of Statistical Genetics and Bioinformatics, Research Institute for Genetic and Molecular Epidemiology, Kursk State Medical University, 18 Yamskaya Street, 305041 Kursk, Russia; 4 Department of Biology, Medical Genetics and Ecology, Kursk State Medical University, 3 Karl Marx Street, 305041 Kursk, Russia

**Keywords:** type 2 diabetes, genetic susceptibility, molecular mechanisms, oxidative stress, redox homeostasis, glutathione metabolism, antioxidant enzymes, oxidative enzymes, single nucleotide polymorphism

## Abstract

Numerous studies have shown that oxidative stress resulting from an imbalance between the production of free radicals and their neutralization by antioxidant enzymes is one of the major pathological disorders underlying the development and progression of type 2 diabetes (T2D). The present review summarizes the current state of the art advances in understanding the role of abnormal redox homeostasis in the molecular mechanisms of T2D and provides comprehensive information on the characteristics and biological functions of antioxidant and oxidative enzymes, as well as discusses genetic studies conducted so far in order to investigate the contribution of polymorphisms in genes encoding redox state-regulating enzymes to the disease pathogenesis.

## 1. Introduction

Diabetes mellitus is one of the most common metabolic diseases, affecting every tenth person on the planet [1]. According to the latest edition of the IDF Diabetes Atlas, there are more than 537 million people who suffer from diabetes in the world [2]. It has been recently predicted that 643 million people will have diabetes by 2030 (11.3% of the population) [3]. If trends continue, this number will jump to a staggering 783 million (12.2%) by 2045 [2]. A great majority of diabetics in the world suffer from type 2 diabetes mellitus, or non-insulin-dependent diabetes mellitus, a serious chronic disease that develops when the body does not produce enough insulin or is unable to use it effectively [4,5,6].

Epidemiological studies of T2D conducted over the past decades indicate that T2D is a heterogeneous disease determined by genetic, epigenetic, and environmental risk factors that closely interact with each other [7,8,9]. A huge number of genetic studies have been conducted to elucidate the molecular mechanisms of T2D, including beta-cell dysfunction, insulin resistance, imbalance in redox homeostasis, and impairment of incretin signaling; from these studies, multiple disease-associated gene polymorphisms have been identified [10,11,12,13,14]. Nonetheless, many aspects of the molecular mechanisms of disease pathogenesis remain poorly characterized. In particular, numerous studies have shown that oxidative stress (the imbalance caused by excess ROS or oxidants over the cell’s ability to realize an effective antioxidant response) resulting from an imbalance between the production of free radicals and their neutralization by antioxidant enzymes is one of the major pathological disorders underlying the development and progression of type 2 diabetes.

However, the literature data on the molecular genetic mechanisms underlying abnormal redox homeostasis in type 2 diabetes have not so far been reviewed.

The present review summarizes current state-of-the art advances in understanding the role of abnormal redox homeostasis in the molecular mechanisms of T2D and provides comprehensive information on the characteristics and biological functions of antioxidant and oxidative enzymes, as well as discusses genetic studies conducted so far in order to investigate the contribution of polymorphisms in genes encoding redox state-regulating enzymes in disease pathogenesis. The following internet resources were used in preparing this review: Entrez gene [15], UniProt Knowledgebase [16], BRENDA Enzyme Database [17], GENATLAS [18], HUGO Gene Nomenclature Committee [19], GeneCards [20], BioGPS [21], Genotype-Tissue Expression (GTEx) Portal [22], eQTLGen Consortium [23], GWAS Catalog [24], and DisGeNET [25].

## 2. General Pathological Alterations in Type 2 Diabetes

It is widely agreed that chronic hyperglycemia, the primary diagnostic indicator of T2D, results from pancreatic beta-cell failure, which manifests by a gradient reduction in beta-cell mass and insulin production in response to glucose [26,27,28]. With the loss of metabolic flexibility, beta-cells begin to oxidize fatty acids instead of glucose, which results in the production of harmful byproducts (peroxides) and decreased insulin secretion [29,30]. It is noteworthy that the damage of the endocrine part of the pancreas is caused by at least three biological phenomena: apoptosis of beta-cells, their dedifferentiation, and transformation into glucagon-producing alpha cells [31,32,33,34]. A number of studies have shown a decrease in the expression of key transcription factors (PDX1, NKX6.1, and MAFA) regulating the function of beta-cells, and discussed the subsequent disorders that are accompanied by a loss of insulin-producing ability in T2D [33,35]. 

The islets of Langerhans have been found to contain bihormonal cells that produce both glucagon and insulin after the conversion of beta-cells into alpha cells, as has been demonstrated by experimental studies on transgenic mice [33,36]. A shift in the phenotype of pancreatic beta-cells may serve as a mechanism of cell mass conservation [37] since alpha cells are more resistant to metabolic stress induced by excessive nutrition. Lim and co-authors observed that changes in insulin secretion in T2D are potentially reversible due to beta-cell re-differentiation: complete recovery of the first phase of secretion was observed by the eighth week of a hypocaloric diet in 87% of T2D patients with less than four years of disease duration [38]. There is definitely a “point of no return” in the progression of the disease beyond which the lost ability to produce insulin cannot be restored due to irreversible apoptotic changes in the pancreatic islets.

The development of insulin resistance, another phenomenon accompanying T2D pathogenesis, involves supra- and post-receptor mechanisms. The former are associated with hypertrophic extracellular matrix signaling, a decrease in capillary filling, and thus deterioration of local blood flow, limiting the availability of insulin and glucose in muscles and other tissues [39,40,41]. Insulin resistance is exacerbated by an increase in the expression of collagen and other extracellular matrix proteins, including their integrin receptors, which are in direct contact with skeletal muscle capillaries [42]. It is worth noting that the formation of insulin resistance in adipose tissue is triggered by the accumulation of extracellular matrix and fibrosis [43,44]. Given that insulin-like growth factor 1 (IGF1) is a powerful stimulator of collagen expression, hyperinsulinemia can promote collagen synthesis by adipose tissue fibroblasts, leading to insulin resistance by activating IGF1 receptors or affecting IGF1 binding protein [45]. Post-receptor mechanisms underlying insulin resistance are associated with a decrease in intracellular glucose metabolism activity, including de novo fatty acid synthesis in adipose tissue [46,47,48]. These effects are associated with decreased expression of the lipogenic transcription factors ChREBP-α and ChREBP-β, which are linked to GLUT4 inactivation [49,50,51]. The importance of controlling the metabolism of acetyl-CoA is also related to the fact that this substrate is necessary for the acetylation of proteins, including histones [52,53,54]. 

Insulin resistance and Langerhans islet dysfunction appear early in the pathogenesis of T2D, as shown in observational studies of Pima Indians, whose natural course of disease progressed over time from euglycemia to impaired glucose tolerance and T2D [55,56]. There are two points of view regarding the sequence of development of insulin resistance and beta-cell dysfunction, which is often manifested as hyperinsulinemia. The first explanation states that insulin resistance is the primary cause of hyperinsulinemia. An experimental study on mice has shown that insulin resistance appears as a result of induced disorders of the insulin signaling pathway in the liver, skeletal muscle, and adipose tissue and causes hyperinsulinemia, thereby leading to the development of T2D [57,58]. In humans, mutations in genes encoding insulin signaling proteins are also manifested by an increase in circulating insulin levels in the blood and are the causes of hereditary forms of diabetes [59,60]. A decrease in the effectiveness of insulin in relation to glycemic control is associated with the activation of the FOXO1 transcription factor in the liver [61,62] and impaired translocation of the glucose transporter GLUT4 into the membranes of skeletal myocytes [63,64]. Consequently, FOXO1 increases the expression of key enzymes of gluconeogenesis, causing the liver to produce more glucose. A decrease in GLUT4 in the membrane of skeletal myocytes reduces the uptake of glucose from the bloodstream into the cells. In the liver, insulin normally causes phosphorylation and suppression of FOXO1 by protein kinase B (Akt), which keeps FOXO1 in the cytoplasm, where this transcription factor is inactive [65,66,67]. However, FOXO1 expression was found to be increased in the liver of obese mice, and this transcription factor loses its sensitivity to insulin regulation [68,69,70]. Impact of overnutrition on the FOXO1 dysregulation is currently being extensively researched [71,72] to better understand the primary mechanism of uncontrolled gluconeogenesis in obesity [73,74,75]. It is thought that hyperglycemia caused by uncontrolled FOXO1 activation, combined with chronic hyperinsulinemia, can remove insulin’s inhibitory effect on lipolysis in adipose tissue [69]. Activated lipolysis in abdominal adipocytes in turn increases the flow to the liver of its products—free fatty acids, which are allosteric activators of pyruvate carboxylase, a key enzyme of gluconeogenesis, and glycerol, a substrate of the same pathway [76]. Thus, a high-calorie diet induces the production of glucose by the liver under the influence of FOXO1, which is additionally stimulated by the activation of lipolysis in adipose tissue. In this context, functional beta-cell deficiency results in an inability to secrete enough insulin to compensate for the effects of FOXO1, resulting in diabetes [77,78].

An alternative hypothesis of T2D pathogenesis implies that hyperinsulinemia is the primary cause of insulin resistance. In obese people without diabetes, fasting hyperinsulinemia is found without detectable hyperglycemia, which is theoretically necessary to stimulate additional insulin secretion by beta-cells. This apparent disparity between blood glucose and insulin levels led to the hypothesis that hyperinsulinemia is the initial, primary effect of overeating and obesity [79,80] and is caused by stimulation of insulin secretion [81,82] and suppression of its degradation [83]. Insulin resistance in the liver is caused by primary hyperinsulinemia, which is associated with the suppression of signal transmission from the insulin receptor to Akt. Activation of the Akt protein appears to be sufficient to activate the mTORC1 kinase complex and the SREBP-1c transcription factor, both of which promote de novo fatty acid synthesis [84]. Furthermore, hyperinsulinemia has been found to cause activation of inflammatory signaling pathways in humans [85,86,87] and rats [88], which can impair insulin sensitivity in the target tissues [89]. A similar increase in glucose tolerance was observed in the knockout mice with impaired insulin secretion [90]. Thus, both beta-cell dysfunction and insulin resistance are disorders that can occur at the same time and are not mutually exclusive.

## 3. Environmental Risk Factors of Type 2 Diabetes

Type 2 diabetes is a multifactorial disease that develops as a result of interactions between environmental and genetic factors [91,92]. It has been estimated that the majority of the burden of type 2 diabetes is related to environmental exposures and modifiable risk factors, such as lifestyle [93,94]. High-calorie diets, high consumption of fatty foods and refined carbohydrates, hypodynamia, and smoking are modifiable risk factors for T2D, whereas gender, age, and family history of diabetes are non-modifiable risk factors. 

The leading causes underlying the epidemics of T2D and obesity include urbanization, sedentary lifestyles, and unhealthy diets [26]. Undoubtedly, nutritional status is one of the most important environmental factors influencing the risk of T2D [26]. Food quality is more significant than overall food intake, as evaluated by naturally occurring polyunsaturated fatty acid content, the lack of trans-unsaturated fatty acids, and meals with a high glycemic index. An increase in dietary fat intake raises insulin levels in contrast to normal glycemia [95]. Carter P. et al. [96] observed that daily eating of fresh fruits and vegetables reduces the risk of disease by 14%. According to a study by Cooper A.J. and co-authors [97], a diet with a greater quantity of vegetables and a greater variety of both fruits and vegetables in diet is associated with a reduced risk of type 2 diabetes. It has been found that there is an inverse relationship between consumption of plant food and the risk of disease [98,99]. It is estimated that consuming even four servings of fruit each day can lower the incidence of T2D by 7% [99]. Increased consumption of fresh, plant-based foods and whole-meal breads, as well as decreased consumption of sugary drinks, high-calorie, fried foods, and white bread, are among the nutritional recommendations made by the WHO for the prevention of T2D [100]. Numerous studies have demonstrated that the Mediterranean diet lowers the incidence of T2D [101,102]. The fast-food boom is one of the factors that’s caused the pandemic spike in T2D incidence in Asia (China and South Korea) over the past two decades [103]. Clinical studies, such as the Diabetes Prevention Program in the USA, the Finnish Diabetes Prevention Studies in Finland, the Da Qing IGT, and the Diabetes Study in China, have demonstrated the benefits of changing lifestyle, particularly through a healthy diet and increased physical activity.

Physical inactivity is associated with poor glucose tolerance, hyperinsulinemia, and insulin resistance, which dramatically raises the risk of T2D. The prevalence of type 2 diabetes was found to be two times higher among those who have a sedentary lifestyle than those who engage in strenuous physical activity [104,105,106]. Prospective studies have demonstrated a positive relationship between aerobic and power loads and the risk of T2D [107,108]. Ekelund et al. [109] calculated that the reduction in T2D risk caused by moderate and vigorous physical activity depends only on the length of training and does not depend on the amount of time spent in an inactive state. Rockette-Wagner [110] showed that long-term sedentary work can neutralize the positive effect of physical activity in terms of the T2D risk. Numerous tissues, including skeletal and cardiac muscle, the liver, and brain cells, have been found to be involved in the regulation of redox homeostasis by physical exercise [111]. A body of evidence shows that disruption in exercise-induced redox homeostasis serves as an upstream signal for transcription factor activation, followed by gene expression stimulation, increased antioxidant defense, and decreased age-related oxidative stress [112,113]. 

Tobacco smoking is also an environmental risk factor for T2D [114]. The number of cigarettes smoked per day was found to be dose-dependently correlated with the risk of T2D, which was found to be 45% higher in smokers than nonsmokers [115]. There is evidence that passive smoking also increases the incidence of T2D [116]. Smoking is an independent factor that worsens tissue insulin sensitivity. Tobacco use has been shown in studies to be toxic to the pancreas and to reduce insulin release by beta-cells [117]. 

Alcohol drinking patterns have been found to be associated with the risk of type 2 diabetes [118]. Okamura T. and colleagues discovered that heavy alcohol consumers (>280 g/week) with fatty liver disease (FLD) had a higher risk of developing type 2 diabetes than the other groups. Moderate alcohol consumers (140–280 g/week) without FLD had a significantly higher risk for type 2 diabetes, compared with minimal (<40 g/week) and light (40–140 g/week) alcohol consumers without FLD. In contrast, there was no apparent difference in the risk for incident type 2 diabetes between non-drinkers and minimal, light, or moderate alcohol consumers with FLD. Furthermore, no significant difference in the risk for T2D was found between moderate and heavy alcohol drinkers without FLD and non-drinkers or minimal, light, or moderate alcohol consumers with FLD [119].

The prevalence of type 2 diabetes, like many other multifactorial diseases, is linked to cumulative exposure to lipophilic and hydrophilic environmental pollutants such as POPs (persistent organic pollutants), exudates produced by common plastics, air pollutants, and some medicines [120,121]. Prospective studies investigating the dose-response relationship between environmental pollutants and T2D prevalence are needed to substantiate the causal role of chemical factors in disease susceptibility.

## 4. Genetic Factors of Type 2 Diabetes

Studies in multiethnic populations with varying T2D rates suggest that some ethnic groups may have a genetic predisposition to developing insulin resistance and diabetes when exposed to adverse conditions [122]. It is thought that between 35% and 70% of type 2 diabetes cases are genetically predisposed [123]. The genetic contribution to diabetes is demonstrated by familial aggregation [124] and a high concordance rate for T2D in monozygotic twins. In particular, the relative risk of T2D is 35–39% if one parent suffers from the disease; the risk is 60–70% if both parents have T2D; the risk of T2D in monozygotic twins is 58–65%; and the risk in heterozygotic twins is 16–30% [125,126].

Genome-wide association studies (GWAS), a hypothesis-free method created to test hundreds of thousands of genetic variants across many genomes to find those statistically associated with a specific trait or disease, have made significant progress in the last two decades in identifying the genes that cause diabetes susceptibility [127]. Numerous GWASs have identified multiple genetic loci that are linked to T2D and disease-related phenotypes [9,10,128]. According to the GWAS Catalog (https://www.ebi.ac.uk/gwas/, accessed on 25 February 2023), there have been 222 genome-wide studies on type 2 diabetes and 5348 SNP-disease associations have been detected. Furthermore, large meta-analyses of GWAS were performed [10,129] and identified 2284 SNPs associated with T2D, but only 183 and 38 loci were successfully replicated as T2D susceptibility markers once and twice, respectively [130]. Thus, a large portion of the originally discovered SNP-disease associations were poorly replicated by independent studies [131,132,133]. 

*TCF7L2, HHEX, KCNJ11, CENTD2, ARAP1, FTO, HNF1B, PPARG, IGF2BP2, CDKAL1, VEGFA, SLC30A8, CDKN2A, CDKN2B, KCNQ1, UBE2E2, ANK1, ABCC8, IRS1,* and *GLP2R* are the genes whose polymorphic variants showed strong associations with susceptibility to T2D and related phenotypes [134,135,136]. The variants most strongly associated with disease risk affect gene expression in the islets of Langerhans as well as insulin-sensitive tissues and organs such as adipose, muscle, and liver. Transcriptome studies [137,138] showed that some differentially expressed genes (DEGs) are associated with a decrease in insulin secretion (*ZMIZ1, MTNR1B, ADCY5, GIPR, C2CDC4A, CDKAL1, GCK, TCF7L2, GLIS3, THADA,* and *IGF2BP2*), while other DEGs are associated with insulin resistance (*PPARG, KLF14,* and *IRS1*) and a decrease in the incretin response (*TCF7L2, KCNQ1, GIPR, THADA, WFS1,* and MTNR1B). However, a larger portion of the identified genes are not associated with either production and/or action of insulin or glucose metabolism, and their role in disease pathogenesis remains unknown. These genes produce proteins that are involved in signaling pathways (*ACHE, ADCY5, ARAP1, ARL15, ATP8A1, BECN1, BRAF, CAMKK2, CMIP, DAAM1, DCDC2C, GATAD2A, GPSM1, GRK5, IGF2BP2, MAEA, MCC, PLEKHA1, RGS7, SHB, SUGP1, SYK*, and *TMEM132D*), antigens and receptors (*GABRA4, GIPR, GLP2R, IL17REL, IL23R, LRP12, MTNR1B, NOTCH2, NRXN3, PPARG, PTPRD, PVRL2, RASGRP1, SSR1, TGFBR3*, and *THADA*), transport proteins (*ATP8A1, EXOC6, KCNJ11, KCNQ1, SLC16A11, SLC16A13, SLC9B2, SLCO4C1, VPS26A, VPS33B, WFS1,* and *YKT6*), proteins regulating cell cycle, mitosis and apoptosis (*BCL2, CENPW, FAM58A, FAM60A, MPHOSPH9, NDUFAF6, RCCD1, RHOU,* and *ZZEF1*) and other proteins (*ANK1, FSCN3, INTS8, MACF1, SGCD, SGCG, LAMA1, LIMS2,* and *ST6GAL1*). SNP rs7903146 at the *TCF7L2* (transcription factor 7 like 2) gene is a genetic marker, the most strongly associated with T2D susceptibility, and it has been successfully replicated in twenty-two studies in various populations of the world [139,140,141,142,143,144]. Polymorphisms of *TCF7L2, IGF2BP2, CDKAL1, KCNQ1*, and *PPARG* genes showed a strong contribution to type 2 diabetes. 

The rs7903146 polymorphism of *TCF7L2*, one of the most strongly T2D-associated loci, has been convincingly linked to diminished incretin responsiveness, elevated hepatic glucose production, and impaired insulin secretion [145,146]. It has been experimentally shown that turning off the *TCF7L2* gene in mouse beta-cells impairs glucose tolerance and reduces beta-cell mass [147]. Functional studies have confirmed the essential role of the gene in the control of glucose homeostasis through the regulation of beta-cell mass. TCF7L2 is a dual-acting transcription factor that participates in the Wnt signaling pathway, operating as an activator when CTNNB1 beta-catenin is present and as a repressor when it is not [148]. TCF7L2 also inhibits adipogenesis via NLK (Nemo-like kinase) in adipose tissue and inhibits gluconeogenesis in the liver [149]. 

The rs11927381 variant of the *IGF2BP2* gene is a marker that has been shown to be strongly associated with T2D susceptibility [150,151]. *IGF2BP2* encodes a protein that binds insulin-like growth factor 2 (IGF2) mRNA and was found to be associated with both beta-cell dysfunction and decreased sensitivity of peripheral tissues to its effects [152]. Associations of six polymorphisms of the *IGF2BP2* gene such as rs4402960, rs1470579, rs7640539, rs71320321, rs1374910, and rs6769511 with type 2 diabetes have been discovered and subsequently replicated by independent studies [134]. Dai and co-authors have revealed that an increase in *IGF2BP2* expression can cause a deficit of mitochondrial uncoupler protein 1 (UCP1) and hyperproduction of IGF2 because the IGF2BP2 protein facilitates the translation of insulin-like growth factor 2 (IGF2) mRNA and inhibits the synthesis of UCP1 [152]. Mice knocked out of the *IGF2BP2* gene showed that a decrease in UCP1 synthesis worsens tissue sensitivity to insulin and contributes to the development of alimentary obesity [152]. Additionally, a study [153] discovered an inverse correlation between the level of *IGF2BP2* gene expression in the blood and serum insulin concentration in T2D patients. Experiments with transgenic mice have shown that an increased level of IGF2 leads to insulin resistance, hyperglycemia, and diabetes in 30% of the animals [154]. An increase in IGF2 levels was found to cause disruption of pancreatic islets, whereas a decrease in IGF2 expression in human liver, muscles, and beta-cells reduced the risk of T2D [155].

*CDKAL1* (cyclin dependent kinase 5 regulatory subunit associated protein 1 like 1) is another gene whose polymorphisms (rs4712524, rs6931514) are linked to beta-cell dysfunction [156]. This enzyme regulates cyclin-dependent kinase 5, an activator of insulin production in pancreatic beta-cells [157,158]. As part of the formation of ms2t6A37, the enzyme transfers the thiomethyl group from the S-adenosylmethionine molecule to the N6-threonylcarbamoyladenosine at position 37 of the cytosolic tRNALys (t6A37), which is required for the proper reading of lysine codons during translation of the proinsulin mRNA [159]. The accurate recognition of the AAA and AAG lysine codons during preproinsulin mRNA translation is made possible by CDKAL1-catalyzed tRNA modification [160]. The lysine residue is located at the cleavage site of proinsulin into the A-chain and C-peptide; therefore, translational errors can disrupt the formation and secretion of insulin in pancreatic beta-cells. Loss-of-function SNPs in the introns of the *CDKAL1* gene were found to be associated with defects in insulin secretion but not with obesity or insulin resistance [161,162].

The first gene described in candidate gene studies and subsequently replicated in GWAS was the *PPARG* (peroxisome proliferator activated receptor gamma) gene [163]. The gene encodes a nuclear transcription factor involved in the regulation of hundreds of genes for carbohydrate and lipid metabolism. PPARG controls the expression of lipoprotein lipase and CD36 fatty acid transporter [128]. Adipocytes with activated expression of PPARG secrete leptin and adiponectin in a balanced manner, which mediates the effects of insulin on peripheral organs. It has been shown that polymorphisms in the *PPARG* gene are involved in the development of both insulin resistance and impaired insulin secretion by pancreatic beta-cells [164]. In particular, the rs11709077 polymorphism of *PPARG* is associated with T2D, obesity, and cardiovascular diseases [165].

The *KCNQ1* gene encodes the potassium channel protein of beta-cells and is directly involved in the process of glucose-stimulated insulin secretion [166]. Polymorphism rs2237896 of the *KCNQ1* gene was found to be associated with dysfunction of beta-cells and decreased incretin response [167].

Thus, genome-wide association studies have discovered hundreds of SNPs linked to T2D. The discovered disease-associated alleles influence functional properties of pancreatic beta-cells and sensitivity of insulin-dependent tissues. However, the molecular processes by which many susceptibility genes are implicated in the pathogenesis of T2D are yet recognized and are the subject of ongoing research [168,169]. The polygenic nature of this complex disease, epistatic interactions between genes, effects of environmental factors, variable penetrance (between 10 and 40%), and high frequency of disease-related alleles with weak or moderate effects on disease phenotype make it difficult to understand the primary molecular mechanisms underlying T2D [170].

## 5. Glutathione Metabolism and Oxidative Enzymes as the Core of Redox Homeostasis

Cellular redox homeostasis is a crucial dynamic process that maintains the balance of reducing and oxidizing reactions within cells and governs a wide range of biological responses and events. Reactive oxygen species are required for life and play a role in practically all biological activities. Excess ROS are neutralized by antioxidant enzymes that catalyze the conversion of reactive oxygen species and their byproducts into stable harmless compounds. Redox homeostasis controls a wide range of biological responses and events by ensuring the balance between oxidation and reduction reactions within the cell [171]. The roles of reactive oxygen species in redox homeostasis and cellular signaling were reviewed in detail [172,173]. Increased levels of reactive oxygen species (ROS) damage macromolecules, impair redox signaling, and were found to be associated with beta-cell dysfunction and insulin resistance [174,175,176]. To reduce the harm of free radicals, cells use both enzymatic and non-enzymatic antioxidant mechanisms. Superoxide dismutase (SOD), catalase (CAT), glutathione peroxidase (GPX), and glutathione S-transferases (GST) comprise the enzymatic part of antioxidant defense, while vitamin A, ascorbic acid (vitamin C), and alpha-tocopherol (vitamin E) represent non-enzymatic antioxidants [177,178]. Supplementary Table S1 summarizes the biological functions and tissue-specific gene expression of antioxidant enzymes that fight against oxidative stress by multiple mechanisms, including the conversion of superoxide anion into hydrogen peroxide (SOD), breakdown of hydrogen peroxide (CAT, GPX), reduction of disulfide bonds into dithiol-containing ones (thioredoxin reductase), biosynthesis of glutathione (glutamate-cysteine ligase, glutathione synthase, glutathione reductase), and conjugation of xenobiotic substrates with glutathione (GST).

Glutathione-dependent enzymes constitute a crucial antioxidant system and control thiol-disulfide equilibrium. Thiol-dependent peroxidases require electrons to deactivate free radicals with the help of reduced glutathione (GSH). Glutathione is the master antioxidant, capable of protecting cellular components from reactive oxygen species, free radicals, peroxides, lipid peroxides, and heavy metals; it also participates in DNA repair, immunological response, and controls cell death [179]. Furthermore, glutathione determines the activity of several enzymes, acts as a mediator of signaling pathways, regulates the cellular life cycle and division, and promotes protein folding as well as acts as a cysteine depot and a source of vitamins C and E [174,180]. Glutathione is converted by free radical scavenging into the oxidized form known as the GSSG dimer, which is involved in glutathionylation, post-translational protein modification, and epigenetic control of gene expression [181,182,183].

The main metabolic pathway for glutathione biosynthesis is the gamma-glutamyl cycle, which begins with the ATP-dependent formation of gamma-glutamylcysteine from glutamate and cysteine (https://www.kegg.jp/pathway/map00480, accessed on 22 January 2022). The reaction is catalyzed by the enzyme glutamate cysteine ligase (GCL), consisting of modifying (GCLM) and catalytic (GCLC) subunits [184]. In the second step, glutathione synthetase (GSS) is used to add glycine to the cysteine residue of the γ-Glu-Cys dipeptide. GSH is then released from the cell. Since the concentration of glutathione in blood plasma is 1000 times lower than that in a cell’s cytosol, the current gradient acts as a driving force for glutathione transport. Once GSH has left the cell, gamma-glutamyl transferase (GGT) replaces the glutathione-Cys-Gly moiety with an alternative amino acid (phenylalanine, leucine, or lysine). Following the hydrolysis of the dipeptide Cys-Gly by dipeptidase into cysteine and glycine, these amino acids are delivered into the cell via specialized membrane transporters [185]. Gamma-glutamyl-amino acid, a product of the GGT reaction, is also transported into the cell, where it is converted into an amino acid and oxoproline by gamma-glutamyl cyclotransferase (GGCT). With the use of a mole of ATP, oxoproline is transformed into glutamic acid by the enzyme oxoprolinase. Therefore, the cell contains all three of the amino acids required to replenish GSH. In order to assure the formation of GSH and to supply some positively charged (lysine) and non-polar (phenylalanine, leucine) amino acids into the cell, one cycle turn requires the consumption of three ATP molecules [186].

The ChaC-family of proteins [187], which catalyzes a process similar to that of the GGCT enzyme but uses glutathione itself as a substrate, is one of the new enzymes implicated in glutathione catabolism that have been discovered in recent years. This fact has led to a considerable revision of theories regarding GSH degradation and the roles of GGT and GGCT in glutathione metabolism. GGT catalyzes the hydrolysis of GSH into glutamate and cysteinylglycine (1): transpeptidation of amino acids (2):

GSH + H_2_O → Glu + Cys-Gly (1)

GSH + AA → γ-Glu-AA + Cys-Gly(2)

Several experimental studies showed that there is no connection between GGT activity and the transport of amino acids into the cell. Glutathione is transported by membrane glutathione transporters, including the MRP protein (multidrug resistance protein) and the ABC (ATP binding cassette) family transporter [180]. As it turns out, the main function of gamma-glutamyl transferase is the reutilization of GSSG or GSH conjugates, which are released from the cell into plasma, where they undergo conversion into glutamate and Cys-Gly or dimer (Cys-Gly)_2_ by GGT. The resulting peptides can be transported into the cell by appropriate proteins or can be hydrolyzed into cysteine and glycine, which are taken up by the cells using specific transporters [188]. Thus, glutathione enters the cell “in parts”—in the form of its individual amino acids or dipeptides, which are subsequently used for de novo synthesis of GSH.

Gamma-glutamyl cyclotransferase was first described by Meister as one of the most important enzymes of the γ-glutamyl cycle [185]. GGCT also regulates de novo glutathione synthesis through its activity against glutamylcysteine, which is also a substrate of glutathione synthase [189]. Glutamate cysteine ligase normally generates a product that is targeted toward the production of GSH, in contrast to GGCT, which has a lesser affinity for γ–glutamylcysteine [190]. Finally, under conditions of Cys deficiency, GCL can catalyze the condensation of Glu with a non-Cys amino acid, forming γ-glutamyl-AA, a potential GGCT substrate converted to the amino acid and 5-oxoproline. 

The second source of GSH formation is the enzyme glutathione reductase, which catalyzes the reduction of the GSSG dimer into a functionally active monomer according to the equation:

GSSG + NADPH H^+^ → 2GSH + NADP^+^

It is well known that viability of the cell depends on maintaining the optimal ratio between reduced to oxidized glutathione (GSH:GSSG) [191,192,193]. A decrease in the activity of any enzyme involved in the metabolism of glutathione may shift the balance between reduced and oxidized glutathione and encourage the buildup of free radicals in tissues. In addition, an increase in the activity of oxidative enzymes generating ROS may cause glutathione depletion. 

The main source of ROS in the cell is the mitochondrial electron transport chain, along with NADPH oxidase and nitric oxide synthase [194,195]. ROS are also produced during the folding of proteins, a process of disulfide bond formation in the endoplasmic reticulum, as well as a result of the activity of cytochromes P450 in the metabolism of xenobiotics [196]. Phagocytic cells such as neutrophils and monocytes utilize free radicals to destroy internalized microorganisms, whereas in non-phagocytic cells, ROS act as signaling molecules regulating a variety of biological processes such as cell division, regeneration, cell differentiation, their apoptosis, and cytoskeleton organization [197]. The combination of increased free radical compound production and inefficient antioxidant defense enzyme function creates the foundation for the development of oxidative stress, a pathological condition thought to be responsible for the development of complications in type 2 diabetes [198,199,200].

The biological functions and tissue-specific gene expression of oxidant enzymes producing superoxide anion (NADPH oxidases), hydrogen peroxide (dual oxidases, monoamine oxidases, lysyl oxidase), and hypochlorous acid (myeloperoxidase) are also summarized in Supplemental Table S1.

As mentioned above, NADPH oxidase is the main oxidant enzyme responsible for the generation of superoxide anion radicals:

NADPH·H^+^ + 2O_2_ = 2O_2_·^−^ + NADP^+^ + H^+^

As a result of the dismutation reaction, O_2_ turns into H_2_O_2_.

The NADPH oxidase consists of transmembrane catalytic core proteins such as CYBA and CYBB, four cytosolic subunits—NCF1, NCF2, and NCF4 as well as small GTP-ases RAC1/RAC2 [201,202]. Noteworthy is the fact that only CYBB, out of all the NADPH oxidase subunits, has binding domains for the cofactors NADPH, FAD, and two heme molecules. This means that CYBB is directly involved in the formation of superoxide anion.

Nitric oxide synthases (NOS1, NOS2, and NOS3) and myeloperoxidase (MPO), which have been implicated in the development of type 2 diabetes, are also important ROS-generating enzyme families [203,204,205,206]. The three isoforms of the nitric oxide synthase family members catalyze the generation of NO from L-arginine: neuronal (nNOS/NOS1), inducible (iNOS/NOS2), and endothelial (eNOS/NOS3). Impairment in NO generation is known to be associated with endothelial dysfunction, insulin resistance, and diabetes. Several studies have revealed that single nucleotide polymorphisms (SNP) such as rs2297518 of *NOS2* and rs1799983 of *NOS3* are linked to the onset of T2D and/or its microvascular complications [206,207,208,209]. 

In addition, decreased antioxidant defense is a common finding in type 2 diabetes, which may be partially attributed to the spontaneous glycosylation of key antioxidant enzymes such as catalase, superoxide dismutase, glutathione-S-transferases, glutathione peroxidase, glutathione reductase, and others, as well as depletion of the intracellular pool of NADPH·H+ [85,210]. It is observed that enhanced conversion of sorbitol to fructose under the action of NAD+-dependent sorbitol dehydrogenase reduces the ratio of NAD+/NADH·H+ in the cell, leading to inhibition of glycolytic enzyme glyceraldehyde-3-phosphate dehydrogenase (GAPDH), accumulation of glyceraldehyde-3-phosphate and its conversion to diacylglycerol, a known allosteric activator of protein kinase C [211]. GAPDH, along with advanced glycation end products (AGEs), serves as a potent inducer of NADPH oxidase [212].

Reactive oxygen and nitrogen species and carbonyl compounds are respectively generated under oxidative, nitrosative, and carbonyl stress conditions, which have been found to play a role in the development and progression of type 2 diabetes [85]. Dysregulation of redox homeostasis in T2D was demonstrated at both genetic and biochemical levels [204].

## 6. Endogenous Deficiency of Glutathione in Type 2 Diabetes

Numerous environmental factors have been identified as risk factors for type 2 diabetes, and many of these same factors are responsible for the depletion of the endogenous glutathione pool, highlighting the importance of glutathione and redox homeostasis in the development of type 2 diabetes. Glutathione is the most prevalent non-protein thiol in cells that functions as the primary reducing agent and provides antioxidant defense against oxidative cell damage [213]. The millimolar level of reduced glutathione is maintained within cells, illustrating its essential biological functions that extend beyond antioxidant defense [214]. Glutathione is required for the detoxification of xenobiotics and endogenous toxic substances, the maintenance of mitochondrial redox balance, direct antiviral defense, immune response, vitamin C and E regeneration, control of cell proliferation, apoptosis, and protein folding [213,214,215]. Given the wide range of important biological functions of reduced glutathione, it is predicted that a GSH deficit plays a substantial role in the onset of a number of disorders [216,217]. It is observed that individuals with type 2 diabetes exhibit impaired redox homeostasis and oxidative stress attributed to, on the one hand, a deficiency in the production of endogenous antioxidants and, on the other hand, an excess of free radicals [218]. Decreased antioxidant defense and oxidative stress are linked to key pathophysiological abnormalities underlying type 2 diabetes mellitus, such as pancreatic beta-cell dysfunction and insulin resistance [219,220,221]. Lutchmansingh and colleagues discovered that T2D patients have lower levels of the reduced form of glutathione and a slower rate of its synthesis in erythrocytes than healthy individuals [193]. Furthermore, several studies [193,222,223] have revealed that depletion of the endogenous glutathione pool correlated to increased blood glucose levels and contributed to disease complications. An experimental study by Stumvoll and colleagues observed that glutathione appears to have the ability to prevent beta-cell insufficiency and reduce glucose tolerance in rats fed a long-term high-glucose diet [223].

Reduced glutathione synthesis in T2D is thought to be caused by a lack of the amino acid precursors of GSH, cysteine, and glycine [224], whereas replenishing glutathione deficiency in diabetic patients leads to improvements in metabolic disorders and disease symptoms. In particular, intravenous glutathione infusions were found to improve tissue insulin resistance [225]. The induction of GSH biosynthesis by activation of the PI3K/Akt/p70S6K signaling pathway was found to significantly improve the therapeutic effect of insulin therapy in patients with T2D [226]. A study by Murakami and colleagues found a decreased activity of key enzymes responsible for glutathione biosynthesis, such as glutamate-cysteine ligase and glutathione reductase, along with decreased levels of reduced glutathione and increased levels of oxidized glutathione in the erythrocytes of type 2 diabetics [227]. In addition, some studies showed decreased rates in the membrane transport of glutathione disulfide in diabetics [228,229]. Inhibiting glutamate-cysteine ligase activity resulted in increased hydrogen peroxide accumulation and decreased insulin transcript levels in pancreatic beta-cells [230], suggesting an important role for glutathione in the regulation of the transcriptional activity of insulin.

The ability of cells in different tissues and organs to synthesize glutathione varies, and many tissues with a limited capacity to synthesize GSH require amino acid precursors from extracellular glutathione synthesized in the liver to enter the cell. The liver, skeletal muscles, and kidneys are examples of tissues and organs with significant metabolic activity and glutathione synthesis, whereas the pancreas, despite increased activity of biosynthetic processes, has a considerably lower potential to produce glutathione [231,232]. In the pancreas, expression of GGT1, GGT6, and ANPEP enzymes, which catabolize extracellular glutathione, is far more abundant than glutathione biosynthesis enzymes such as GCLC, GCLM, GSS, GSR, and GGCT (Supplementary Table S1), meaning that the pancreatic tissues need glutathione transported from the liver [233]. It is critically important that gamma-glutamyl transferase and aminopeptidase N (ANPEP), membrane-associated enzymes that catalyze the breakdown of extracellular GSH into its individual amino acids and promote its uptake into the cell, are responsible for controlling this process [234,235]. This means the pancreas is dependent on the activity of membrane-associated enzymes that catabolize GSH and provide amino acid precursors for the synthesis of glutathione in the cell. Notably, patients with T2D and prediabetes had significantly higher plasma levels of gamma-glutamyl transferase [236,237] and mRNA levels of *ANPEP* in pancreatic islets than non-diabetics [238,239]. We recently hypothesized that increased gamma-glutamyltransferase and aminopeptidase N levels reflect a cell’s adaptive response to intracellular glutathione deficiency found in T2D, and that an increase in these enzyme levels is required to promote extracellular peptide degradation, providing amino acid precursors for de novo GSH biosynthesis [240]. We also suggested that glutathione deficiency may contribute to the disruption of protein folding in the endoplasmic reticulum [240], since glutathione is required for promoting the formation of disulfide bonds in the tertiary structure of proteins and correcting non-naive formed S-S-bonds [241,242]. Impaired proinsulin folding, observed in type 2 diabetes by several studies [242,243,244,245,246], may be attributed to reduced glutathione level in pancreatic beta-cells. The accumulation of unfolded or misfolded proinsulin molecules may trigger endoplasmic reticulum stress and the unfolded protein response—abnormalities culminating in beta-cell apoptosis in type 2 diabetes [245,247,248]. Deeper genetic and biochemical studies of the redox homeostasis system using cell lines and animal models will shed light on the mechanisms by which glutathione deficiency triggers the development and progression of type 2 diabetes mellitus.

## 7. Genes Encoding Antioxidant Defense Enzymes and the Risk of Type 2 Diabetes

Variation in genes encoding antioxidant and oxidative enzymes may affect their amount or function, shifting redox homeostasis towards oxidative stress and increasing the risk of type 2 diabetes mellitus. Several genetic association studies have shown that single nucleotide polymorphisms (SNP) in genes for redox state-regulating enzymes are linked with T2D susceptibility. Table 1 summarizes the results of genetic studies that identified associations between these genes and the risk of type 2 diabetes. In particular, the following polymorphisms at genes encoding antioxidant defense enzymes were found to be associated with the risk of type 2 diabetes: del/del of *GSTM1* [249,250,251,252,253,254], del/+ *GSTT1* [249,250,251], rs1695 [251,252,255] and rs1138272 [249] of *GSTP1*, rs12524494 of *GCLC*, rs3827715 and rs41303970 of *GCLM* [256], rs13041792 of *GSS* [240], rs2551715 of *GSR* [257], rs11546155 and rs6119534 of *GGT7* [240], rs4270 of *GGCT* [258], rs1050450 of *GPX1* [259], rs4902346 of *GPX2* [260], rs769217 of *CAT* [261], rs2234694 of *SOD1* [261], rs4880 of *SOD2* [212], and rs2536512 of *SOD3* [262]. However, much of the genetic research has focused on polymorphisms in the glutathione S-transferases M1, T1, and P1 (enzymes that used reduced glutathione for xenobiotic conjugation reactions); loss-of-function variants of these genes have been linked to an increased risk of type 2 diabetes. A limited number of studies looked into the link between T2D and genetic variations in hydrogen peroxide-metabolizing enzymes such as superoxide dismutases types 1, 2, and 3, glutathione peroxidases types 1 and 2, and catalase. The genes encoding enzymes directly involved in the metabolism of glutathione have received less attention in diabetes research. The great majority of the investigations were undertaken by our research team to explore for a link between polymorphisms at genes for glutathione metabolism and T2D susceptibility. Below, we describe the results of genetic studies that observed the associations between polymorphisms of genes for antioxidant enzymes and type 2 diabetes risk.

*Glutamate cysteine ligase* (GCL), also known as gamma-glutamylcysteine synthetase, is the first rate-limiting enzyme in glutathione biosynthesis from L-cysteine and L-glutamate. The enzyme is composed of two subunits: a heavy catalytic subunit (GCLC) and a light regulatory subunit (GCLM). *GCLC* gene is highly expressed in the liver whereas *GCLM* has the highest expression in pancreatic islets (Supplementary Table S1). Our recent study showed that polymorphisms such as rs12524494 in the *GCLC* gene and rs41303970 in the 5’-flanking region of the *GCLM* gene confer protection against T2D in nonsmokers [256]. The minor allele rs3827715-C *GCLM* also showed an association with a decreased risk of T2D. In addition, rs2301022 *GCLM* was associated with decreased levels of ROS, while SNPs rs7517826 and rs41303970 of the gene were associated with increased levels of total GSH in the plasma of T2D patients. These findings are supported by the eQTL analysis, which showed that the rs41303970-A allele was associated with a decreased level of *GCLM* gene expression in various tissues, including the pancreas. It is known that the rs41303970-A allele *GCLM* suppresses oxidant-induced gene expression and is associated with lower plasma levels of reduced glutathione and a risk of myocardial infarction [263]. A study by Ma et al. showed that the rs41303970-A allele *GCLM* was associated with liver damage in patients with viral hepatitis B [264]. Chromatin immunoprecipitation and high-throughput sequencing data from the Roadmap Epigenomics Consortium project show that rs41303970 was located in the region of epigenetic regulation of *GCLM* gene expression in numerous tissues, including pancreatic islets. In particular, SNP rs41303970 is located at the transcription start site and was associated with an epigenetic modification in the region of the H3K4me3 promoter [265]. Moreover, rs41303970 is located in the region of the histone modifier H3K27ac [266]. In addition, rs41303970 falls into the region of the H3K9ac epigenetic modification (acetylation at the 9th lysine residue of the H3 histone protein) [267]. Thus, the observed protective effect of the rs41303970-A allele against the risk of T2D is apparently attributed to its positive effect on the *GCLM* gene expression, thereby increasing glutathione production in the cell. 

*Glutathione synthetase* (GSS) is the enzyme responsible for the second step in glutathione biosynthesis. *GSS* is highly expressed in the liver and pancreatic islets (Supplementary Table S1). Although the *GSS* gene is polymorphic, no studies investigating the relationship between its polymorphisms and diseases have been conducted so far. The rs13041792 polymorphism of the *GSS* gene is associated with the level of protein C in blood plasma [268]. Our recent study showed that carriers of the rs13041792-G/A genotype possess an increased risk of type 2 diabetes [240]. The GTEx portal’s data show that the rs13041792 polymorphism has no effect on expression of the *GSS* gene in the pancreas and other tissues. Instead, the rs13041792-A allele is associated with increased pancreatic expression of other genes such as *EDEM2, PROCR,* and *MYH7B*. We also observed that *GSS* gene polymorphisms correlated with redox homeostasis parameters, such as the levels of glutathione and ROS, as well as with fasting blood glucose [240]. The relationship between the variation at the *GSS* gene and susceptibility to T2D is also supported by the finding that the rs6088660-C and rs13041792-A alleles are associated with increased levels of fasting blood glucose [240]. Taken together, these findings may indicate that the *GSS* gene polymorphisms are functionally significant variants with the potential to affect glutathione metabolism. Functional effects of the SNPs might be attributed to linkage disequilibrium with variants of neighboring genes such as *EDEM2, MYH7B,* and *PROCR*. rs13041792 is located in the 5’-untranslated region 1.4 kb from the transcription start of the *GSS* gene and is in strong linkage disequilibrium (D’ ≥ 0.91) with SNPs in nearby or adjacent genes such as *MYH7B* (rs6120772, rs7268266, rs6120788, rs3746436, and rs3746435), *MIR499A* (rs3746444), and *TRPC4AP* (rs752075). A SNP rs3746444 of *MIR499A* is one of the polymorphisms strongly linked to the rs13041792 variant of the *GSS* gene.

*Gamma-glutamyl transpeptidase type 1* (GGT1) is an enzyme localized on the surface of almost all types of epithelial cells. It plays a critical role in the regulation of levels of reactive oxygen species (ROS) by maintaining the balance between reduced glutathione and its oxidized form [269]. The *GGT1* gene is expressed at a high level in the pancreas; however, the highest expression of *GGT1* is detected in the liver (Supplementary Table S1). It is noteworthy that an increase in plasma level of gamma-glutamyltransferase is a common finding in T2D [270,271]. A few studies have been done to assess the relationships between the SNPs of *GGT1* gene and the risk of type 2 diabetes. Lee with colleagues observed a weak association between the rs4820599 SNP and the risk of type 2 diabetes [272]. Jinnouchi et al. did not find an association between SNP rs4820599 and disease risk, but it was revealed that this polymorphism was associated with the risk of diabetic retinopathy [273]. Studies of Diergaarde B. with colleagues [274] and Brand with colleagues [275] have identified an association of the rs4820599 polymorphism with the risk of pancreatic cancer and chronic pancreatitis, respectively. Two large studies have established the effect of rs4820599 polymorphism on the level of gamma-glutamyltransferase in blood [276,277]. The rs5751909-A allele is known to be associated with increased levels of *GGT1* mRNA in the pancreas and decreased levels in the blood (Supplementary Table S1).

*Gamma-glutamyl transpeptidase type 5* (GGT5) is an enzyme which cleaves the gamma-glutamyl peptide bond of glutathione and glutathione S-conjugates such as leukotriene C4 [278]. It is known that GGT5 converts C4 leukotriene to D4 leukotriene, which increases smooth muscle tone and promotes plasma exudation in tissues [279]. The *GGT5* gene is expressed in almost all tissues and organs, but to the greatest extent in adipose tissue, kidneys, thyroid gland, peripheral nerves, arteries, and cardiac muscle (Supplementary Table S1). A few studies investigated the association of polymorphisms of the *GGT5* gene with human traits or diseases. Astle with colleagues found that the rs2275984-C allele was associated with an increase in granulocytes and monocytes in the white blood cells [280]. In whole blood, the rs2275984-C allele is related with lower expression of glutathione metabolism genes *GGT5, GGT1*, and *GSTT1*, as well as *UPB1*, *DDT, SUSD2*, and *SPECC1L* genes, and higher expression of the *ADORA2A* gene (Supplementary Table S2). All the above genes are located in the 22q11.23 chromosomal segment, and their co-expression seems to be under the control of common *cis*-regulatory elements-enhancers located in this region, as can be seen from the GeneHancer data available at https://www.genecards.org (accessed on 2 December 2022).

*Gamma-glutamyl transpeptidase type 6* (GGT6) is a membrane-bound extracellular enzyme which cleaves gamma-glutamyl peptide bonds in glutathione and transfers it to gamma-glutamyl acceptors [184,185]. Like other gamma-glutamyltransferases, GGT6 is a key regulator of glutathione homeostasis by providing cells with amino acid substrates for GSH biosynthesis [281]. To date, no genetic association studies have been undertaken to investigate the association of *GGT6* gene polymorphisms and susceptibility to type 2 diabetes and other diseases. 

*Gamma-glutamyl transpeptidase 7* (GGT7) is the extracellular membrane-bound enzyme, also known as gamma-glutamyl transferase 7, that breaks gamma-glutamyl peptide bonds in the GSH molecule and transfers gamma-glutamyl compounds to acceptors [281]. GGT7, like other gamma-glutamyltransferases, is essential for maintaining glutathione homeostasis by supplying substrates for glutathione synthesis, particularly in tissues like the pancreas that produce glutathione at a low rate. However, the enzyme mitigates oxidative damage by degrading extracellular glutathione [282]. This pathway, known as the gamma-glutamyl cycle, allows cells to use the released amino acids for *de novo* GSH synthesis [184]. No genetic association studies have been done to assess the association of *GGT7* gene polymorphisms with the risk of any disease. We found in our recent study [240] that the rs6119534-T allele of the *GGT7* gene is strongly associated with a decreased risk of T2D, especially among individuals without T2D-related risk factors such as physical inactivity, smoking, stress, insufficient consumption of fresh fruits/vegetables and proteins, excessive consumption of carbohydrates, and low-fiber foods. The rs6119534-T allele is known to be associated with an increased level of the *GGT7* and *GSS* genes in skeletal muscles (Supplementary Table S2). Surprisingly, none of the SNPs are associated with the level of *GGT7* gene expression in the pancreas. Like *GSS*, the *GGT7* gene is expressed at a low level in the pancreas compared to other organs and tissues [283,284].

**Table 1:** Genetic association studies of enzymes involved in the regulation of redox homeostasis in type 2 diabetes mellitus.

Gene	Gene Name	Polymorphism/ SNP ID	Genotype/ Allele	Odds Ration [95% CI]	Cofactor	Population	Reference
*GSTM1*	glutathione S-transferase M1	Deletion	del/del	1.99 [1.30–3.05]	males	European (Russians)	[282]
			del/del	1.99 [1.46–2.71]	-	Chinese Turkish Japanese Indians Taiwanese Iranians Egyptians	[283]
			del/del	2.90 [1.76–4.78]	-	Indians	[284]
			del/del	2.042 [1.254–3.325]	-	Indians	[285]
			del/del	3.841 [2.280–6.469]	-	Turkish	[286]
			del/del	1.74 [1.13–2.69]	-	Iranians	[287]
*GSTT1*	glutathione S-transferase T1	Deletion	del/del	2.23 [1.22–4.09]	males	European (Russians)	[282]
			del/del	1.61 [1.19–2.17]	-	Chinese Turkish Japanese Indians Taiwanese Iranians Egyptians	[283]
			del/del	2.90 [1.76–4.78]	-	Indians	[284]
*GSTP1*	glutathione S-transferase P1	rs1138272	114A/V	2.85 [1.44–5.62]	males	European (Russians)	[282]
		rs1695	105I/V	1.99 [1.20–3.32]	-	Romanian	[288]
		rs1695	105I/V	2.56 [1.47–4.48]	-	Indians	[284]
		rs1695	105I/V	0.397 [0.225–0.701]	-	Indians	[285]
*GCLC*	glutamate cysteine ligase catalytic subunit	rs12524494	G	0.62 [0.41–0.93]	Nonsmokers	European (Russians)	[289]
*GCLM*	glutamate cysteine ligase modifier subunit	rs3827715	C	0.86 [0.75–0.99]	-	European (Russians)	[289]
		rs41303970	A	0.77 [0.63–0.93]	Nonsmokers	European (Russians)	[289]
*GSS*	glutathione synthetase	rs13041792	A	1.14 [1.01–1.29]	-	European (Russians)	[178]
*GSR*	glutathione reductase	rs2551715	T/T	0.33 [0.13–0.82]	BMI < 25 kg/m^2^ Daily consumption of fresh fruits and vegetables	European (Russians)	[290]
*GGT7*	gamma-glutamyl transferase 7	rs11546155	A/A	0.42 [0.22–0.80]	-	European (Russians)	[178]
		rs6119534	T	0.85 [0.76–0.95]	-	European (Russians)	[178]
*GGCT*	gamma-glutamyl cyclotransferase	rs4270	T/C-C/C	0.71 [0.54–0.93]	Nonsmokers; Daily consumption of fresh fruits and vegetables	European (Russians)	[291]
*GPX1*	glutathione peroxidase 1	rs1050450	T/T	1.76 [1.011–3.066]	-	South Indian	[292]
*GPX2*	glutathione peroxidase 2	rs4902346	G/G	1.41 [1.02–1.96]	Males	European (Russians)	[293]
*CAT*	catalase	rs769217	T	2.94 [1.66–5.23]	-	Egyptians	[294]
*SOD1*	Superoxide dismutase 1	rs2234694	C	2.9 [1.84–4.6]	-	Egyptians	[294]
*SOD2*	superoxide dismutase 2	rs4880	C	2.434 [1.413–4.191]	-	North Indian	[145]
*SOD3*	superoxide dismutase 3	rs2536512	GA-AA	1.64 [1.16–2.33]	-	Chinese	[295]
*RAC1*	Rac family small GTPase 1	rs7784465	T/C	1.40 [1.20–1.65]	Dietary deficit of fresh fruits and vegetables; Excess of carbohydrates in food; High calorie diet; Psychological stress; Sedentary lifestyle	European (Russians)	[296]
*CYBA*	cytochrome b-245 alpha chain	rs4673	A/A	1.60 [1.04–2.46]	Females	European (Russians)	[297]
			T/T	1.74 [1.15–2.64]	-	Asians	[298]
			T	1.30 [1.04–1.61]	-	Non-Asians	
*CYBB*	cytochrome b-245 beta chain	rs5963327	T	1.7 [1.06–2.75]	Males	European (Russians)	[299]
		rs6610650	A	1.71 [CI 1.05–2.78]	Males	European (Russians)	[299]
		rs5963327	T/T	1.35 [1.05–1.73]	Females	European (Russians)	[299]
		rs6610650	A/A	1.34 [1.05–1.72]	Females	European (Russians)	[299]
*NCF2*	neutrophil cytosolic factor 2	rs17849502	G/T	1.42 [1.08–1.87]	BMI > 25 kg/m^2^	European (Russians)	[300]
*MPO*	Myeloperoxidase	rs2107545	T/C	1.563 [1.166–2.096]	-	Han Chinese	[301]

*Gamma-glutamyl cyclotransferase* (GGCT) is an enzyme catalyzing the formation of 5-oxoproline from gamma-glutamyl dipeptides. GGCT also causes the release of cytochrome *c* from mitochondria with subsequent induction of apoptosis [285]. No genetic association studies investigated the contribution of *GGCT* gene polymorphisms to the development of diabetes. Yu. A. Bocharova found that SNP rs6462210 was associated with a decreased risk of ischemic stroke [286]. In our recent study [258], carriage of the rs4270 T/C-C/C genotypes was associated with a decreased risk of T2D, but this association occurred only in those individuals who were non-smokers and consumed a sufficient amount (approximately 400 g daily) of fresh vegetables and fruits.

*Glutathione S-transferase pi 1* (GSTP1) is an enzyme conjugating a reduced glutathione with a large number of exogenous and endogenous hydrophobic electrophilic compounds [287,288] and is involved in the formation of glutathione conjugates of prostaglandins A2 and J2 [289]. The rs1695 (Ile105Val) polymorphism is the most studied variant of the *GSTP1* gene in a variety of human diseases such as type 2 diabetes [255], acute lymphoblastic leukemia [290], Hodgkin’s lymphoma [291], breast cancer [292], lung cancer [293], acute pancreatitis [294], Alzheimer’s disease [295], and bronchial asthma [296]. It is also known that another well-studied polymorphism rs1138272 (Ala114Val) of the *GSTP1* gene is associated with the risk of T2D [249], bronchial asthma [297], cancers [298], and Parkinson’s disease [299].

*Glutathione peroxidase 1* (GPX1) is a member of the glutathione peroxidase family, which catalyzes the reduction of organic hydroperoxides and hydrogen peroxide (H_2_O_2_) by glutathione, protecting cells from oxidative damage [300]. A decrease in GPX activity in T2D was first noted in rats with streptozotocin-induced diabetes [300,301]. The literature on associations between SNPs in glutathione peroxidase genes and T2D risk is limited and contradictory. In particular, Tanaka [302] showed that an increase in GPX expression protected beta-cells from the damaging effects of oxidative stress during hyperglycemia, while McClung [303] reported that insulin resistance correlated with increased GPX1 expression. The role of glutathione peroxidase isoforms other than GPX1 in the development of diabetes remains unknown. Huang et al. [304] showed that knockout and overexpression of *Gpx1* in mice may induce types 1 and 2 diabetes-like phenotypes. Another study [305] discovered that *GPX1* gene polymorphisms protected the kidneys from oxidative damage in type 1 diabetic patients. Liu D. et al. [306] discovered that the *GPX1* polymorphism rs1050450 was associated with an increased risk of carotid plaques in T2D patients. In ApoE/mice, a lack of functional Gpx1 accelerated diabetes-associated atherosclerosis by upregulating proinflammatory and profibrotic pathways [307]. 

*Glutathione peroxidase 2* (GPX2) is a member of the glutathione peroxidase family, which encodes a selenium-dependent enzyme responsible for the majority of the glutathione-dependent hydrogen peroxide-reducing activity in the epithelial cells of the gastrointestinal tract. *GPX2* is characterized by the highest level of expression in the pancreas according to the data from the BioGPS project [308]. GPX2 is active in the cytosol and mitochondrial matrix where the enzyme protects all parts of the cell from the damaging effects of ROS. Substrates for GPX2 include not only hydrogen peroxide and lipid hydroperoxides, but also peroxynitrite. We observed an association between the rs4902346 polymorphism in the intron of the *GPX2* gene and an increased risk of T2D [260]. The minor allele rs4902346-G of *GPX2* is known to be associated with a decrease in the expression of the *GPX2* gene in the liver, the small intestine, subcutaneous and visceral adipose tissues, nervous tissue and skeletal muscle [309].

## 8. Genes for ROS-Generating Enzymes and Susceptibility to Type 2 Diabetes

Reactive oxygen and nitrogen (RNS) species, formerly believed to be by-products of cellular metabolism, are known to play important regulatory roles in a number of vital physiological processes, including cell survival, proliferation, differentiation, migration, and adhesion [310]. Superoxide anion and hydrogen peroxide produced by NADPH oxidase (NOX) play a significant role in numerous cellular signaling networks [311]. Currently, seven NOX isoenzymes are known, with tissue-specific expression patterns: NOX1, NOX2, NOX3, NOX4, NOX5, DUOX1, and DUOX2. The main subunits of NADPH oxidase are cytochrome b-245 light chain (CYBA), cytochrome b-245 heavy chain (CYBB), neutrophil cytosolic factor 1 (NCF1), neutrophil cytosolic factor 2 (NCF2), neutrophil cytosolic factor 4 (NCF4), and small GTPases (RAC1/RAC2). Cytosolic protomers such as NCF1, NCF2, and NCF4 provide activation of two transmembrane domains CYBA and CYBB, which form the catalytic core of the multienzyme complex of NADPH oxidase [311]. This complex catalyzes the one-electron reduction of molecular oxygen to superoxide, which is essential for oxidizing pathogens [312]. All ROS-producing NOX isoforms may transport electrons across membranes and produce superoxide anion and/or hydrogen peroxide, which are signaling molecules impacting on all facets of cell activity [313]. As can be seen from Table 1, several genetic studies showed that polymorphic genes encoding oxidative enzymes are associated with the risk of T2D. These polymorphisms are rs7784465 of *RAC1* [253], rs4673 of *CYBA* [314,315], rs5963327 and rs6610650 of *CYBB* [316], rs17849502 of *NCF2* [317], and rs2107545 of *MPO* [306]. Below, we describe the results of studies that observed associations between polymorphic genes for oxidative enzymes and T2D susceptibility.

*Cytochrome b-245 alpha chain* (CYBA) is a critical component of the membrane-bound phagocyte oxidase, which, by binding to CYBB, forms a functionally active NADPH oxidase. Several SNPs were identified in the *CYBA* gene; for instance, –242C>T (rs4673) in the fourth exon, resulting in amino acid substitution His72Tyr, 640A>G (rs1049255) in the 3-prime-untranslated region, and –930A>G (rs9932581) located in the promoter gene region at the binding site for transcription factor CEBP, affecting transcriptional activity of the *CYBA* gene [318]. There is experimental evidence that the 242C>T and 640A>G polymorphisms of the *CYBA* gene affect the binding affinity of the p22phox and gp91phox protein subunits, modulating the activity of NADPH oxidase and the generation of superoxide anions [319,320]. A study by Meijles and colleagues showed that the 242C>T polymorphism caused structural changes in p22phox that inhibited the activation of endothelial NOX2 and the oxidative response to tumor necrosis factor alpha or glucose stimulation [321]. It is known from the literature that the rs4673C allele (polymorphism C242T) is associated with endothelial dysfunction in patients with type 2 diabetes [311], the risk of coronary artery disease [322], T2D [315], peripheral neuropathy in type 1 diabetes mellitus [323], hypertension in patients with type 2 diabetes [324], lung cancer [325], bronchial asthma [326], and some other diseases. It has also been found that the rs4673-A/G genotype, compared to the wild-type G/G genotype, of the *CYBA* gene is associated with more pronounced liver damage in patients with viral hepatitis B [264]. Our recent study showed a sex-specific association of the *CYBA* rs4673 polymorphism with the risk of type 2 diabetes in women [314]. It has been suggested that the rs9932581 variant of *CYBA* modulates the transcriptional activity of the gene promoter through allele-specific binding to CEBP (the −930G allele increases the affinity for this transcription factor to interact with the gene promoter) [327]. In particular, functional studies showed that the promoter with the −930G allele variant had a 30% higher expression of the *CYBA* gene than the promoter with the −930A allele [318]. It was also found that the −930G allele was associated with increased ROS production [327,328]. SNP rs9932581 is associated with premature development of coronary atherosclerosis [329] and hypertension [318,328]. In addition, SNP rs9932581 is associated with an increased risk of renal complications in patients with type 1 diabetes [330]. 

*Cytochrome b-245 beta chain* (CYBB) is an essential component of membrane-bound phagocyte oxidase that produces the superoxide anion. CYBB is the terminal component of the respiratory chain that transfers electrons from the cytoplasmic portion of NADPH oxidase across the plasma membrane to molecular oxygen outside. In addition, CYBB functions as a voltage-gated proton channel that transmits H+ fluxes in inactive phagocytes and is involved in the regulation of cellular pH. CYBB expression is regulated by the nuclear transcription factor NF-κB [331]. The *CYBB* gene is located at chromosome X. According to the DisGeNET database, polymorphisms in the *CYBB* gene have been the subject for genetic association studies in hematological and infectious diseases. In particular, the rs6610650A allele of the *CYBB* gene is associated with a decreased risk of tuberculosis in male smokers [332]. Our study revealed sex-specific associations of rs6610650 and rs5963327 with fasting hyperglycemia and increased risk of T2D in males in the presence of risk factors such as cigarette smoking and diets with excess calories and sugar [316]. Furthermore, the above-mentioned SNPs were related with a predisposition to T2D in females, whereas rs5917471 of *CYBB* was associated with fasting hyperglycemia in males [316] but not with T2D.

*Neutrophil cytosolic factor 2* (NCF2) is a neutrophil cytosolic factor (also known as p67phox), which is part of the NADPH oxidase enzyme complex and is responsible for its activation by binding to the CYBA and CYBB subunits [320]. Few studies have been done to investigate the association of *NCF2* gene polymorphisms with human diseases. It is known that rs10911363 and rs17849502 *NCF2* are associated with the risk of systemic seropositive rheumatic disease [333], rheumatoid arthritis [334], and arthritis in patients with systemic lupus erythematosus [335]. Our recent study showed that SNP rs17849502 of *NCF2* was associated with an increased risk of T2D in overweight and obese patients [317].

*Neutrophil cytosolic factor 4* (NCF4) is also known as p40phox and together with other proteins (NCF4, CYBA, and CYBB) is involved in activation of NADPH oxidase [336]. SNP rs4821544 *NCF4* is associated with the risk of rheumatoid arthritis [337] and Crohn’s disease [338], whereas rs5995355 is associated with the risk of colorectal cancer [339]. Xing and colleagues [340] found an increase in NCF4 expression in the neutrophils of patients with latent adult autoimmune diabetes (LADA), which contributed to oxidative damage to pancreatic beta-cells. Our recent study showed no significant associations of *NCF4* polymorphisms such as rs5995355, rs5995357, rs1883112, rs4821544, rs760519, rs729749, rs2075938, and rs2075939 with the risk of T2D [341]. Nevertheless, we revealed associations of the rs4821544-C/C and rs5995357-A/A genotypes with increased risk of coronary artery disease in females with T2D [341]. In addition, carriers of the rs4821544-C/C genotype had significantly higher levels of glycated hemoglobin and oxidized glutathione. 

*NADPH oxidase 1 activator* (NOXA1) is a functional homologue of p67phox for the activation of NADPH oxidase in vascular smooth muscle cells (SMCs) and plays an important role in atherogenesis [291,342]. NOXA1 is capable of activating CYBB/gp91phox and NOX3 [343]. There is no data on the association of *NOXA1* gene variants with T2D risk. 

*NADPH oxidase 1* (NOX1) is a homologue of the catalytic subunit of the superoxide-generating phagocyte NADPH oxidase, gp91phox. The oxidase activity of the enzyme is regulated by NOXA1 and NOXO1 [344]. As can be seen from the DisGeNET database, association studies of *NOX1* gene polymorphisms were carried out on cardiovascular diseases [345]. 

*NADPH oxidase 4* (NOX4) was originally identified as a homologue of NADPH oxidase highly expressed in the kidney [346]. In addition to the kidneys, NOX4 is expressed in smooth muscle cells and vascular endothelium, has protective properties through the modulation of endothelial nitric oxide synthase, and is also characterized by anti-inflammatory and anti-apoptotic effects [347]. It has been established that polymorphisms rs3913535, rs10765219, and rs11018670 of the *NOX4* gene are associated with the risk of severe diabetic retinopathy [348]. The rs2164521 variant of *NOX4* is associated with a decreased risk of hepatopulmonary syndrome [349]. 

*NADPH oxidase 5* (NOX5) from the NOX family is unique in that it does not require NADPH oxidase subunits for its activation [350]. NOX5 is localized in the regions of the perinuclear and endoplasmic reticulum of cells and, after activation, is directed to the cell membrane. Vasoactive substances, growth factors, and pro-inflammatory cytokines all activate NOX5 and modulate its activity by a variety of post-translational changes [350]. NOX5 hyperactivation is linked to the onset of cardiovascular disease, kidney damage, and cancer. However, the exact pathophysiological functions of NOX5 are still unknown [350]. NOX5 was found to be activated in human diabetic nephropathy and affects the function of the filtration barrier and blood pressure through excessive formation of ROS [351]. Expression of human NOX5 in vascular smooth muscle cells and mesangial cells in mice has been shown to induce oxidative stress, glomerulosclerosis, mesangial expansion, inflammation, and fibrosis in the kidneys: processes that are known to accelerate the progression of renal failure in diabetes [352]. It is known from the literature that the *NOX5* gene is also associated with Hirschsprung’s disease [353].

*Rac family small GTPase 1* (RAC1) belongs to the Rho family of GTPases that regulates the redox state of the cell by controlling enzymes generating and converting reactive oxygen and nitrogen species [310]. RAC1 is an integral part of the NOX2 holoenzyme. Activation of RAC1 is required for the subsequent generation of ROS. Indeed, RAC1 is directly involved in the assembly and activation of NADPH oxidases (NOX1/2/3), which generate superoxide anion radicals, which in turn activate nuclear factor-κB (NFκB), a transcription factor regulating the expression of redox homeostasis genes such as NOX2 and superoxide dismutase 2 [310]. While NOX2 increases ROS generation, SOD2 converts highly reactive oxygen radicals into less reactive hydrogen peroxide. When redox homeostasis is shifted to the reducing state, ROS activate RAC1 and RhoA, whereas under oxidative stress, these GTPases become inactive [310]. The depletion of intracellular glutathione leads to an increase in active forms of RAC1, replenishing the GSH deficit in the cell by the antioxidant N-acetylcysteine blocks RAC1 activation [354]. Notably, acylation of RAC1 at the amino acid residue Cys178 is a critical process for RAC1-mediated remodeling of the actin cytoskeleton [355], which is important in the regulation of transmembrane glucose transport [356] and insulin secretion in pancreatic beta-cells in T2D [357]. The suppression of RAC1 protects β-cells from the damaging effects of glucose, fatty acids, and pro-inflammatory cytokines [358,359,360]. Interestingly, one of the main stages in the development of metabolic dysregulation of islet beta-cells in type 2 diabetes is thought to be sustained activation of RAC1 [361]. It is important to note that chronic activation of RAC1 has been found in many diseases, including cancer, neurodegenerative diseases (Parkinson’s and Alzheimer’s diseases), and cardiometabolic disorders, including type 2 diabetes [362,363].

In our recent study, we discovered that the RAC1 gene polymorphism rs7784465 was associated with the risk of T2D in individuals with a lower daily intake of fresh fruits and vegetables, as well as those who experienced psycho-emotional stress and physical inactivity [314]. rs10951982, another *RAC1* gene polymorphism, was found to be protective against T2D risk in subjects who did not abuse food with excessive amount of carbohydrates. It was also revealed that another SNP, rs836478 of *RAC1,* was associated with elevated levels of glycated hemoglobin and fasting blood glucose. In addition, the *RAC1* gene polymorphisms were associated with increased plasma levels of hydrogen peroxide and uric acid in T2D patients [364]. Few studies have been undertaken to assess the association between *RAC1* gene polymorphisms and other human diseases. A meta-analysis of genome-wide association studies showed that SNP rs7784465 was associated with body mass index [365]. 

*Rac family small GTPase 2* (RAC2), like RAC1, belongs to the family of Rho GTPases that regulates cellular redox homeostasis. RAC2 is involved in the activation of NADPH oxidase (NOX2), increasing ROS production [366]. The active form of RAC2 binds to various effector proteins that regulate a variety of cellular processes, such as secretion, phagocytosis of apoptotic cells, and polarization of epithelial cells [367]. RAC2 interacts with inducible NO synthase, producing nitric oxide [368]. According to the DisGeNET database, a limited number of genetic association studies have been done to explore the association between *RAC2* gene polymorphisms and the risk of oncological, inflammatory, and hematological diseases. 

In summarizing the findings of genetic studies, it should be noted that a significant proportion of established associations between redox homeostasis gene polymorphisms and type 2 diabetes have weak or moderate effects on disease risk, and thus these findings require confirmation in independent populations as well as experimental functional annotation of disease-associated alleles.

## 9. Conclusions

Numerous studies have demonstrated that individuals with type 2 diabetes exhibit impaired redox homeostasis and oxidative stress attributed to, on the one hand, a deficiency in the production of endogenous antioxidants, mainly glutathione, and, on the other hand, an excess of free radicals. In type 2 diabetes, increased levels of reactive oxygen species and oxidative stress were found to be associated with pathological conditions, such as beta-cell dysfunction and insulin resistance. Patients with T2D have lower levels of reduced glutathione and a slower rate of its biosynthesis than healthy individuals, and the decreased level of glutathione correlated with increased blood glucose levels and disease progression. Glutathione has also been demonstrated to inhibit beta-cell dedifferentiation and failure caused by chronic oscillating glucose consumption [34].

**Figure 1:**
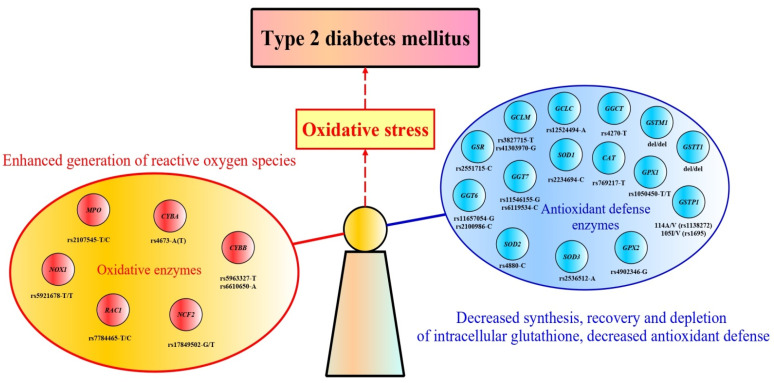
The relationship between genes encoding enzymes involved in the regulation of redox homeostasis and the development of type 2 diabetes. The scheme reflects the genetic causes responsible for oxidative stress in type 2 diabetes as a result of an imbalance between the generation of free radicals and their neutralization, which is attributed to the impact of polymorphisms in the genes encoding oxidative and antioxidant defense enzymes. Genotypes and alleles of the increased disease risk are shown along the genes. GSTM1, glutathione S-transferase M1; GSTT1, glutathione S-transferase T1; GSTP1, glutathione S-transferase P1; GCLC, glutamate cysteine ligase catalytic subunit; GCLM, glutamate cysteine ligase modifier subunit; GSS, glutathione synthetase; GSR, glutathione reductase; GGT6, gamma-glutamyl transferase 6; GGT7, gamma-glutamyl transferase 7; GGCT, gamma-glutamyl cyclotransferase; GPX1, glutathione peroxidase 1; GPX2, glutathione peroxidase 2; CAT, catalase; SOD1, Superoxide dismutase; SOD2, superoxide dismutase 2; SOD3, superoxide dismutase 3; RAC1, Rac family small GTPase 1; CYBA, cytochrome b-245 alpha chain; CYBB, cytochrome b-245 beta chain; NCF2, neutrophil cytosolic factor 2; NOX1, NADPH oxidase1; MPO, Myeloperoxidase.

Polymorphisms at genes encoding antioxidant defense and oxidative enzymes have the potential to impact redox homeostasis and therefore they represent attractive markers for testing the genetic susceptibility to type 2 diabetes. Several studies have been done to evaluate whether polymorphisms in genes for glutathione S-transferases, superoxide dismutases, glutathione peroxidases, cytochrome b-245 alpha chain, and myeloperoxidase are associated with T2D susceptibility. These studies showed that variants at the oxidative stress-related genes were significantly associated with diabetes risk. Meanwhile, a limited number of studies, a significant proportion of which were carried out by our research team, looked into the link between T2D and genetic variations in glutathione-metabolizing enzymes such as glutamate cysteine ligase, glutathione synthetase, glutathione reductase, gamma-glutamyl cyclotransferase, and gamma-glutamyltransferases. Loss of function variants at genes encoding glutathione metabolizing enzymes, together with environmental factors, contribute to an intracellular glutathione deficiency in type 2 diabetes. The mechanism by which genes encoding enzymes involved in the regulation of redox homeostasis contribute to the development of type 2 diabetes mellitus is summarized in Figure 1.

The most significant contribution to T2D predisposition is attributed to polymorphisms of genes such as *GCLM, GSS, GGT1, GGT7, GSTM1, GSTT1, GSTP1, GPX2, RAC1, CYBA, CYBB,* and *NCF2*. The effects of each gene polymorphism on redox homeostasis and disease risk may vary significantly across populations, and this variation is attributed to interpopulation differences in minor allele frequencies and linkage disequilibrium between SNPs, as recently demonstrated in a number of studies [240,369,370]. The link between polymorphic genes involved in redox homeostasis, such as *GCLM* (rs2301022), *GGT7* (rs6119534, rs11546155), *GSTM1* (+/del), *GSTP1* (rs1695, rs1138272), *RAC1* (rs7784465), and *CYBB* (rs5917471), and type 2 diabetes differs in overweight and normal-weight individuals. The impact of genetic variants of these enzymes on disease risk is triggered by environmental factors such as insufficient intake of proteins, fresh vegetables and fruits, as well as smoking and physical inactivity. Further research is needed to uncover the molecular mechanisms by which interactions between genetic and environmental factors contribute to type 2 diabetes pathogenesis via alterations in redox homeostasis. In particular, the pathophysiological relationship between glutathione deficiency and T2D development could be explained not only by the development of oxidative stress but also by other mechanisms. Since T2D-associated alleles of genes for glutathione metabolism (e.g., GSS and GGT7) correlate with expression levels of genes involved in the unfolded protein response pathway and regulation of proteostasis [240], it can be hypothesized that glutathione deficiency in beta-cells of pancreatic islets may contribute to the impaired folding of proinsulin identified in type 2 diabetes.

Understanding the importance of redox homeostasis in the pathogenesis of type 2 diabetes substantiates the need for antioxidant treatment focusing on replenishing the endogenous glutathione pool, and several clinical studies have already shown the efficacy and safety of this approach in diabetics [371,372,373]. However, this type of therapy for patients with type 2 diabetes has not been implemented into endocrinological practice or included in any country’s national clinical recommendations.

## Supplementary Materials

The following supporting information can be downloaded at: https://www.mdpi.com/article/10.3390/ijms24054738/s1.
