# Supplementary material for: Molecular Genetics of Abnormal Redox Homeostasis in Type 2 Diabetes Mellitus
Source: Int J Mol Sci. 2023 Mar 1;24(5):4738. doi: 10.3390/ijms24054738 (PMC10003739; doi:10.3390/ijms24054738)
Supplement: Supplementary file 1 [file ijms-24-04738-s001.zip › Supplementary Table S1.pdf]

Suppl. Table S1. Biological functions and tissue-specific expression of genes encoding enzymes for redox homeostasis

Biological functions and tissue-specific gene expression of antioxidant enzymes								
Approved gene symbol[1] (Gene ID)[2] Chromosomal location[2]	Approved gene name[1] (protein name)[3]	Enzyme/protein function[3]	Basic function[4]	Tissue gene expression[5]				
				Pancreas	Skeletal muscle	Adipose tissue	Liver	Whole blood
GSTM1 (2944) 1p13.3	glutathione S-transferase mu 1	Conjugation of reduced glutathione to a wide number of exogenous and endogenous hydrophobic electrophiles. Involved in the formation of glutathione conjugates of both prostaglandin A2 (PGA2) and prostaglandin J2 (PGJ2). Participates in the formation of novel hepoxilin regioisomers.	• conjugating reduced glutathione to a wide number of exogenous and endogenous hydrophobic electrophiles<br>• playing a role in telomere attrition and subsequent telomerase activity in the cancer cells<br>• absence of GSTM1 activity can be compensated for by the overexpression of GSTM2	+	+	+	+++	+
GSTM2 (2946) 1p13.3	glutathione S-transferase mu 2	Conjugation of reduced glutathione to a wide number of exogenous and endogenous hydrophobic electrophiles. Participates in the formation of novel hepoxilin regioisomers.	• conjugating reduced glutathione to a wide number of exogenous and endogenous hydrophobic electrophiles<br>• GSTM2-2 is a novel luminal regulator of the RyR channels in the heart<br>• absence of GSTM1 activity can be compensated for by the overexpression of GSTM2	+	++	+	+++	+
GSTM3 (2947) 1p13.3	glutathione S-transferase mu 3	Conjugation of reduced glutathione to a wide number of exogenous and endogenous hydrophobic electrophiles. May govern uptake and detoxification of both endogenous compounds and xenobiotics at the testis and brain blood barriers.	• establishment of blood/nerve barrier<br>• conjugating reduced glutathione to a wide number of exogenous and endogenous hydrophobic electrophiles	++	+	++	++	-

GSTM4 (2948) 1p13.3	glutathione S-transferase mu 4	Conjugation of reduced glutathione to a wide number of exogenous and endogenous hydrophobic electrophiles. Catalyzes the conjugation of leukotriene A4 with reduced glutathione (GSH) to form leukotriene C4. Can also catalyzes the transfer of a glutathionyl group from glutathione (GSH) to 13(S),14(S)-epoxy-docosahexaenoic acid to form maresin conjugate in tissue regeneration 1 (MCTR1), a bioactive lipid mediator that possess potent anti-inflammatory and proresolving actions.	• playing a role in proliferation and oncogenic transformation is likely specific to Ewing's sarcoma	++	++	++	++	++
GSTM5 (2949) 1p13.3	glutathione S-transferase mu 5	Conjugation of reduced glutathione to a wide number of exogenous and endogenous hydrophobic electrophiles.	• glutathione-S-transferase	++	++	+	++	++
GSTT1 (2952) 22q11.23	glutathione S-transferase theta 1	Conjugation of reduced glutathione to a wide number of exogenous and endogenous hydrophobic electrophiles. Acts on 1,2-epoxy-3-(4-nitrophenoxy)propane, phenethylisothiocyanate 4-nitrobenzyl chloride and 4-nitrophenethyl bromide. Displays glutathione peroxidase activity with cumene hydroperoxide.	• involved in tautomerization of d-dopachrome with decarboxylation to give 5,6-dihydroxyindole<br>• catalyzing the conjugation of reduced glutathione to a variety of electrophilic and hydrophobic compounds	++	++	+++	+	+

GSTT2<br>(2953)<br>22q11.23	glutathione S-transferase theta 2	Conjugation of reduced glutathione to a wide number of exogenous and endogenous hydrophobic electrophiles. Has a sulfatase activity.	• involved in the conjugation of reduced glutathione to a wide number of exogenous and endogenous hydrophobic electrophiles<br>• playing an important role in human carcinogenesis<br>• acting as a sulfatase<br>• participating in detoxification and defense mechanisms against toxic carcinogens and other compounds	++	++	+	++	++

GSTP1 (2950) 16p13.13	glutathione S-transferase pi 1	Conjugation of reduced glutathione to a wide number of exogenous and endogenous hydrophobic electrophiles. Involved in the formation of glutathione conjugates of both prostaglandin A2 (PGA2) and prostaglandin J2 (PGJ2). Participates in the formation of novel hepoxilin regioisomers. Regulates negatively CDK5 activity via p25/p35 translocation to prevent neurodegeneration.	• is a nucleo-cutoplasmic shuttling protein<br>• involved in regulation of mammalian cell growth<br>• mediating translation termination, and controling the formation of the termination complex by modulating ETF1 protein stability<br>• GSPT1 is the major factor acting in translation termination in mammals and GSPT2 can substitute for GSPT1 in this function<br>• is a GTPase associated with ETF1 in a complex that mediates translation termination in eukaryotes<br>• eukaryotic translation termination is mediated by two interacting release factors, ETF1 and GSPT1, which act cooperatively to ensure efficient stop codon recognition and fast polypeptide release<br>• ETF1 recognizes the stop codon in the A site of the ribosome and promotes nascent peptide chain release, and the GTPase GSPT1 facilitates this peptide release via its interaction with ETF1<br>• plays pivotal roles in translation termination and post-termination events including ribosome recycling and mRNA decay<br>• could serve as a target in the regulation of gene expression<br>• functional interplay between two release factors, ETF1 and GSPT1, involved in eukaryotic translation termination, and GTP hydrolysis by GSTP1 couples codon recognition with peptidyl-tRNA hydrolysis by ETF1<br>• is a multifunctional protein that plays pivotal roles in translation termination as well as the initiation of mRNA decay, and also functions in the regulation of apoptosis<br>• is a novel regulator of cell death	++	++	++	-	++

GSTK1 (373156) 7q34	glutathione S-transferase kappa 1	Glutathione S-transferase that catalyzes the conjugation of glutathione to exogenous and endogenous compounds. Significant glutathione conjugating activity is found only with the model substrate, 1-chloro-2,4-dinitrobenzene (CDNB)	• may have glutathione conjugating activity	+	+	++	++	++
GSTA1 (2938) 6p12.2-p12.1	glutathione S-transferase alpha 1	Glutathione S-transferase that catalyzes the nucleophilic attack of the sulfur atom of glutathione on the electrophilic groups of a wide range of exogenous and endogenous compounds (Probable). Involved in the formation of glutathione conjugates of both prostaglandin A2 (PGA2) and prostaglandin J2 (PGJ2). It also catalyzes the isomerization of D5-androstene-3,17-dione (AD) into D4-androstene-3,17-dione and may therefore play an important role in hormone biosynthesis. Through its glutathione-dependent peroxidase activity toward the fatty acid hydroperoxide (13S)-hydroperoxy-(9Z,11E)-octadecadienoate/13-HPODE it is also involved in the metabolism of oxidized linoleic acid.	• conjugating reduced glutathione to a wide number of exogenous and endogenous hydrophobic electrophiles<br>• suppresses activation of JNK signalling by a pro-inflammatory cytokine and oxidative stress and have likely a protective role in JNK-associated apoptosis	++	++	+	++	++
GSTA2 (2939) 6p12.2	glutathione S-transferase alpha 2	Catalyzes the conjugation of glutathione to a large variety of electrophilic compounds.	• conjugating reduced glutathione to a wide number of exogenous and endogenous hydrophobic electrophiles			No Data		

GSTA3 (2940) 6p12.1	glutathione S-transferase alpha 3	Conjugation of reduced glutathione to a wide number of exogenous and endogenous hydrophobic electrophiles. Catalyzes isomerization reactions that contribute to the biosynthesis of steroid hormones. Efficiently catalyze obligatory double-bond isomerizations of delta5-androstene-3,17-dione and delta5-pregnene-3,20-dione, precursors to testosterone and progesterone, respectively. Has substantial activity toward aflatoxin B1-8,9-epoxide	• glutathione S-transferase activity<br>• may have a role in drug resistance<br>• play a role in cellular sex hormone production	++	++	+	+++	+
GSTA4 (2941) 6p12.1	glutathione S-transferase alpha 4	Conjugation of reduced glutathione to a wide number of exogenous and endogenous hydrophobic electrophiles. This isozyme has a high catalytic efficiency with 4-hydroxyalkenals such as 4-hydroxynonenal (4-HNE).	• glutathione S-transferase activity<br>• play an important role in the protection against oxidative stress	+	-	++	-	-
GSTA5 (221357) 6p12.1	glutathione S-transferase alpha 5	Catalytic Activity<br>glutathione + RX = a halide anion + an S-substituted glutathione + H+1	• unknown	++	++	++	+	++
GSTO1 (9446) 10q24.33	glutathione S-transferase omega 1	Exhibits glutathione-dependent thiol transferase and dehydroascorbate reductase activities. Has S-(phenacyl)glutathione reductase activity. Has also glutathione S-transferase activity. Participates in the biotransformation of inorganic arsenic and reduces monomethylarsonic acid (MMA) and dimethylarsonic acid.	• small stress response protein likely involved in cellular redox homeostasis<br>• implicated in the post-translational processing and activation of the proinflammatory mediator interleukin-1B<br>• inhibitor of cardiac muscle ryanodine receptor Ca2+ channels<br>• nuclear translocation could be potentially involved in the stress response of human cells playing a role in the cancer progression of Barrett esophagus<br>• two isozymes (GSTO1 and GSTO2) have significant DHA reductase (DHAR) activity<br>• participates in the glutathionylation cycle and targets specific proteins	++	-	++	+++	++

GSTO2 (119391) 10q25.1	glutathione S-transferase omega 2	Exhibits glutathione-dependent thiol transferase activity. Has high dehydroascorbate reductase activity and may contribute to the recycling of ascorbic acid. Participates in the biotransformation of inorganic arsenic and reduces monomethylarsonic acid (MMA).	• having glutathione transferase activity<br>• may play an important role in cellular signaling<br>• two isozymes (GSTO1 and GSTO2) have significant DHA reductase (DHAR) activity	++	++	++	+	++
GSTZ1 (2954) 14q24.3	glutathione S-transferase zeta 1	Bifunctional enzyme showing minimal glutathione-conjugating activity with ethacrynic acid and 7-chloro-4-nitrobenz-2-oxa-1,3-diazole and maleylacetoacetate isomerase activity. Has also low glutathione peroxidase activity with T-butyl and cumene hydroperoxides. Is able to catalyze the glutathione dependent oxygenation of dichloroacetic acid to glyoxylic acid.	• glutathione-S-transferase<br>• possessing glutathione peroxydase activity toward t-butyl and cumene hyperoxydes<br>• possessing maleylacetoacetate isomerase activity<br>• fundamental to the catabolism of aromatic AAs<br>• last enzyme from the phenylalanine and tyrosine catabolic pathway	+	++	++	+++	+
GCLC (2729) 6p12	Glutamate-cysteine ligase, catalytic subunit	Catalyzes the ATP-dependent ligation of L-glutamate and L-cysteine and participates in the first and rate-limiting step in glutathione biosynthesis. Catalytic Activity<br>ATP + L-cysteine + L-glutamate = ADP + gamma-L-glutamyl-L-cysteine + H+ + phosphate2	• catalyzing and limiting the first step of GSH synthesis, gamma-glutamyl cycle	+	++	+	++	+
GCLM (14630) 1p22.1	Glutamate-cysteine ligase, modifier subunit	Pathway - Sulfur metabolism; glutathione biosynthesis; glutathione from L-cysteine and L-glutamate: step 1/2.	• regulatory subunit, catalyzing the first step of GSH synthesis, gamma-glutamyl cycle<br>• implicated in some forms of hemolytic anemia	+++	++	++	++	++
GSS (2937) 20q11.21	Glutathione synthetase	Catalyzes the production of glutathione from gamma-glutamylcysteine and glycine in an ATP-dependent manner. Glutathione (gamma-glutamylcysteinylglycine, GSH) is the most abundant intracellular thiol in living aerobic cells and is required for numerous processes including the	• essential tripeptide antioxidant, that protects against reactive oxygen species and participates in metabolism of drugs and carcinogens<br>• involved in glutathione biosynthesis second step<br>• plays an important role in defending the cell against reactive oxygen species	++	+	+	++	+

		protection of cells against oxidative damage, amino acid transport, the detoxification of foreign compounds, the maintenance of protein sulfhydryl groups in a reduced state and acts as a cofactor for a number of enzymes.<br>Participates in ophthalmate biosynthesis in hepatocytes. Catalytic Activity<br>ATP + gamma-L-glutamyl-L-cysteine + glycine = ADP + glutathione + H+ + phosphate						
GSR<br>(2936)<br>8p21.1	Glutathione reductase	Maintains high levels of reduced glutathione in the cytosol. Catalytic Activity<br>2 glutathione + NADP+ = glutathione disulfide + H+ + NADPH	• glutathione reductase (NADPH) FAD dependent<br>• maintaining glutathione in the reduced state	++	++	+	++	++
GGT1<br>(2678)<br>22q11.23	Gamma-glutamyl transferase 1	Cleaves the gamma-glutamyl bond of extracellular glutathione (gamma-Glu-Cys-Gly), glutathione conjugates (such as maresin conjugate (13R)-S-glutathionyl-(14S)-hydroxy-(4Z,7Z,9E,11E,16Z,19Z)-docosahexaenoate, MCTR1) and other gamma-glutamyl compounds (such as leukotriene C4, LTC4).<br>The metabolism of glutathione by GGT1 releases free glutamate and the dipeptide cysteinyl-glycine, which is hydrolyzed to cysteine and glycine by dipeptidases.<br>In the presence of high concentrations of dipeptides and some amino acids, can also catalyze a transpeptidation reaction, transferring the gamma-glutamyl moiety to an acceptor amino acid to form a new gamma-glutamyl compound.<br>Contributes to cysteine homeostasis, glutathione homeostasis and in the conversion of the leukotriene LTC4 to LTD4. Catalytic Activity an alpha-amino	• transfer of the glutamyl moiety of glutamine to a variety of AAs and adipocytes acceptors	++	++	+	+++	++

		acid + an N-terminal (5-L-glutamyl)-[peptide] = 5-L-glutamyl amino acid + N-terminal L-alpha-aminoacyl-[peptide]						
GGT2 (830119) 22q11	Gamma-glutamyl transferase 2	Lacks catalytic activity due to its inability to undergo the autocatalytic cleavage needed to produce a mature, enzymatically active heterodimer.	• predictor of complications of atherosclerosis	++	++	+	+++	++
GGT3 (843318) 22q11.21	Gamma-glutamyl transferase 3	Hydrolyzes and transfers gamma-glutamyl moieties from glutathione and other gamma-glutamyl compounds to acceptors.	NG	+	++	+	+++	++
GGT5 (2687) 22q11.23	Gamma-glutamyl transferase 5	Cleaves the gamma-glutamyl peptide bond of glutathione and glutathione-S-conjugate such as leukotriene C4.<br>Does not cleaves gamma-glutamyl compounds such as gamma-glutamyl leucine.<br>May also catalyze a transpeptidation reaction in addition to the hydrolysis reaction, transferring the gamma-glutamyl moiety to an acceptor amino acid to form a new gamma-glutamyl compound.<br>Acts as a negative regulator of geranylgeranyl glutathione bioactivity by cleaving off its gamma-glutamyl group, playing a role in adaptive immune responses	• gamma-glutamyltransferase-like 1 activity<br>• hydrolizing the gamma-gmutamyl moiety of glutathione<br>• cleaving the gamma-glutamyl linkage of leukotriene C4 and most likely, other naturals compounds	++	++	++	++	++
GGT6 (124975) 17p13.2	Gamma-glutamyl transferase 6	Hydrolyzes and transfers gamma-glutamyl moieties from glutathione and other gamma-glutamyl compounds to acceptors.	• having gammaglutamyl transferase activity	++	++	++	+	++
GGT7 (2686) 20q11.22	Gamma-glutamyl transferase 7	Hydrolyzes and transfers gamma-glutamyl moieties from glutathione and other gamma-glutamyl compounds to acceptors.	• playing a key role in the gamma-glutamyl cycle, a pathway for the synthesis and degradation of glutathione	++	++	++	+	++
GGCT (79017)	Gamma-glutamyl cyclotransferase	Catalyzes the formation of 5-oxoproline from gamma-glutamyl dipeptides and	• catalyzes the formation of 5-oxoproline (pyroglutamic acid) from gamma-glutamyl	++	-	++	+	+

7p15-p14		may play a significant role in glutathione homeostasis.<br>Induces release of cytochrome c from mitochondria with resultant induction of apoptosis.	dipeptides and potentially plays a significant role in glutathione homeostasis<br>• may play an important role in the induction of apoptosis by GGO in leukemia U937 cells<br>• could play a significant role in regulating the synthesis of glutathione by limiting the availability of -glutamylcysteine					
OPLAH<br>(26873)<br>8q24	5-oxoprolinase (ATP-hydrolysing)	Catalyzes the cleavage of 5-oxo-L-proline to form L-glutamate coupled to the hydrolysis of ATP to ADP and inorganic phosphate. Catalytic Activity 5-oxo-L-proline + ATP + 2 H_2O = ADP + H+ + L-glutamate + phosphate	• catalyzing the cleavage of 5-oxo-L-proline to form L-glutamate coupled to the hydrolysis of ATP to ADP<br>• and inorganic phosphate	++	++	++	++	++
SLC7A11<br>(23657)<br>4q28.3	solute carrier family 7 member 11	Sodium-independent, high-affinity exchange of anionic amino acids with high specificity for anionic form of cystine and glutamate.	• involved in sodium-independent, high-affinity exchange of anionic amino acids with high specificity<br>• for anionic form of cystine and glutamate<br>• major genetic regulator of pheomelanin pigment in hair and melanocytes, with minimal or no effects on eumelanin, and controling the production of pheomelanin pigment<br>• directly<br>• has a critical role in the generation of glutathione and the protection of cells from oxidative stress<br>• promotes cystine uptake and glutathione biosynthesis, resulting in protection from oxidative stress and ferroptotic cell death<br>• also plays critical roles in glutamine metabolism and regulates the glucose and glutamine dependency of cancer cells<br>• ferroptosis is triggered by lipid peroxidation and is tightly regulated by SLC7A11, a key component of the cystine-glutamate antiporter<br>• imports extracellular cystine into cells to promote glutathione synthesis, thus inhibiting ferroptosis	+	++	+	++	++
ABCC1<br>(4363)<br>16p13.11	ATP binding cassette subfamily C member 1	Mediates export of organic anions and drugs from the cytoplasm.<br>Mediates ATP-dependent transport of	• ATP-binding cassette transporter that confers resistance to drugs(colchicine doxorubicine) and mediates the transport of organic anions	+	++	++	++	++

		glutathione and glutathione conjugates, leukotriene C4, estradiol-17-beta-o-glucuronide, methotrexate, antiviral drugs and other xenobiotics.<br>Confers resistance to anticancer drugs by decreasing accumulation of drug in cells, and by mediating ATP- and GSH-dependent drug export.<br>Hydrolyzes ATP with low efficiency.<br>Catalyzes the export of sphingosine 1-phosphate from mast cells independently of their degranulation.<br>Participates in inflammatory response by allowing export of leukotriene C4 from leukotriene C4-synthezing cells. Catalytic Activity ATP + H_2O + xenobiotic Side 1 = ADP + phosphate + xenobiotic Side 2.	• glutathione (GSH) cce protein ()onjugate (GS-x) pump or multispecific anion transporter<br>• having specific biologic functions in epidermal lipid reorganization during terminal keratinocyte differentiation<br>• playing a role in export of sphingosine-1-phosphate from mast cells<br>• may play an important role in smoking-related lung function loss and development of chronic obstructive pulmonary disease (COPD)<br>• with P-glycoprotein (ABCB1) and the breast cancer resistance protein (ABCG2), confers resistance to a large number of structurally diverse drugs<br>• ability to mediate ATP-dependent CBL transport and having a physiologic role in CBL homeostasis<br>• mediates the ATP-dependent efflux of a variety of organic anions derived from both endogenous metabolites and exogenous xenobiotics, many of which are conjugated to glutathione<br>• its activity is not correlated with the level of free cholesterol or with localization in cholesterol-dependent lipid rafts					
NAT8<br>(9027)<br>2p13.1	N-acetyltransferase 8	Acetylates the free alpha-amino group of cysteine S-conjugates to form mercapturic acids. This is the final step in a major route for detoxification of a wide variety of reactive electrophiles which starts with their incorporation into glutathione S-conjugates. The glutathione S-conjugates are then further processed into cysteine S-conjugates and finally mercapturic acids which are water soluble and can be readily excreted in urine or bile. Alternatively, may have a lysine N-acetyltransferase activity catalyzing peptidyl-lysine N6-acetylation of various proteins. Thereby, may regulate apoptosis through the	• involved in development and maintenance of kidney and liver structure and function<br>• putative N-acetyltransferase that produces N-acetylaspartate in brain<br>• involved in mercapturic acid formation<br>• involved in mercapturic acid formation<br>• catalyze transfer of an acetyl group from acetyl-coenzyme A to a wide range of acceptor substrates<br>• NAT8 and NAT8B share enzymatic properties but differ in that NAT8B is post-translationally regulated via acetylation<br>• important regulatory roles for NAT8B and NAT8 in neuron function with induction of NAT8 expression potentially serving as a critical event that adjusts the efficiency of the secretory pathway for activity-dependent neuronal functions	++	++	+	+++	++

		acetylation and the regulation of the expression of PROM1.<br>May also regulate amyloid beta-peptide secretion through acetylation of BACE1 and the regulation of its expression in neurons. Catalytic Activity acetyl-CoA + an S-substituted L-cysteine = an N-acetyl-L-cysteine-S-conjugate + CoA + H+						
GPX1<br>(2876)<br>3p21.31	Glutathione peroxidase 1	Protects the hemoglobin in erythrocytes from oxidative breakdown. In platelets, plays a crucial role of glutathione peroxidase in the arachidonic acid metabolism.<br>Catalytic Activity<br>2 glutathione + H_2O_2 = glutathione disulfide + 2 H_2O<br>This reaction proceeds in the forward direction.	• free radical detoxification<br>• catabolic pathway of activated oxygen species<br>• involved in the protection of the hemoglobin in erythrocytes from oxidative breakdown<br>• implicated as a mechanism of resistance against oxygen free radicals<br>• plays an important role in cellular defense by converting hydrogen peroxide and organic hydroperoxides to nonreactive products<br>• likely a major role for GPX1 in cardiac dysfunction in ANGPT2-dependent hypertension<br>• modulates potentially the endothelial cell response to LPS, in part, by altering CD14-mediated effects<br>• selenium-containing protein, associated with cancer risk and development<br>• regulates leukocyte-endothelial cell interactions in the cerebral microvasculature, but that this is affected by the nature of the inflammatory insult<br>• GPX5 protein, and the epithelial proteins GPX1, GPX3, and cellular GPX4, all functioning in the mammalian epididymis at different stages of the sperm epididymal journey, and in different epididymis compartments<br>• anti-oxidant enzyme that may protect lungs from oxidative damage<br>• primes potentially pro-inflammatory cytokine production after LPS challenge	+	-	++	++	++
GPX2<br>(2877)<br>14q24.1	Glutathione peroxidase 2	Could play a major role in protecting mammals from the toxicity of ingested organic hydroperoxides.	• glutathione peroxidase involved in free radical detoxification<br>• involved in transcriptional response to exogenous	+++	++	+	++	++

		Tert-butyl hydroperoxide, cumene hydroperoxide and linoleic acid hydroperoxide but not phosphatidycholine hydroperoxide, can act as acceptors<br>Catalytic Activity<br>a hydroperoxide + 2 glutathione = an alcohol + glutathione disulfide + H_2O	redox agents and catabolic pathway of activated oxygen species					
GPX3<br>(2878)<br>5q33.1	Glutathione peroxidase 3	Protects cells and enzymes from oxidative damage, by catalyzing the reduction of hydrogen peroxide, lipid peroxides and organic hydroperoxide, by glutathione. Catalytic Activity 2 glutathione + H_2O_2 = glutathione disulfide + 2 H_2O	• involved in free radical detoxification and reduction of hydrogen peroxide<br>• catalyzing the reduction of hydrogen peroxide, organic hydroperoxide, and lipid peroxides by reduced glutathione<br>• functioning in the protection of cells against oxidative damage<br>• having potent extracellular antioxidant activity<br>• major antioxidative enzyme that was induced upon nontoxic proteasome inhibition in endothelial cells<br>• borderline defense mechanism of endothelial cell-derived GPx-3 to protect the vasculature from oxidative stress<br>• GPX5 protein, and the epithelial proteins GPX1, GPX3, and cellular GPX4, all functioning in the mammalian epididymis at different stages of the sperm epididymal journey, and in different epididymis compartments<br>• from the blood binds to basement membranes of specific epithelial cells and the cells modify their basement membranes to cause the binding<br>• importance of this plasma antioxidant enzyme in regulating platelet activity, endothelial function, platelet-dependent thrombosis, and vascular thrombotic propensity<br>• plasma antioxidant enzyme, maintaining genomic integrity by inactivating reactive oxygen species (ROS), known DNA-damaging agents and mediators of cancer chemotherapy response<br>• new retinoid target gene, crucial for human skeletal	++	+++	+++	+++	-

			muscle precursor cell survival<br>• plays a main role in removing hydro- and lipoperoxides from the body<br>• has an important function of scavenging hydrogen peroxide and preventing cancer					
GPX4<br>(2879)<br>19p13.3	Glutathione peroxidase 4	Essential antioxidant peroxidase that directly reduces phospholipid hydroperoxide even if they are incorporated in membranes and lipoproteins (By similarity).<br>Can also reduce fatty acid hydroperoxide, cholesterol hydroperoxide and thymine hydroperoxide (By similarity).<br>Plays a key role in protecting cells from oxidative damage by preventing membrane lipid peroxidation (By similarity).<br>Required to prevent cells from ferroptosis, a non-apoptotic cell death resulting from an iron-dependent accumulation of lipid reactive oxygen species.<br>The presence of selenocysteine (Sec) versus Cys at the active site is essential for life: it provides resistance to overoxidation and prevents cells against ferroptosis (By similarity).<br>The presence of Sec at the active site is also essential for the survival of a specific type of parvalbumin-positive interneurons, thereby preventing against fatal epileptic seizures (By similarity).<br>May be required to protect cells from the toxicity of ingested lipid hydroperoxides (By similarity).<br>Required for normal sperm development and male fertility (By similarity).	• glutathione peroxidase, free radical detoxification, catabolic pathway of activated oxygen species, oxydoreductase<br>• catalyzing the reduction of hydrogen peroxide, organic hydroperoxide, and lipid peroxides by reduced glutathione and functions in the protection of cells against oxidative damage<br>• playing an indispensable role for structural integrity of spermatozoa and to codetermine sperm motility and viability<br>• playing a pivotal role in brain development and function<br>• multifunctional and it acts as a reducing enzyme of peroxidized phospholipids and thiols and as a structural protein<br>• essential antioxidant enzyme having multiple functions<br>• GPX5 protein, and the epithelial proteins GPX1, GPX3, and cellular GPX4, all functioning in the mammalian epididymis at different stages of the sperm epididymal journey, and in different epididymis compartments<br>• nuclear form of GPX4 is associated with sperm nuclear matrix and is required for proper paternal chromatin decondensation at fertilization<br>• GPX4 is a critical antioxidant enzyme for the maturation and survival of photoreceptor cells<br>• is essential for maintaining oxidative homeostasis and keeping defense against oxidative stress in conjunctival epithelial cells<br>• antioxidant defense enzyme active in repairing oxidative damage to lipids, that is a key inhibitor of ferroptosis, a non-apoptotic form of cell death	+	++	++	++	+

		Essential for maturation and survival of photoreceptor cells (By similarity).<br>Plays a role in a primary T-cell response to viral and parasitic infection by protecting T-cells from ferroptosis and by supporting T-cell expansion (By similarity).<br>Plays a role of glutathione peroxidase in platelets in the arachidonic acid metabolism.<br>Reduces hydroperoxy ester lipids formed by a 15-lipoxygenase that may play a role as down-regulator of the cellular 15-lipoxygenase pathway. Catalytic Activity<br>a hydroperoxy polyunsaturated fatty acid + 2 glutathione = a hydroxy polyunsaturated fatty acid + glutathione disulfide + H_2O	involving lipid reactive oxygen species<br>• ferroptosis inhibition by GPX4 is essential for motor neuron health and survival<br>• is an important antioxidant enzyme for maintaining redox status and protecting corneal endothelial cells from oxidative stress<br>• is a monomeric glutathione peroxidase, unique in reducing the hydroperoxide group (-OOH) of fatty acids esterified in membrane phospholipids<br>• paternal acetylated histones and acrosomal nuclear GPX4 are directly involved in fertilization<br>• antioxidant enzyme, having biological important functions such as signaling cell death by suppressing peroxidation of membrane phospholipids<br>• GPX4 regulates reactive oxygen species-induced cell death<br>• ferroptosis is mediated by the accumulation of lipid peroxides upon the depletion/or inhibition of glutathione peroxidase 4 (GPX4)<br>• unique antioxidant enzyme that can directly reduce phospholipid hydroperoxide<br>• increased lipid peroxidation resulting from reduced GPX4 expression levels may be causally associated with lung fibrosis development					
GPX5<br>(2880)<br>6p21.32	Glutathione peroxidase 5	Protects cells and enzymes from oxidative damage, by catalyzing the reduction of hydrogen peroxide, lipid peroxides and organic hydroperoxide, by glutathione. May constitute a glutathione peroxidase-like protective system against peroxide damage in sperm membrane lipids.<br>Catalytic Activity<br>2 glutathione + H_2O_2 = glutathione disulfide + 2 H_2O	• may protect cells and enzymes from oxidative dammage by catalyzing the reduction of hydrogen perodie, lipid peroxides and organic peroxides<br>• may act to protect sperm form premature acrosome reaction in the epididymis<br>• because of the very low level of expression of the active form, these functions remains to be demonstrated<br>• key importance of GPX5 as an extracellular antioxidant designed to protect maturing mammalian spermatozoa from oxidative stress<br>• GPX5 protein, and the epithelial proteins GPX1, GPX3, and cellular GPX4, all functioning in the mammalian epididymis at different stages of the	++	++	+	++	++

			sperm epididymal journey, and in different epididymis compartments					
GPX6 (257202) 6p22.1	Glutathione peroxidase 6	Catalytic Activity<br>2 glutathione + H_2O_2 = glutathione disulfide + 2 H_2O	• protecting cells and enzymes from oxidative damage, by catalyzing the reduction of hydrogen peroxide, lipid peroxides and organic hydroperoxide, by glutathione			No data		
GPX7 (2882) 1p32.3	Glutathione peroxidase 7	It protects esophageal epithelia from hydrogen peroxide-induced oxidative stress. It suppresses acidic bile acid-induced reactive oxigen species (ROS) and protects against oxidative DNA damage and double-strand breaks.<br>Catalytic Activity<br>2 glutathione + H_2O_2 = glutathione disulfide + 2 H_2O	• lutathione peroxidase activity<br>• plays an essential role in breast cancer cells in alleviating oxidative stress generated from polyunsaturated fatty acid metabolism<br>• GPX7 and GPX8 may represent a novel route for the productive use of peroxide produced by ERO1A during disulfide bond formation<br>• is a newly identified stress sensor that transmits oxidative stress signals by forming the disulfide bond between its Cys57 and Cys86 residues<br>• is a monomeric glutathione peroxidase of the endoplasmic reticulum (ER), containing a Cys redox center (CysGPx)<br>• in the ER, the emerging physiological role of GPX7 is oxidation of protein disulfide isomerase (HsPDI), modulated by the amount of GSH (reduced glutathione)<br>• possesses tumour suppressor functions in oesophageal adenocarcinomas (OAC)<br>• protects against fat accumulation via modulating ROS, suggesting the importance of targeting redox homeostasis in obesity management<br>• GPX7 and GPX8 are unique homologs that contain a peroxidatic Cys (CP)<br>• displays a unique function which serves as a stress sensor/transmitter to transfer the signal to its interacting proteins by shuttling disulfide bonds in response to various stresses<br>• role of GPX7 in the maintenance of redox homeostasis<br>• epigenetic silencing of GPX7 could play an important role in gastric tumorigenesis and	++	++	++	++	++

			progression					
CAT<br>(847)<br>11p13	Catalase	Occurs in almost all aerobically respiring organisms and serves to protect cells from the toxic effects of hydrogen peroxide. Promotes growth of cells including T-cells, B-cells, myeloid leukemia cells, melanoma cells, mastocytoma cells and normal and transformed fibroblast cells.<br>Catalytic Activity<br>$2 H_2O_2 = 2 H_2O + O_2$	• involved in catabolic pathway of activated oxygen species and free radical detoxification<br>• catalase overexpression has a protective role against UVB irradiation by preventing DNA damage mediated by the late ROS increase<br>• catalase is an antioxidant enzyme the activity of which is crucial for the protection against damage caused by reactive oxygen species<br>• heme enzymes, which catalyze decomposition of hydrogen peroxide to water and molecular oxygen<br>• is an important antioxidant enzyme that dismutates hydrogen peroxide into water and molecular oxygen<br>• catalase is a key enzyme in the metabolism of H2O2 and reactive nitrogen species, and its expression and localization is markedly altered in tumors<br>• major peroxisome protein, plays a critical role in removing peroxisome-generated reactive oxygen species (ROS) produced by peroxisome enzymes<br>• catalase plays an important role in pexophagy during nutrient deprivation<br>• potential therapeutic effects in nonalcoholic fatty liver disease (NAFLD) progression	++	-	++	+++	+++
SOD1<br>(6647)<br>21q22.11	superoxide dismutase 1	Destroys radicals which are normally produced within the cells and which are toxic to biological systems.	• catabolic pathway of activated oxygen species,free radical detoxification<br>• role in human placenta development (<br>• may play a role in the regulation of cellular lifespan by p53 and may also regulate the death signals in cancer cells (<br>• involved in the function of motor neuron<br>• an important biological role for this enzyme in the preservation of mitochondrial homeostasis (<br>• cuproenzyme that catalyzes the dismutation of the toxic superoxide anion, a byproduct of cellular respiration<br>• functioning as a molecular switch that activates the ER stress response, which plays an important role in cellular homeostasis under zinc-deficient conditions	++	-	+	++	++

SOD2<br>(6648)<br>6q25.3	superoxide dismutase 2	Catalytic Activity<br>2 H+ + 2 superoxide = H_2O_2 + O_2	• manganese superoxide dismutase catabolic pathway of activated oxygen species, free radical detoxification<br>• destroying radical which are normally produced within the cells and which are toxic to biological system<br>• acting as an iron toxicity modifier<br>• function as the first line of antioxidant defense against highly reactive superoxide radicals<br>• with MIF appear to be early predictors for survival in septic patients<br>• binds with ubiquitin molecules to form polyubiquitination chains and undergoes degradation through the ubiquitin-proteasomal pathway<br>• major mitochondrial antioxidative enzyme, constituting an important control switch in the process of activation-induced oxidative signal generation in T cells<br>• critical role for SOD2 and mitochondria in the regulation of human T cell activation<br>• regulates a "metabolic switch" during progression from quiescent through the proliferative cycle<br>• is potentially a new molecular player contributing to the Warburg effect	+++	++	++	++	+++
SOD3<br>(6649)<br>4p15.3-p15.1	superoxide dismutase 3	Protect the extracellular space from toxic effect of reactive oxygen intermediates by converting superoxide radicals into hydrogen peroxide and oxygen.<br>Catalytic Activity<br>2 H+ + 2 superoxide = H_2O_2 + O_2	• antioxidant enzymes that catalyze the dismutation of two superoxide radicals into hydrogen peroxide and oxygen<br>• invoved in catabolic pathway of activated oxygen species, and free radical detoxification<br>• may be protecting the brain, lungs, and other tissues from oxidative stress<br>• tetrameric metalloenzyme responsible for the removal of superoxide anions from the extracellular space	++	++	+++	++	+
PRDX1<br>(5052)<br>1p34.1	peroxiredoxin 1	Thiol-specific peroxidase that catalyzes the reduction of hydrogen peroxide and organic hydroperoxides to water and	• playing a role in elimination of peroxides generated during metabolism<br>• having a function in apoptosis signal-regulating	++	-	++	+	+

	alcohols, respectively. Plays a role in cell protection against oxidative stress by detoxifying peroxides and as sensor of hydrogen peroxide-mediated signaling events. Might participate in the signaling cascades of growth factors and tumor necrosis factor-alpha by regulating the intracellular concentrations of H2O2.<br>Reduces an intramolecular disulfide bond in GDPD5 that gates the ability to GDPD5 to drive postmitotic motor neuron differentiation.	kinase 1 (MAP3K5)-mediated signaling pathway<br>• have a role in both the resistance of certain cancers to therapy and quite a different role in possibly slowing the progression of neurodegenerative<br>• having a function in apoptosis signal-regulating kinase 1 (MAP3K5)-mediated signaling pathway<br>• peroxidase function of PRDX1 is involved in regulating RAS-induced transformation<br>• pivotal regulator of GDPD5 activity, suggesting roles for coupled thiol-redox-dependent cascades in controlling neuronal differentiation in the spinal cord<br>• catalyze peroxide reduction of H(2)O(2), organic hydroperoxides and peroxynitrite<br>• involved in the cellular protection against oxidative stress, the modulation of intracellular signalling cascades as well as the regulation of cell proliferation and apoptosis<br>• essential for preventing respiratory syncytial virus-induced oxidative damage in a subset of nuclear intermediate filament and actin binding proteins in epithelial cells<br>• PRDX1-stimulated endothelial cell proliferation, migration, and differentiation in a TLR4- and VEGF-dependent manner<br>• role in regulating TGFB1-induced epithelial-to-mesenchymal transition (EMT)<br>• detoxifies peroxide substrates and has been implicated in numerous biological processes, including cell growth, proliferation, differentiation, apoptosis, and redox signaling<br>• may perform biological functions as a RNA-binding protein, which are distinctive from known functions of PRDX1 as a reactive oxygen species scavenger<br>• novel role of PRDX1 in phagocytosis of macrophage<br>• orchestrates potentially redox signaling in an H&<br>• 8322; dose-dependent manner through the oxidation status of its peroxidatic cysteine Cys52	

			• is involved in disassembly of flagella and cilia, and suggested that the abnormal expression of the cilia-related gene including PRDX1 may affect both ciliogenesis and cancerogenesis<br>• site-specific acetylation of PRDX1 may change its biological role in response to environmental changes<br>• involved in the control of the pericentrosomal H2O2 level that is critical for mitotic progression<br>• interaction of PRDX1 with APEX1 represents a novel anti-inflammatory function of PRDX1, whereby the association safeguards APEX1 from reducing transcription factors and activating superfluous gene expression, which otherwise could trigger cancer invasion and metastasis					
PRDX2<br>(7001)<br>19p13.2	peroxiredoxin 2	Thiol-specific peroxidase that catalyzes the reduction of hydrogen peroxide and organic hydroperoxides to water and alcohols, respectively. Plays a role in cell protection against oxidative stress by detoxifying peroxides and as sensor of hydrogen peroxide-mediated signaling events. Might participate in the signaling cascades of growth factors and tumor necrosis factor-alpha by regulating the intracellular concentrations of H_2O_2. Catalytic Activity<br>[thioredoxin]-dithiol + a hydroperoxide = [thioredoxin]-disulfide + an alcohol + H_2O	• may be playing an important role in eliminating peroxides generated during metabolism<br>• involved in redox regulation of the cell<br>• reducing peroxides with reducing equivalents provided through the thioredoxin system<br>• might participate in the signaling cascades of growth factors and tumor necrosis factor-alpha by regulating the intracellular concentrations of $H(_2)O(_2)$<br>• enhancing natural killer (NK) cells activity<br>• acting as a negative regulator of PDGF signaling<br>• have a role in both the resistance of certain cancers to therapy and quite a different role in possibly slowing the progression of neurodegenerative diseases<br>• functioning as a noncatalytic scavenger of low-level hydrogen peroxide in the erythrocyte and having nonredundant role in erythrocyte defense against oxidative stress<br>• thiol-specific peroxidase, that regulate proinflammatory responses, vascular remodeling, and global oxidative stress<br>• pecific peroxidase that inhibits atherogenic responses in vascular and inflammatory cells<br>• has been shown to regulate diverse biological functions as cell proliferation and planar cell	++	++	++	++	+

			polarity, and also to affect wound healing<br>• PRDX2 functions are modulated in response to oxidative stress in diseased Red blood cells<br>• protects cells from deleterious oxidative damage<br>• PRDX2 and PRDX4 are negative regulators of hypoxia-inducible factors under conditions of prolonged hypoxia					
PRDX3 (10935) 10q26.11	peroxiredoxin 3	Thiol-specific peroxidase that catalyzes the reduction of hydrogen peroxide and organic hydroperoxides to water and alcohols, respectively. Plays a role in cell protection against oxidative stress by detoxifying peroxides. Acts synergistically with MAP3K13 to regulate the activation of NF-kappa-B in the cytosol.	• may be involved in the regulation of cellular proliferation, differentiation and antioxidant functions<br>• regulating erythroid differentiation (late stage of proerythrocyte differentiation)<br>• critical regulator of the abundance of mitochondrial $H(_2)O(_2)$, which itself promotes apoptosis in cooperation with other mediators of apoptotic signaling<br>• may be important in protecting photoreceptor mitochondria especially in blue cones<br>• PRDX3 and PRDX4, may act as new placental immune targets, and are involved in recurrent pregnancy loss	++	++	+	++	++
PRDX4 (10549) Xp22.11	peroxiredoxin 4	Thiol-specific peroxidase that catalyzes the reduction of hydrogen peroxide and organic hydroperoxides to water and alcohols, respectively. Plays a role in cell protection against oxidative stress by detoxifying peroxides and as sensor of hydrogen peroxide-mediated signaling events. Regulates the activation of NF-kappa-B in the cytosol by a modulation of I-kappa-B-alpha phosphorylation.	• playing a regulatory role in the activation of the transcription factor NF-kappaB<br>• involved in protection against oxidative stress through the detoxification of cellular peroxides<br>• endoplasmic reticulum-localized enzyme forming oligomeric complexes in human cells<br>• important for spermatogenesis, but not an absolute requisite<br>• essential for preventing respiratory syncytial virus-induced oxidative damage in a subset of nuclear intermediate filament and actin binding proteins in epithelial cells<br>• ER-localized and implicated in a previously unanticipated, parallel, ERO1-independent pathway that couples hydroperoxide production to oxidative protein folding in mammalian cells<br>• PRDX3 and PRDX4, may act as new placental	++	-	++	+++	-

			immune targets, and are involved in recurrent pregnancy loss<br>• different binding properties of ERO1A and PRDX4 increase the robustness of ER redox homeostasis<br>• may have a close relationship with follicular development<br>• PRDX4-recycling in the endoplasmic reticulum is much less efficient than in the cytosol or mitochondria, leading to the protection of PRDX4 from hyperoxidation<br>• ER-specific antioxidative peroxidase that can utilize luminal H2O2 as driving force for reoxidizing protein disulfide isomerase family members, thus efficiently contributing to disulfide bond formation<br>• important role of PRDX4 in maintaining insulin levels and improving the ER folding capacity also under conditions of a high insulin requirement<br>• functions not only to eliminate peroxide but also to promote oxidative protein folding via oxidizing protein disulfide isomerase (PDI)<br>• novel function of secreted PRDX4 in mediating osteoclast activation by cancer cells<br>• PRDX4 oxidative activity acts as a sensor to directly couple neuronal differentiation with redox environments in the ER<br>• PRDX2 and PRDX4 are negative regulators of hypoxia-inducible factors under conditions of prolonged hypoxia	
PRDX5 (25824) 11q13.1	peroxiredoxin 5	Thiol-specific peroxidase that catalyzes the reduction of hydrogen peroxide and organic hydroperoxides to water and alcohols, respectively. Plays a role in cell protection against oxidative stress by detoxifying peroxides and as sensor of hydrogen peroxide-mediated signaling events.	• peroxynitrite reductase, playing an antioxidant protective role in various tissues under nonpathologic conditions and during inflammatory processes<br>• reducing hydrogen peroxide and alkyl hydroperoxides with reducing equivalents provided through the thioredoxin system<br>• protecting mitochondrial DNA (mtDNA) from oxidative attacks<br>• may play an important role in mitochondrial genome	No data

			stability<br>• thiol-specific antioxidant protein that is highly expressed in cancer cells<br>• might protect cells from oxidative stress<br>• transcriptional corepressor, as a peroxisomal/mitochondrial antioxidant protein, and as an inhibitor of TP53-dependent apoptosis<br>• constitutively expressed PRDX5 gene plays an important role in protecting the genome against oxidation and may also be involved in the control of transcription of noncoding DNA (Kropotov 2006)					
PRDX6<br>(9588)<br>1q25.1	peroxiredoxin 6	Catalytic Activity<br>[thioredoxin]-dithiol + a hydroperoxide = [thioredoxin]-disulfide + an alcohol + H_2O	• "moonlighting" protein with both GSH peroxidase and phospholipase A2 (PLA2)) activities<br>• involved in redox regulation of the cell<br>• reducing $H(_2)O(_2)$ and short chain organic, fatty acid and phospholipid hydroperoxides<br>• playing a role in the regulation of phospholipid turnover as well as in protection against oxidative injury<br>• regulating, with arachidonic acid, striated muscle growth by modulating multiple growth-regulatory pathways<br>• bifunctional enzyme with two distinct active sites<br>• required for blood vessel integrity in wounded skin<br>• important antioxidant enzyme in human brain defenses<br>• attenuates oxidative stress- and TGFbeta-induced abnormalities of trabecular meshwork cells<br>• binds to reduced phospholipid at acidic pH but at cytosolic pH binds only phospholipid that is oxidized compatible with a role in the repair of peroxidized cell membranes<br>• in uterus PRDX6 also plays antioxidant roles in human implantation	+	++	++	++	++
TXNRD1<br>(7296)<br>12q23.3	thioredoxin reductase 1	Reduces disulfideprotein thioredoxin (Trx) to its dithiol-containing form.<br>Homodimeric flavoprotein involved in the regulation of cellular redox reactions,	• key enzyme in the regulation of the intracellular redox environment<br>• mitochondrial TXNRD3 is much less dependent upon the use of selenocysteine (Sec) for the reduction	++	+	+++	+	++

		growth and differentiation. Contains a selenocysteine residue at the C-terminal active site that is essential for catalysis (Probable). Also has reductase activity on hydrogen peroxide (H_2O_2).<br>Catalytic Activity<br>[thioredoxin]-dithiol + NADP+ = [thioredoxin]-disulfide + H+ + NADPH<br>This reaction proceeds in the backward direction.	of small molecules, as TXNRD1<br>• overexpression of the cytosolic form, TXNRD1, did not produce a lifespan extension in males flie					
TXNRD2 (10587) 22q11.21	thioredoxin reductase 2	Involved in the control of reactive oxygen species levels and the regulation of mitochondrial redox homeostasis.<br>Maintains thioredoxin in a reduced state. May play a role in redox-regulated cell signaling. Catalytic Activity<br>[thioredoxin]-dithiol + NADP+ = [thioredoxin]-disulfide + H+ + NADPH<br>This reaction proceeds in the backward direction.	• thioredoxin reductase activity<br>• participates in the regulation of protein tyrosine phosphorylation and cell growth<br>• play essential roles in cell survival and mitochondria-mediated apoptosis<br>• overexpression of mitochondrial TXNRD2 in Drosophila melanogaster extended median (but not maximum) lifespan in female flies with a small lifespan extension in males	+	++	++	++	+
TXNRD3 (114112) 3q21.3	thioredoxin reductase 3	Displays thioredoxin reductase, glutaredoxin and glutathione reductase activities. Catalyzes disulfide bond isomerization. Promotes disulfide bond formation between GPX4 and various sperm proteins and may play a role in sperm maturation by promoting formation of sperm structural components.<br>Catalytic Activity [thioredoxin]-dithiol + NADP+ = [thioredoxin]-disulfide + H+ + NADPH	• acting on NADH or NADPH as acceptor<br>• may have an essential role in redox-regulated cell signaling<br>• mitochondrial TXNRD3 is much less dependent upon the use of selenocysteine (Sec) for the reduction of small molecules, as TXNRD1<br>• plays a critical role in male reproduction via the thiol redox control of spermatogenesis	++	++	+	++	++
MSRA (4482) 8p23.1	methionine sulfoxide reductase A	Has an important function as a repair enzyme for proteins that have been inactivated by oxidation. Catalyzes the reversible oxidation-reduction of methionine sulfoxide in proteins to methionine. Catalytic Activity	• having an oxidoreductase activity, and reducing methionine S-sulfoxide<br>• antioxidant repair enzyme that reduces oxidized methionine to methionine<br>• might possess metastasis suppressor activity in hepatocellular carcinoma	+	++	++	+++	++

		[thioredoxin]-disulfide + H_2O + L-methionyl-[protein] = [thioredoxin]-dithiol + L-methionyl-(S)-S-oxide-[protein]	• may play an important role in protecting macular RPE from oxidative damage<br>• capable of protecting cells against oxidative damage by reversing damage to proteins caused by methionine oxidation or by decreasing ROS through a scavenger mechanism<br>• protects cardiac myocytes against hypoxia/reoxygenation induced cell death<br>• required for maintaining the reduced state of alpha-crystallin methionines in the lens<br>• essential enzyme in the antioxidant system, which scavenges reactive oxygen species through cyclic oxidation and reduction of methionine and methionine sulfoxide<br>• enzyme that reverses oxidation of methionine in proteins<br>• required when cells are stressed may be related to the scavenger role of the MSR. system, and under stress conditions required to provide maximal protection<br>• required for lens cell maintenance, defense against oxidative stress damage, mitochondrial function and prevention of lens cataract formation<br>• uses multiple reducing systems for its repair activity that may augment its function under different cellular conditions					
MSRB1 (51734) 16p13.3	methionine sulfoxide reductase B1	Methionine-sulfoxide reductase that specifically reduces methionine (R)-sulfoxide back to methionine. While in many cases, methionine oxidation is the result of random oxidation following oxidative stress, methionine oxidation is also a post-translational modification that takes place on specific residue. Acts as a regulator of actin assembly by reducing methionine (R)-sulfoxide mediated by MICALs (MICAL1, MICAL2 or MICAL3) on actin, thereby promoting filament	• with MSRB3, and MSRB2, required for lens cell viability, and their silencing in lens cells results in increased oxidative-stress-induced cell death<br>• important as antioxidant/repair systems for neutrophils, cells with enormous capacity for the generation of reactive oxidants and hence, susceptible to oxidative damage<br>• repair enzymes that protect proteins from oxidative stress by catalyzing stereospecific reduction of oxidized methionine residues<br>• recovers TRPM6 channel activity by reducing the oxidation of Met(1755) and could, thereby, function	++	++	++	+++	+++

		repolymerization. Plays a role in innate immunity by reducing oxidized actin, leading to actin repolymerization in macrophages.<br>Catalytic Activity<br>[thioredoxin]-disulfide + H2O + L-methionyl-[protein] = [thioredoxin]-dithiol + L-methionyl-(R)-S-oxide-[protein]By Similarity	as a modulator of TRPM6 during oxidative stress					
MSRB2 (22921) 10p12.2	methionine sulfoxide reductase B2	Methionine-sulfoxide reductase that specifically reduces methionine (R)-sulfoxide back to methionine. While in many cases, methionine oxidation is the result of random oxidation following oxidative stress, methionine oxidation is also a post-translational modification that takes place on specific residue. Upon oxidative stress, may play a role in the preservation of mitochondrial integrity by decreasing the intracellular reactive oxygen species build-up through its scavenging role, hence contributing to cell survival and protein maintenance.<br>Catalytic Activity<br>[thioredoxin]-disulfide + H_2O + L-methionyl-[protein] = [thioredoxin]-dithiol + L-methionyl-(R)-S-oxide-[protein]By Similarity	• specifically reducing methionine-R-sulfoxide<br>• with SEPX1, and MSRB3, required for lens cell viability, and their silencing in lens cells results in increased oxidative-stress-induced cell death<br>• important as antioxidant/repair systems for neutrophils, cells with enormous capacity for the generation of reactive oxidants and hence, susceptible to oxidative damage<br>• repair enzymes that protect proteins from oxidative stress by catalyzing stereospecific reduction of oxidized methionine residues	+	++	++	+++	++
MSRB3 (253827) 12q14.3	methionine sulfoxide reductase B3	Catalyzes the reduction of free and protein-bound methionine sulfoxide to methionine. Isoform 2 is essential for hearing.<br>Catalytic Activity<br>[thioredoxin]-disulfide + H_2O + L-methionyl-[protein] = [thioredoxin]-dithiol + L-methionyl-(R)-S-oxide-[protein]	• catalyzes the reduction of free and protein-bound methionine sulfoxide to methionine<br>• with SEPX1, and MSRB2, required for lens cell viability, and their silencing in lens cells results in increased oxidative-stress-induced cell death<br>• important as antioxidant/repair systems for neutrophils, cells with enormous capacity for the generation of reactive oxidants and hence, susceptible to oxidative damage<br>• repair enzymes that protect proteins from oxidative	++	++	++	+	++

			• stress by catalyzing stereospecific reduction of oxidized methionine residues<br>• MSRB3-catalyzed reduction of methionine sulfoxides to methionine is essential for hearing					

[1] Human Gene Nomenclature Committee (https://www.genenames.org); [2] Entrez Gene (https://www.ncbi.nlm.nih.gov/gene) ; [3] UniProt (https://www.uniprot.org) ;
[4] GENATLAS (http://genatlas.medecine.univ-paris5.fr/); [5] BioGPS (http://biogps.org). The number of the "+" symbols means the level of gene expression in the tissues/organs playing a role in the pathogenesis of type 2 diabetes: low (+), medium (++) and high (+++). "-" depicts that the gene expression is too low or not detected (<0.5 Me).
Low expression: >0.5 Me, <1 Me; medium expression: >1 Me, <10 Me; high expression: >10 Me.

Biological functions and tissue-specific gene expression of oxidative enzymes

Approved gene symbol[1] (Gene ID)[2] Chromosomal location[2]	Approved gene name[1] (protein name)[3]	Enzyme/protein function[3]	Basic function[4]	Tissue gene expression[5]				
				Pancreas	Skeletal muscle	Adipose tissue	Liver	Whole blood
LPO (4025) 17q23.1	lactoperoxidase	Heme-containing oxidoreductase which catalyzes the conversion of thiocyanate (SCN-) into antimicrobial agent hypothiocyanous acid (OSCN-) in the presence of hydrogen peroxide (H_2O_2).<br>Also involved in the conversion of iodide (I-) into hypoiodite (IO-) in the presence of H_2O_2.<br>Responsible for the inactivation of a wide range of micro-organisms and hence, important component of defense mechanism.<br>Shows antibacterial properties against Pseudomonas aeruginosa.<br>The lactoperoxidase-SCN- H_2O_2 system shows antibacterial properties against Burkholderia cepacia and Haemophilus influenzae in vitro.<br>Present in mammary and salivary gland secretions and may contribute to airway host defense against infection.<br>May contribute to maintaining an appropriate H_2O_2 cellular level, therefore protecting cells from H_2O_2-caused injuries and inflammation.	• having antimicrobial activity and presumably contributing to the protective functions of milk	++	++	+	++	++

DHCR24 (1718) 1p32.3	24-dehydrocholesterol reductase	Catalyzes the reduction of the delta-24 double bond of sterol intermediates during cholesterol biosynthesis. In addition to its cholesterol-synthesizing activity, can protect cells from oxidative stress by reducing caspase 3 activity during apoptosis induced by oxidative stress. Also protects against amyloid-beta peptide-induced apoptosis. Catalytic Activity cholesterol + NADP+ = desmosterol + H+ + NADPH	• catalyzing the reduction of the delta-24 double bond of sterol intermediates during cholesterol biosynthesis<br>• acting as an antiapoptotic factor in neurons<br>• playing a role in protection of cells against amyloid beta peptide toxicity and oxidative stress<br>• might be involved in the molecular events of adrenocortical tumorigenesis by facilitating steroid synthesis and cell growth<br>• being a key mediator of Ras-induced senescence<br>• regulates responses to oncogenic and oxidative stimuli<br>• flavin adenine dinucleotide-dependent oxidoreductase regulating responses to oncogenic and oxidative stimuli<br>• playing a role in cholesterol biosynthesis, APP processing and Abeta generation<br>• exerts an anti-apoptotic function as a reactive oxygen species (ROS) scavenger, for which it needs its FAD-binding domain<br>• anti-apoptotic function of DHCR24 is likely associated with its cleavage by caspase<br>• involved in cell growth, senescence and cellular response to oncogenic and oxidative stress<br>• endoplasmic reticulum (ER)-localized multifunctional enzyme that possesses anti-apoptotic and cholesterol-synthesizing activitie<br>• could protect neuronal cells from apoptosis induced by ER stress<br>• DHCR24 associates strongly with the endoplasmic reticulum beyond predicted membrane domains: implications for the activities of this multi-functional enzyme<br>• converts desmosterol into cholesterol	+	+	+++	+++	+

SIRT2<br>(22933)<br>19q13.2	sirtuin 2	NAD-dependent protein deacetylase, which deacetylates internal lysines on histone and alpha-tubulin as well as many other proteins such as key transcription factors.<br>Participates in the modulation of multiple and diverse biological processes such as cell cycle control, genomic integrity, microtubule dynamics, cell differentiation, metabolic networks, and autophagy.<br>Plays a major role in the control of cell cycle progression and genomic stability.<br>Functions in the antephase checkpoint preventing precocious mitotic entry in response to microtubule stress agents, and hence allowing proper inheritance of chromosomes.<br>Positively regulates the anaphase promoting complex/cyclosome (APC/C) ubiquitin ligase complex activity by deacetylating CDC20 and FZR1, then allowing progression through mitosis.<br>Associates both with chromatin at transcriptional start sites (TSSs) and enhancers of active genes.<br>Plays a role in cell cycle and chromatin compaction through epigenetic modulation of the regulation of histone H4 'Lys-20' methylation (H4K20me1) during early mitosis.<br>Specifically deacetylates histone H4 at 'Lys-16' (H4K16ac) between the G2/M transition and metaphase enabling H4K20me1 deposition by KMT5A leading to ulterior levels of H4K20me2 and H4K20me3 deposition throughout cell cycle, and mitotic S-phase progression.<br>Deacetylates KMT5A modulating KMT5A chromatin localization during the mitotic stress response.	• conserved NAD+-dependent deacetylases and ADP-ribosyltransferases involved in the regulation of cell division, apoptosis, DNA damage repair, genomic silencing, and longevity<br>• deacetylating the Lys-40 of alpha-tubulin (role in the cell cycle regulation)<br>• involved in the control of mitotic exit in the cell cycle, probably via its role in the regulation of cytoskeleton<br>• involved in chromatin structure<br>• involved in control of cellular life span<br>• involved in mitotic progression in the normal cell cycle<br>• mitotic checkpoint protein that functions in the early metaphase to prevent chromosomal instability<br>• acts as an important regulator of adipocyte differentiation through modulation of FOXO1 acetylation/phosphorylation and activity and may play a role in controlling adipose tissue mass and function<br>• responds to nutrient deprivation and energy expenditure to maintain energy homeostasis by promoting lipolysis and inhibiting adipocyte differentiation<br>• deacetylase for tubulin and histone H4<br>• function of SIRT2 for centrosome maintenance upon exposure to mitotic stress caused by microtubule inhibitors, but also the existence of a centrosome-mediated signaling pathway to sustain the spindle checkpoint<br>• by participating in the stress response to genomic insults, sirtuins are thought to protect against cancer, but they are also emerging as direct participants in the growth of some cancers<br>• tubulin deacetylase and an important	+	++	+	++	+

	Deacetylates also histone H3 at 'Lys-57' (H3K56ac) during the mitotic G2/M transition.<br>Upon bacterium Listeria monocytogenes infection, deacetylates 'Lys-18' of histone H3 in a receptor tyrosine kinase MET- and PI3K/Akt-dependent manner, thereby inhibiting transcriptional activity and promoting late stages of listeria infection.<br>During oocyte meiosis progression, may deacetylate histone H4 at 'Lys-16' (H4K16ac) and alpha-tubulin, regulating spindle assembly and chromosome alignment by influencing microtubule dynamics and kinetochore function.<br>Deacetylates histone H4 at 'Lys-16' (H4K16ac) at the *VEGFA* promoter and thereby contributes to regulate expression of VEGFA, a key regulator of angiogenesis.<br>Deacetylates alpha-tubulin at 'Lys-40' and hence controls neuronal motility, oligodendroglial cell arbor projection processes and proliferation of non-neuronal cells.<br>Phosphorylation at Ser-368 by a G1/S-specific cyclin E-CDK2 complex inactivates SIRT2-mediated alpha-tubulin deacetylation, negatively regulating cell adhesion, cell migration and neurite outgrowth during neuronal differentiation.<br>Deacetylates PARD3 and participates in the regulation of Schwann cell peripheral myelination formation during early postnatal development and during postinjury remyelination.<br>Involved in several cellular metabolic pathways.<br>Plays a role in the regulation of blood glucose homeostasis by deacetylating and stabilizing	regulator of cell division and myelinogenesis<br>• having an unique role in the control of neuronal metabolism<br>• in non-neuronal cells, function as a tubulin deacetylase and a key regulator of cell division and differentiation<br>• role in regulating microtubule acetylation patterns in neurons, suggesting a novel mechanism by which neuronal function might become impaired in the aging brain<br>• NAD-dependent deacetylase that is an important regulator of programmed necrosis and inhibitors of this deacetylase may constitute a novel approach to protect against necrotic injuries, including ischaemic stroke and myocardial infarction<br>• regulation of protein acetylation by SIRT2 plays a central role in platelet function<br>• SIRT1 and SIRT2 are NAD(+)-dependent histone deacetylases that operate as post-translational regulators for the deacetylation of acetyllysine<br>• function of SIRT2 extends beyond the regulation of microtubules to include the regulation of nuclear envelope dynamics<br>• cytoplasmic sirtuin that plays a role in various cellular processes, including tumorigenesis, metabolism, and inflammation<br>• link between SIRT2 and physiological aging impacting the axonal compartment of the central nervous system, while supporting a major role for SIRT2 in orchestrating its metabolic regulation<br>• inhibits cell motility by suppressing actin polymerization<br>• SIRT2 plays a critical role in mediating the radiation-induced DNA damage response, thus regulating radiation-induced cell death

	phosphoenolpyruvate carboxykinase PCK1 activity in response to low nutrient availability.<br>Acts as a key regulator in the pentose phosphate pathway (PPP) by deacetylating and activating the glucose-6-phosphate G6PD enzyme, and therefore, stimulates the production of cytosolic NADPH to counteract oxidative damage.<br>Maintains energy homeostasis in response to nutrient deprivation as well as energy expenditure by inhibiting adipogenesis and promoting lipolysis.<br>Attenuates adipocyte differentiation by deacetylating and promoting FOXO1 interaction to PPARG and subsequent repression of PPARG-dependent transcriptional activity.<br>Plays a role in the regulation of lysosome-mediated degradation of protein aggregates by autophagy in neuronal cells.<br>Deacetylates FOXO1 in response to oxidative stress or serum deprivation, thereby negatively regulating FOXO1-mediated autophagy.<br>Deacetylates a broad range of transcription factors and co-regulators regulating target gene expression. Deacetylates transcriptional factor FOXO3 stimulating the ubiquitin ligase SCF(SKP2)-mediated FOXO3 ubiquitination and degradation (By similarity).<br>Deacetylates HIF1A and therefore promotes HIF1A degradation and inhibition of HIF1A transcriptional activity in tumor cells in response to hypoxia.<br>Deacetylates RELA in the cytoplasm inhibiting NF-kappaB-dependent transcription activation upon TNF-alpha stimulation.	and survival<br>• plays an important role in the response to stress, thereby modulating depression-like behaviors<br>• NAD-dependent sirtuin deacetylase that regulates microtubule and chromatin dynamics, gene expression, cell cycle progression as well as nuclear envelope reassembly<br>• SIRT2 may also play a role in Golgi structure formation

		Inhibits transcriptional activation by deacetylating p53/TP53 and EP300.<br>Deacetylates also EIF5A.<br>Functions as a negative regulator on oxidative stress-tolerance in response to anoxia-reoxygenation conditions.<br>Plays a role as tumor suppressor.<br>In addition to protein deacetylase activity, also has activity toward long-chain fatty acyl groups and mediates protein-lysine demyristoylation and depalmitoylation of target proteins, such as ARF6 and KRAS, thereby regulating their association with membranes. Catalytic Activity<br>H_2O_2 + N6-acetyl-L-lysyl-[protein] + NAD+ = 2''-O-acetyl-ADP-D-ribose + L-lysyl-[protein] + nicotinamide						
SCARA3<br>(51435)<br>8p21.1	scavenger receptor class A member 3	Seems to protect cells by scavenging oxidative molecules or harmful products of oxidation.	• may be a potent tumor sup-pressor gene<br>• undergoes hypermethylation in over 30 p100 of prostate cancers<br>• probably has a role beyond a cell stress-response gene<br>• induce cell death through a novel mechanism by hijacking a critical RNA processing enzyme	++	++	++	++	+
SRXN1<br>(140809)<br>20p13	sulfiredoxin 1	Contributes to oxidative stress resistance by reducing cysteine-sulfinic acid formed under exposure to oxidants in the peroxiredoxins PRDX1, PRDX2, PRDX3 and PRDX4.<br>Does not act on PRDX5 or PRDX6.<br>May catalyze the reduction in a multi-step process by acting both as a specific phosphotransferase and a thioltransferase.	• catalyzes a novel enzymatic reaction, the reduction of protein cysteine sulfinic acid, Cys-SO(2)(-)<br>• deglutathionylation function of sulfiredoxin suggesting a central role in redox control with potential implications in cell signaling<br>• role in the feedback regulation of AP1 activity	+	++	++	+	++
RAC1<br>(5879)<br>7p22.1	Rac family small GTPase 1	Plasma membrane-associated small GTPase which cycles between active GTP-bound and inactive GDP-bound states. In its active state, binds to a variety of effector proteins to regulate cellular responses such as secretory processes, phagocytosis of apoptotic cells,	• playing a significant role in the bone resorptive activity of cells, probably by regulating the motility of osteoclasts<br>• has a known role in cerebellar development<br>• potent activator of mineralocorticoid receptor signal transduction	++	+	+	+	++

	epithelial cell polarization, neurons adhesion, migration and differentiation, and growth-factor induced formation of membrane ruffles.<br>Rac1 p21/rho GDI heterodimer is the active component of the cytosolic factor sigma 1, which is involved in stimulation of the NADPH oxidase activity in macrophages. Essential for the SPATA13-mediated regulation of cell migration and adhesion assembly and disassembly. Stimulates PKN2 kinase activity.<br>In concert with RAB7A, plays a role in regulating the formation of RBs (ruffled borders) in osteoclasts.<br>In podocytes, promotes nuclear shuttling of NR3C2; this modulation is required for a proper kidney functioning. Required for atypical chemokine receptor ACKR2-induced LIMK1-PAK1-dependent phosphorylation of cofilin (CFL1) and for up-regulation of ACKR2 from endosomal compartment to cell membrane, increasing its efficiency in chemokine uptake and degradation. In neurons, is involved in dendritic spine formation and synaptic plasticity.<br>In hippocampal neurons, involved in spine morphogenesis and synapse formation, through local activation at synapses by guanine nucleotide exchange factors (GEFs), such as ARHGEF6/ARHGEF7/PIX.<br>In synapses, seems to mediate the regulation of F-actin cluster formation performed by SHANK3. In neurons, plays a crucial role in regulating GABA(A) receptor synaptic stability and hence GABAergic inhibitory synaptic transmission through its role in PAK1 activation and eventually F-actin stabilization.	• involved in the production of superoxide in neutrophils<br>• putative regulator of mitogen-induced cytoskeletal changes essential for membrane ruffling and C-jun aminoterminal kinase JNK<br>• regulator of actin filament at the plasma membrane to produce lamellipodia and membrane ruffles, controlling proliferation, adhesion, migration during embryonic development<br>• key downstream target in Ras signaling<br>• linking growth factor receptor to the activator of actin polymerization<br>• activating JNK signaling pathway<br>• reduction of oxygen to O-2 at the expense of NADPH, the O2-generated is the precursor of potent oxidants used to kill the invading microorganisms<br>• exerting its effects in the epidermis by negatively regulating c-Myc through p21-activated kinase 2 (PAK2) phosphorylation<br>• may playing an important role in corneal wound healing<br>• have distinct roles in regulating cell morphology, migration and invasion, but are not essential for macrophage migration or chemotaxis<br>• critically involved in M-CSF receptor signaling and mediates survival signaling primarily through PI3K/Akt pathways<br>• regulating cell adhesion, migration and differentiation in various cell types<br>• role in hematopoietic development and function<br>• critical role in B cell development and signaling<br>• controles the formation of dendrites in mature dendritic cells, their polarized short-range	

migration toward T cells, and T cell priming

- critical for the hypertrophic response in the heart
- negatively controls LPS-induced IL-23 p19 expression through an NF-kappaB p65 trans activation-dependent
- mediator of colony-stimulating factor 1 (CSF-1)-dependent actin remodeling in osteoclasts
- central role for Rac1 in the control of NDRG1-induced breast cancer cell-cycle progression and proliferation through up-regulating the expression of cyclin D1 and CDKN1A
- essential player for mediating the induction of cyclin D1 and CDKN1A by HRG in breast cancer cells
- involved in endothelial cell cytoskeletal reorganization and in neovessel formation
- shares with RAC3 the ability to interfere with cadherin-mediated adhesion
- both RAC1 and RAC3 are important for the development of the nervous system, wherein they play complementary roles during late stages of neuronal and brain development
- its overexpression contributes to the accelerated migration and high proliferation potential of leukemia cells, which could be implicated in leukemia development and progression
- with RHOA, and RHOB, are involved in the establishment of the migratory and invasive phenotype of tumour cells that have CDH1 mutation
- play essential roles in coordinating directional migration and superoxide production during neutrophil responses to chemoattractants
- RAC1 and RAC2 GTPases are essential for normal bone marrow erythropoiesis but that they are dispensable for erythropoiesis in the

			spleen, implying different signaling pathways for homeostatic and stress erythropoiesis<br>• critical regulator of both MTOR and CRTC2 in response to growth-factor stimulation<br>• role for neuronal RAC1 and RAC3 in dictating proper lymphoid organ development, suggesting the existence of lymphoid-extrinsic mechanisms linking neural defects to the loss of immune-competence<br>• RAC1 and RAC3 GTPases participate in the normal development of hilar mossy cells, and indicate that they are involved in the regulation of the migration of the mossy cell precursor by preventing their arrival to the dorsal hilus<br>• cell-autonomous and stage-specific functions for the small Rho GTPases CDC42 and RAC1 in the course of adult hippocampal neurogenesis<br>• RAC1 and CDC42 are molecular switches that control many cellular processes, but are best known for their roles in the regulation of actin cytoskeleton dynamics<br>• divergent roles of RAC1 and CDC42 function in podocyte maintenance and injury<br>• FHOD1 and the small GTPase RAC1 promote vaccinia virus actin-based motility<br>• RAC1 has been reported to function downstream of ARF6 to control membrane ruffling and cell migration<br>• RAC1 controls surface mobilization of CD40LG on activated platelets and MMP9 secretion from neutrophils<br>• RHOA and RAC1 function as master regulators of cytokinesis by controlling the actomyosin cytoskeleton<br>• importance of RAC1 in neuronal development					
RAC2	Rac family small	Plasma membrane-associated small GTPase	• involved in the production of superoxide in	++	+	++	++	+++

(5880)<br>22q13.1	GTPase 2	which cycles between an active GTP-bound and inactive GDP-bound state. In active state binds to a variety of effector proteins to regulate cellular responses, such as secretory processes, phagocytose of apoptotic cells and epithelial cell polarization. Augments the production of reactive oxygen species (ROS) by NADPH oxidase.	neutrophils<br>• putative regulator of mitogen-induced cytoskeletal changes essential for membrane ruffling and C-jun aminoterminal kinase JNK<br>• regulator of actin filament at the plasma membrane to produce lamellipodia and membrane ruffles<br>• key downstream target in Ras signaling<br>• linking growth factor receptor to the activator of actin polymerization<br>• activating JNK signaling pathway<br>• reduction of oxygen to O^{-}_{2} at the expense of NADPH,the O_2-generated is the precursor of potent oxidants used to kill the invading microorganisms<br>• have distinct roles in regulating cell morphology, migration and invasion, but are not essential for macrophage migration or chemotaxis<br>• plays a critical role in host defense<br>• plays an important role in cell migration with activation of membrane ruffling, whereas the other members of the Rho family, like RHOA and CDC42, mediate stress fibers and filopodia formation, respectively<br>• in hematopoietic cells regulates leukocyte lineage distribution and in nonhematopoietic cells might contribute to regulating circulating neutrophil counts<br>• play essential roles in coordinating directional migration and superoxide production during neutrophil responses to chemoattractants<br>• RAC1 and RAC2 GTPases are essential for normal bone marrow erythropoiesis but that they are dispensable for erythropoiesis in the spleen, implying different signaling pathways for homeostatic and stress erythropoiesis<br>• important role in mature osteoclasts

			• required for the formation of neutrophil extracellular traps<br>• NCF1 and RAC2 accumulate only transiently at the phagosome at the onset of NADPH activity and detach from the phagosome before the end of ROS production<br>• controls tumor growth, metastasis and M1-M2 macrophage differentiation<br>• important role for RAC2 in maintaining mitochondrial integrity					
CYBA (1535) 16q24.3	cytochrome b-245 alpha chain	Critical component of the membrane-bound oxidase of phagocytes that generates superoxide. Associates with NOX3 to form a functional NADPH oxidase constitutively generating superoxide.	• oxygen dependent mechanism of phagocytosis<br>• reduction of oxygen to O_2^- at the expense of NADPH, the O_2-generated is the precursor of potent oxidants used to kill the invading microorganisms<br>• encoding the small sub-unit, p22-phox protein, of the cytochrome b 558 of the NADPH oxidase that plays a crucial role in NADPH-dependent reactive oxygen species production	++	-	+	+++	++
CYBB (1536) Xp21.1	cytochrome b-245 beta chain	Critical component of the membrane-bound oxidase of phagocytes that generates superoxide. It is the terminal component of a respiratory chain that transfers single electrons from cytoplasmic NADPH across the plasma membrane to molecular oxygen on the exterior. Also functions as a voltage-gated proton channel that mediates the H+ currents of resting phagocytes. It participates in the regulation of cellular pH and is blocked by zinc.	• oxygen dependent mechanism of phagocytosis<br>• reduction of oxygen to O_2^- at the expense of NADPH, the O_2-generated is the precursor of potent oxidants used to kill the invading microorganisms<br>• critical for the bactericidal activity of phagocytic cells and plays a major role in innate immunity<br>• its activity participates in the regulation of the phagosomal and endosomal pH in dendritic cells, and is required for efficient antigen cross-presentation<br>• with NOX4 mediate proliferative response in endothelial cells<br>• plays a fundamental role in host defense and innate immunity	++	++	++	++	+++

- plays a fundamental role in conferring macrophages with the ability to respond to extracellular ATP stimulation with robust changes in cellular oxidation
- plays an important role in the control of afferent arteriole tone and is involved in the contractile responses to ANGPT2 and/or adenosine
- plays a key role in apoptosis and phagocytosis of hepatocytes inducing activation of hepatic stellate cells and initiation of fibrosis in liver
- role in the activation of autophagy, a cellular degradative pathway
- mediating proliferative response in endothelial cells
- reactive oxygen species generated by NADPH oxidase 2 and 4 (CYBB and NOX4)are required for chondrogenic differentiation
- implicated as a source of rapid stretch-dependent ROS production at the junctional sarcoplasmic reticulum of cardiomyocytes (X-ROS signaling)
- plays a role in neuronal cell death during retinal ischemia
- is a key mediator of insulin resistance in skeletal muscle
- from both hematopoietic and endothelial cells CYBB is crucial for neutrophil-platelet interactions during TNF-induced venular inflammation
- is critical for heterotypic neutrophil-platelet interactions during vascular inflammation
- NOX1 and CYBB play differential roles in different platelet activation pathways and in thrombosis
- is a critical component of the suppressive machinery of CD8 Tregs, suggesting that repairing CYBB deficiency in these cells may

			protect older individuals from tissue-destructive inflammatory disease, such as large-vessel vasculitis<br>• DUOX1 and CYBB play Distinct roles in Redox regulation of epidermal growth factor receptor signaling<br>• distinct cell-specific roles for CYBB in blood pressure (BP) regulation<br>• CYBB- signaling in macrophages participates in the pathogenesis of obesity, and reinforce a key role for macrophage inflammation in diet-induced metabolic and neurologic decline					
NCF1 (653361) 7q11.23	neutrophil cytosolic factor 1	NCF2, NCF1, and a membrane bound cytochrome b558 are required for activation of the latent NADPH oxidase (necessary for superoxide production).	• p47-phox (NCF1), oxygen dependent mechanism of phagocytosis<br>• reduction of oxygen to O-2 at the expense of NADPH, the O2-generated is the precursor of potent oxidant used to kill the invading microorganisms (electron transfer from NADPH to molecular oxygen)<br>• mediating formation of reactive oxygen intermediates (ROI) which play an important role in host defense and autoimmunity<br>• novel role of NCF1 subunit beyond and independent of nicotinamide adenine dinucleotide phosphate oxidase activity as a regulator of cortactin and adaptive cytoskeletal remodeling, leading to a paradoxically enhanced susceptibility to biomechanical stress and heart failure<br>• NCF1 and RAC2 accumulate only transiently at the phagosome at the onset of NADPH activity and detach from the phagosome before the end of ROS production<br>• is a key regulatory subunit of NADPH oxidase	++	++	++	++	+++
NCF2 (4688) 1q25.3	neutrophil cytosolic factor 2	NCF2, NCF1, and a membrane bound cytochrome b558 are required for activation of the latent NADPH oxidase (necessary for superoxide production).	• p67-phox, oxygen dependent mechanism of phagocytosis<br>• reduction of oxygen to O-2 at the expense of NADPH, the O2-generated is the precursor of	++	++	++	++	+++

			potent oxidants used to kill the invading microorganisms<br>• being a membrane bound cytochrome b558 required for activation of the latent NADPH oxidase<br>• essential component of the multi-protein NADPH oxidase enzyme in phagocytic leukocytes, as well as in certain non-phagocytic cells					
NCF4<br>(4689)<br>22q13.1	neutrophil cytosolic factor 4	Component of the NADPH-oxidase, a multicomponent enzyme system responsible for the oxidative burst in which electrons are transported from NADPH to molecular oxygen, generating reactive oxidant intermediates. It may be important for the assembly and/or activation of the NADPH-oxidase complex.	• activating the NADPH oxidase during FcgammaIIA receptor-induced phagocytosis<br>• reduction of oxygen to O^{-}_{2} at the expense of NADPH, the O_2-generated is the precursor of potent oxidants used to kill the invading microorganisms<br>• negatively regulating the NADPH oxidase upon phosphorylation	++	++	+	++	+++
NOXA1<br>(10811)<br>9q34.3	NADPH oxidase activator 1	Functions as an activator of NOX1, a superoxide-producing NADPH oxidase. Functions in the production of reactive oxygen species (ROS) which participate in a variety of biological processes including host defense, hormone biosynthesis, oxygen sensing and signal transduction. May also activate CYBB/gp91phox and NOX3.	• participating in activation of superoxide-producing NADPH oxidases<br>• mediating the binding of other regulatory proteins during activation of the phagocyte oxidase, and its translocation to the membrane is triggered upon cell activation by hyperphosphorylation<br>• acts in conjunction with Nox organizer 1 (NOXO1) to regulate reactive oxygen species (ROS) production by the NADPH oxidase NOX1<br>• likely a key player that links oxidized low-density lipoprotein with the activation of endothelial NAD(P)H oxidase<br>• with NOXO1, having a role, as well as the binding of Rac1 GTPase, for NOX1 activity<br>• functional homolog of NCF2 in VSMCs (vascular smooth muscle cell) that regulates redox signaling and VSMC phenotype			No data		
NOXO1<br>(124056)	NADPH oxidase organizer 1	Constitutively potentiates the superoxide-generating activity of NOX1 and NOX3 and is	• involved in intracellular signaling cascade activating NOX1			No data		

16p13.3		required for the biogenesis of otoconia/otolith, which are crystalline structures of the inner ear involved in the perception of gravity. Isoform 3 is more potent than isoform 1 in activating NOX3. Together with NOXA1, may also substitute to NCF1/p47phox and NCF2/p67phox in supporting the phagocyte NOX2/gp91phox superoxide-generating activity.	• binding to membrane lipids through its PX domain<br>• with NOXA1, having a role, as well as the binding of Rac1 GTPase, for NOX1 activity					
NOX1 (27035) Xq22.1	NADPH oxidase 1	NOH-1S is a voltage-gated proton channel that mediates the H+ currents of resting phagocytes and other tissues. It participates in the regulation of cellular pH and is blocked by zinc. NOH-1L is a pyridine nucleotide-dependent oxidoreductase that generates superoxide and might conduct H+ ions as part of its electron transport mechanism, whereas NOH-1S does not contain an electron transport chain.	• involved in growth control in non phagocytic cells<br>• pyridine nucleotide dependent superoxide generating<br>• acting as a voltage gated proton (H+) channel<br>• playing a crucial role in irradiation-induced ROS generation and ROS-associated impairment of salivary gland cells<br>• NOX1 mRNA and protein are overexpressed in colon cancer and are strongly correlated with activating mutations in K-Ras<br>• mediates oncogenic Ras-induced upregulation of VEGF and angiogenesis by activating Sp1 through Ras-ERK-dependent phosphorylation of Sp1<br>• controls the persistence of directed cell migration by a Rho-dependent switch of alpha2/alpha3 integrins<br>• mediating role of NOX1-generated reactive oxygen species in cell invasion processes, most notably metalloprotease production and cell motile activity<br>• involved in TP53 deacetylation and its activation may activate SIRT1 and inhibit TP53<br>• controls the balance between goblet and absorptive cell types in the colon by coordinately modulating PI3K/AKT/WNT/CTNNB1 and NOTCH1 signaling	++	++	+	++	++

			• plays a role in host defense, cell growth and differentiation, cell migration, and malignant transformation<br>• NADPH oxidase homologue which requires the regulatory subunits NOXO1 and NOXA1, as well as the flavocytochrome component CYBA for maximal activity<br>• might control autocrine cell growth of liver tumor cells through regulation of the EGFR pathway<br>• NOX1 and CYBB play differential roles in different platelet activation pathways and in thrombosis					
NOX3 (50508) 6q25.1-26	NADPH oxidase 3	NADPH oxidase which constitutively produces superoxide upon formation of a complex with CYBA/p22phox. Plays a role in the biogenesis of otoconia/otolith, which are crystalline structures of the inner ear involved in the perception of gravity.	• NG	++	++	++	++	++
NOX4 (50507) 11q14.3	NADPH oxidase 4	Constitutive NADPH oxidase which generates superoxide intracellularly upon formation of a complex with CYBA/p22phox. Regulates signaling cascades probably through phosphatases inhibition. May function as an oxygen sensor regulating the KCNK3/TASK-1 potassium channel and HIF1A activity. May regulate insulin signaling cascade. May play a role in apoptosis, bone resorption and lipolysaccharide-mediated activation of NFKB. May produce superoxide in the nucleus and play a role in regulating gene expression upon cell stimulation. Isoform 3 is not functional. Isoform 5 and isoform 6 display reduced activity.<br>Isoform 4<br>Involved in redox signaling in vascular cells. Constitutively and NADPH-dependently generates reactive oxygen species (ROS).	• generating reactive oxygen species (ROS) that function in host defense and cellular signaling<br>• critical catalytic component for superoxide production in quiescent vascular smooth muscle cells<br>• may be involved in increased superoxide generation in vascular smooth muscle cells under proinflammatory conditions<br>• with CYBB mediate proliferative response in endothelial cells<br>• major Nox isoform in endothelial cells, forming an active complex with CYBA<br>• contributes to angiotensin II and transforming growth factor-beta redox signaling<br>• central mediator that controls oxidative stress that may lead to mitochondrial dysfunction and cell injury in diseases such as diabetes<br>• increasing oxidative damage leading to loss of replicative potential in HUVECs	++	++	++	++	+

		Modulates the nuclear activation of ERK1/2 and the ELK1 transcription factor, and is capable of inducing nuclear DNA damage. Displays an increased activity relative to isoform 1.	• in cardiac myocytes is potentially a major source of mitochondrial oxidative stress, thereby mediating mitochondrial and cardiac dysfunction during pressure overload<br>• with DUOX2, are required for platelet-derived growth factor (PDGF) induced RB1 phosphorylation in normal fibroblasts<br>• NOX4 and DUOX2 regulate cell cycle entry as part of a p53-dependent checkpoint for proliferation<br>• reactive oxygen species generated by NADPH oxidase 2 and 4 (CYBB and NOX4)are required for chondrogenic differentiation<br>• unique inducible regulator of myocardial angiogenesis, a key determinant of cardiac adaptation to overload stress<br>• unique stress-inducible regulator of myocardial angiogenesis that facilitates adaptation to cardiac overload stress<br>• might be involved in the pathophysiology of lung artery hypertrophy in idiopathic pulmonary fibrosis<br>• acts as an intermediary in the signaling of TGFB1 to facilitate collagen synthesis<br>• is involved in the local stimulatory effects of TGFB1 on collagen accumulation<br>• central role of NOX4 as a mediator of renal cell injury in diabetic kidney disease<br>• expression of NLRP5 and NOX4 proteins are closely related to the follicular development and ovulation with particular regard for ovarian aging<br>• integrin engagement during cell attachment activates POLDIP2/NOX4 to oxidize actin, which modulates focal adhesion (FA) assembly					
NOX5 (79400)	NADPH oxidase 5	Calcium-dependent NADPH oxidase that generates superoxide. Also functions as a	• play an important role in PDGF-induced JAK/STAT activation and human aortic	++	++	++	++	+

15q23		calcium-dependent proton channel and may regulate redox-dependent processes in lymphocytes and spermatozoa. May play a role in cell growth and apoptosis. Isoform v2 and isoform v5 are involved in endothelial generation of reactive oxygen species (ROS), proliferation and angiogenesis and contribute to endothelial response to thrombin.	smooth muscle cell (HASMC) proliferation<br>• being functional, promoting endothelial ROS production, proliferation, and the formation of capillary-like structures and contributing to the endothelial response to thrombin<br>• is the main source of superoxide and is implicated in human spermatozoa motility<br>• role for NOX5-dependent ROS generation in human spermatozoa motility<br>• is the predominant NOX family NADPH oxidase in human spermatozoa					
DUOX1<br>(53905)<br>15q21.1	dual oxidase 1	Generates hydrogen peroxide which is required for the activity of thyroid peroxidase/TPO and lactoperoxidase/LPO. Plays a role in thyroid hormones synthesis and lactoperoxidase-mediated antimicrobial defense at the surface of mucosa. May have its own peroxidase activity through its N-terminal peroxidase-like domain.2 Publications<br>Catalytic Activity<br>H+ + NADH + O2 = H_2O_2 + NAD+	• basic thyroid H202 generating system with DUOX2<br>• involved in mucin induction<br>• involved in innate airway host defense through enhanced production of H2O2 (Boots 2009)<br>• both DUOX1 and DUOX2 play a critical role in the production of H2O2 in the thyroid gland, which is the limiting factor in thyroglobulin iodination and thyroxine synthesis<br>• activation results in the activation of ERK1/2 and NF-KappaB pathways, ADAM17 activation, EGFR ligand shedding leading to amplified epithelial EGFR activation and IL-8 production<br>• either has a unique function or must interact with other protein factors to express its catalytic activity<br>• also involved in thyroid hormonogenesis<br>• despite the high sequence similarity shared between DUOX1 and DUOX2, the two isoforms present distinct regulations, tissue expression and catalytic functions<br>• plays an important role in innate airway epithelial responses to infection or injury<br>• importance of DUOX1 in epithelial redox signaling through reversible S-	+	++	+	++	++

			• glutathionylation of a range of proteins, including proteins involved in cytoskeletal regulation and MAPK signaling pathways involved in cell migration<br>• DUOX1-dependent H_2O_2 production, which induces DNA double-strand breaks, can cause genomic instability and promote the generation of neoplastic cells through its mutagenic effect<br>• changes of intracellular calcium concentration are transformed into redox signals through the calcium-dependent activation of DUOX1<br>• DUOX1 and CYBB play Distinct roles in Redox regulation of epidermal growth factor receptor signaling<br>• both DUOX enzymes (DUOX1, DUOX2) and their respective maturation factors DUOXA1 and DUOXA2 form a stable complex at the cell surface, which is fundamental for their enzymatic activity<br>• is an H_2O_2-generating enzyme related to a wide range of biological features, such as hormone synthesis, host defense, cellular proliferation, and fertilization<br>• thyroid hormone synthesis requires H_2O_2, produced by two NADPH oxidases, DUOX1 and DUOX2					
DUOX2 (50506) 15q21.1	dual oxidase 2	Generates hydrogen peroxide which is required for the activity of thyroid peroxidase/TPO and lactoperoxidase/LPO. Plays a role in thyroid hormones synthesis and lactoperoxidase-mediated antimicrobial defense at the surface of mucosa. May have its own peroxidase activity through its N-terminal peroxidase-like domain.<br>Catalytic Activity<br>$H+ + NADH + O_2 = H_2O_2 + NAD+$	• catalyzing the synthesis of thyroid hormone<br>• essential for iodination of thyroglobulin by thyroid peroxidase and constitutes the rate-limiting step of thyroid hormone synthesis<br>• dual oxydase constitute the catalytic core of the H_2O_2 generator<br>• exhibiting potent heme peroxidase activity in human respiratory tract epithelium<br>• NADPH oxidases producing H_2O_2 necessary for thyroid hormone biosynthesis<br>• with NOX4, are required for platelet-derived	+++	++	+	++	++

			growth factor (PDGF) induced RB1 phosphorylation in normal fibroblasts<br>• NOX4 and DUOX2 regulate cell cycle entry as part of a p53-dependent checkpoint for proliferation<br>• both DUOX1 and DUOX2 play a critical role in the production of H_2O_2 in the thyroid gland, which is the limiting factor in thyroglobulin iodination and thyroxine synthesis<br>• despite the high sequence similarity shared between DUOX1 and DUOX2, the two isoforms present distinct regulations, tissue expression and catalytic functions<br>• both DUOX2 and DUOXA2 expression are involved specifically in inflammation and are regulated on a crypt-by-crypt basis in ulcerative colitis tissue<br>• both stability and function of the maturation factor, DUOXA2, are dependent on the oxidative folding of DUOX2, indicating that DUOX2 displays a chaperone-like function with respect to its partner<br>• both DUOX enzymes (DUOX1, DUOX2) and their respective maturation factors DUOXA1 and DUOXA2 form a stable complex at the cell surface, which is fundamental for their enzymatic activity<br>• thyroid hormone synthesis requires H2O2, produced by two NADPH oxidases, DUOX1 and DUOX2<br>• H_2O_2 produced by the DUOX2/DUOXA2 cell surface enzymatic complex could provoke potential mutagenic DNA damage in an inducible cellular model					
MPO<br>(4353)<br>17q22	myeloperoxidase	Part of the host defense system of polymorphonuclear leukocytes. It is responsible for microbicidal activity against a wide range of organisms. In the stimulated	• involved in oxygen dependent mechanism of phagocytosis and in myeloid differentiation<br>• contributes critically to O_2-dependent neutrophil antimicrobial activity	++	++	+	++	++

	PMN, MPO catalyzes the production of hypohalous acids, primarily hypochlorous acid in physiologic situations, and other toxic intermediates that greatly enhance PMN microbicidal activity.<br>Mediates the proteolytic cleavage of alpha-1-microglobulin to form t-alpha-1-microglobulin, which potently inhibits oxidation of low-density lipoprotein particles and limits vascular damage.<br>Catalytic Activity<br>chloride + H+ + H_2O_2 = H_2O + hypochlorous acid	• implicated in atherosclerosis and cholesterol homeostasis<br>• crucial prerequisite for structural remodeling of the myocardium, leading to an increased vulnerability to atrial fibrillation<br>• acts as a major downstream mediator of atrial fibrosis and atrial arrhythmogeneity<br>• intimately involved in the pathophysiology of atrial fibrillation<br>• is required for neutrophil extracellular traps (NETs) formation<br>• is involved in a multitude of inflammatory processes involving oxidative modification of soluble components and cellular surfaces<br>• plays a key role in promoting atherosclerosis via oxidative stress by modification of both high- and low-density lipoprotein and production of other bioactive molecules<br>• MPO is unique in its capacity to oxidize chloride at physiologic pH to produce hypochlorous acid (HOCl), a potent microbicide that contributes to neutrophil-mediated host defense against infection<br>• MPO, via its catalytic activity, inhibits the generation of adaptive immunity by suppressing dendritic cells (DCs) activation, Ag uptake/processing, and migration to lymph nodes (LNs) to limit pathological tissue inflammation<br>• abundant hemoprotein expressed by neutrophil granulocytes that is recognized to play an important role in the development of vascular diseases<br>• uses hydrogen peroxide generated by the oxidative burst of neutrophils to produce an array of antimicrobial oxidants<br>• has both potent microbicidal and, upon binding to the vessel wall, pro-inflammatory

			properties<br>• role for myeloperoxidase-dependent lipid peroxidation<br>• MPO -dependent lipid peroxidation promotes the oxidative modification of cytosolic proteins in phagocytic neutrophils<br>• MPO may contribute importantly to formation and rupture of cerebral aneurysms<br>• important and previously unsuspected role for MPO and EPX as drivers of angiogenesis<br>• chaperoned by calreticulin (CALR) in the endoplasmic reticulum<br>• MPO and PRTN3 in neutrophils of Anti-neutrophil cytoplasmic autoantibody (ANCA)-associated vasculitis (AAV) patients with active disease have a distinct pattern of histone modifications, which implicates epigenetic mechanisms in regulating expression of autoantigen genes<br>• peroxidase enzymes, like MPO and EPX, may play a fundamental role in inhibiting TNFSF11-induced osteoclast differentiation at inflammatory sites of bone fracture and injury<br>• the difference in MPO between women with abnormal and normal menstrual cycles and the upregulation of MPO before ovulation suggest that neutrophils and MPO are closely related to ovulation<br>• is the major peroxidase enzyme in neutrophil granules and is implicated in contributing to inflammatory lung damage in cystic fibrosis<br>• EPX and MPO are key contributors to cancer progression by augmenting pro-tumorigenic collagen production and angiogenesis<br>• is synthesized by neutrophil and monocyte precursor cells and contributes to host defense by mediating microbial killing					
XDH	xanthine	Key enzyme in purine degradation. Catalyzes	• xanthine oxidase (dehydrogenase), catalyzing	++	++	++	++	++

(7498)<br>2p23.1	dehydrogenase	the oxidation of hypoxanthine to xanthine. Catalyzes the oxidation of xanthine to uric acid. Contributes to the generation of reactive oxygen species. Has also low oxidase activity towards aldehydes (in vitro).<br>Catalytic Activity<br>H_2O + NAD+ + xanthine = H+ + NADH + urate	the oxidation of hypoxanthine to purine and purine to uric acid<br>• plays a physiological role in milk (e.g. in secretion) equal in importance to its catalytic function as an enzyme<br>• synergistic action with ALOXE3, group of oxidized fatty acids which can function as PPAR{gamma} activators<br>• required for functional differentiation of mammary epithelial cells<br>• XDH activity and/or its expression level to contribute to macrophage foam cell formation<br>• activity creates both oxidant and anti-oxidant products that are implicated in the development of hypertension, smoking vascular injury, dyslipidemia and diabetes, which are the main risk factors of atherosclerosis<br>• is abundant in wounds and participates in normal wound healing through effects on ROS production<br>• catalyzes the formation of uric acid (UA) from hypoxanthine and xanthine, which in turn are products of purine metabolism starting from ribose-5-phosphate<br>• XDH activity may be potentially involved in adiposity and subclinical inflammation in humans					
LOX<br>(4015)<br>5q23.1	lysyl oxidase	Responsible for the post-translational oxidative deamination of peptidyl lysine residues in precursors to fibrous collagen and elastin. Regulator of Ras expression. May play a role in tumor suppression. Plays a role in the aortic wall architecture.<br>Catalytic Activity<br>H2O + L-lysyl-[protein] + O_2 = (S)-2-amino-6-oxohexanoyl-[protein] + H_2O_2 + NH4+	• lysyl oxidase, copper dependent, processed by BMP1 to the mature enzyme necessary for the formation of covalent cross-links in collagen and elastic fibers<br>• playing a potential tumor suppressor role but also involved in tumor progression<br>• essential for hypoxia-induced metastasis and is a good therapeutic target for preventing and treating metastases<br>• key enzyme in extracellular matrix maturation	++	++	+++	++	++

			• seems to be involved in the impairment of endothelial function triggered by different pathological conditions<br>• essential for hypoxia-induced metastasis<br>• its enzyme activity is critical for the biosynthesis of mature and functional collagens and elastin<br>• can associate with extracellular and intracellular binding partners to affect its known biological activities as a tumor suppressor and inhibitor of cell proliferation<br>• implicated in chromatin decondensation, decondensation suggested to be promoted by LOX actions on histone H1<br>• essential mediator of STK11-deficiency-elicited lung cancer progression through ECM alteration, especially collagen matrix remodeling<br>• may alter cancer cell growth potential via ECM remodeling<br>• lysosome may regulate the dendrite development of Purkinje cells though degradation of LOX<br>• multifunctional enzyme required for collagen biosynthesis<br>• plays a critical role in vascular remodelling<br>• critical intracellular role for LOX and LOXL2 in transcriptional regulation<br>• enzyme responsible for the formation of collagen cross-links<br>• PLOD1 and lysyl oxidase (LOX), contribute to ECM maturation and stabilization<br>• LOX and PLOD1 are critical players in vascular calcification, and important role of ECM remodeling in this process					
NOS1<br>(4842)<br>12q24.22	nitric oxide synthase 1	Produces nitric oxide (NO) which is a messenger molecule with diverse functions throughout the body. In the brain and	• involved in nitric oxide biosynthesis, electron transport, synaptic transmission, muscle contraction, cell-cell signaling	++	++	+	++	++

peripheral nervous system, NO displays many properties of a neurotransmitter. Probably has nitrosylase activity and mediates cysteine S-nitrosylation of cytoplasmic target proteins such SRR.

Catalytic Activity

H+ + 2 L-arginine + 3 NADPH + 4 O2 = 4 H_2O_2 + 2 L-citrulline + 3 NADP+ + 2 nitric oxide

- has a distinct local role in the physiological regulation of microvascular tone
- negatively regulates adult neurogenesis
- neural stem cells (NSCs)-derived NOS1 stimulates neurogenesis via activating telomerase
- potential anti-inflammatory role of endothelial NOS1 that can attenuate unopposed, proinflammatory cytokine actions
- NOS1 in the collecting duct (CD) is critical in the regulation of fluid-electrolyte balance
- its independent and local regulation of NO levels is crucial for normal cilia function
- plays a critical role in regulating cardiomyocyte function
- fundamental role for NOS1-derived NO in regulating TLR4-mediated inflammatory gene transcription, as well as the intensity and duration of the resulting host immune response
- neuronal NOS1 and endothelial NOS3 are constitutive calcium-dependent forms of the enzyme that regulate neural and vascular function respectively
- NOS1 may be significant in the pathophysiology of human ischemic heart disease with a preservative role in maintaining myocardial homeostasis
- NOS1 plays a critical role in neuronal differentiation of hippocampal neural progenitor cells (NPCs)
- plays an important role in neurite outgrowth and may thus influence brain development, specifically white matter (WM) microstructure
- mediates insulin- and oxidative stress-induced glucose uptake in skeletal muscle myotubes
- is a key arginine metabolising enzyme in the brain, and NOS1-derived nitric oxide (NO)

			plays an important role in regulating glutamatergic neurotransmission					
NOS2 (4843) 17q11.2	nitric oxide synthase 2	Produces nitric oxide (NO) which is a messenger molecule with diverse functions throughout the body.<br>In macrophages, NO mediates tumoricidal and bactericidal actions. Also has nitrosylase activity and mediates cysteine S-nitrosylation of cytoplasmic target proteins such PTGS2/COX2.<br>As component of the iNOS-S100A8/9 transnitrosylase complex involved in the selective inflammatory stimulus-dependent S-nitrosylation of GAPDH on 'Cys-247' implicated in regulation of the GAIT complex activity and probably multiple targets including ANXA5, EZR, MSN and VIM.<br>Involved in inflammation, enhances the synthesis of pro-inflammatory mediators such as IL6 and IL8.<br>Catalytic Activity<br>H+ + 2 L-arginine + 3 NADPH + 4 O_2 = 4 H_2O + 2 L-citrulline + 3 NADP+ + 2 nitric oxide<br>This reaction proceeds in the forward direction.	• producing nitric oxide synthesis from arginine and molecular oxygen<br>• playing a role in neurogenesis<br>• role for IDO1 in the regulation of blood pressure, although the contribution of IDO1 to sepsis-induced hypotension is smaller that that of NOS2 (Hofmann 2010)<br>• role of NOS2 as a critical host factor regulating apoptosis during respiratory syncytial virus (RSV) infection<br>• immune-activated glial cells produced NOS2<br>• its expression is restricted to neurons in the healthy brain but is triggered in microglia upon inflammation<br>• is an important mediator in high-cholesterol diet (HCD)-induced liver fibrosis<br>• NOS2 is calcium-independent and is inducible<br>• is a major signaling molecule involved in innate immunity<br>• is a novel negative regulator of hematopoietic cell migration and prevents egress of hematopoietic stem/progenitor cells (HSPCs) into peripheral blood (PB) during mobilization<br>• crucial role of endogenous NOS2 in promoting optimal IL2 production, proliferation and glycolysis of gamma-delta T cells that may contribute to their regulation at steady state<br>• is necessary for the microbicidal activity of macrophages<br>• regulates a number of cellular processes in these cell types without exerting toxicity	++	++	++	++	++
NOS3 (4846) 7q36.1	nitric oxide synthase 3	Produces nitric oxide (NO) which is implicated in vascular smooth muscle relaxation through a cGMP-mediated signal transduction pathway.<br>NO mediates vascular endothelial growth	• catalyzing the synthesis of NO from l-arginine<br>• plays an important role in adiponectin synthesis in adipocytes by increasing mitochondrial biogenesis and enhancing mitochondrial function	++	++	++	++	++

		factor (VEGF)-induced angiogenesis in coronary vessels and promotes blood clotting through the activation of platelets1 Publication<br>Isoform eNOS13C<br>Lacks eNOS activity, dominant-negative form that may down-regulate eNOS activity by forming heterodimers with isoform 1.<br>Catalytic Activity<br>H+ + 2 L-arginine + 3 NADPH + 4 O_2 = 4 H_2O + 2 L-citrulline + 3 NADP+ + 2 nitric oxide<br>This reaction proceeds in the forward direction.	• novel and important role of NOS3 in chondrocyte proliferation and endochondral bone growth and loss of NOS3 results in premature cell cycle exit and prehypertrophic chondrocyte differentiation during cartilage development<br>• NOS3 and ADH5 play an important role in endothelial cell–mediated postnatal angiogenesis and vascular tone<br>• is a pressure-dependent regulator of intraocular pressure<br>• role for NOS3 in bone growth and metabolism and therefore its contribution may at least in part occur during early skeletal development<br>• both NOS3 in the mother and the conceptus contribute to uteroplacental vascular changes and increased uterine arterial blood flow in normal pregnancy<br>• enzyme responsible for synthesis of nitric-oxide (NO) in the vasculature					
IDO1<br>(3620)<br>8p11.21	indoleamine 2,3-dioxygenase 1	Catalyzes the first and rate limiting step of the catabolism of the essential amino acid tryptophan along the kynurenine pathway.<br>Involved in the peripheral immune tolerance, contributing to maintain homeostasis by preventing autoimmunity or immunopathology that would result from uncontrolled and overreacting immune responses.<br>Tryptophan shortage inhibits T lymphocytes division and accumulation of tryptophan catabolites induces T-cell apoptosis and differentiation of regulatory T-cells.<br>Acts as a suppressor of anti-tumor immunity.<br>Limits the growth of intracellular pathogens by depriving tryptophan.<br>Protects the fetus from maternal immune rejection. Catalytic Activity	• catalyzing the degradation of the essential amino acid L-tryptophan to N-formylkynurenine<br>• heme-containing enzyme, which catalyzes the initial and rate-determining step of L-tryptophan (L-Trp) metabolism via the kynurenine pathway<br>• plays an important role in immune escape through suppressing T-cell function<br>• catalyzing the cleavage of the pyrrol ring of tryptophan and incorporates both atoms of a molecule of oxygen<br>• suppresses T-cell responses and promotes immune tolerance in mammalian pregnancy, tumour resistance, chronic infection, autoimmunity and allergic inflammation<br>• acting as a bridge between dendritic cells and CD4+ regulatory T cells	++	++	+	++	++

		D-tryptophan + O_2 = N-formyl-D-kynurenine	• metabolizing tryptophan to N-formyl-kynurenine under certain pathophysiological conditions<br>• might be involved in chronic inflammatory diseases, such as atherosclerosis, morbid obesity and chronic heart disease<br>• role for IDO1 in the regulation of blood pressure, although the contribution of IDO1 to sepsis-induced hypotension is smaller that that of NOS2<br>• contributes to arterial vessel relaxation and the control of blood pressure<br>• having role in retinal pigment epithelial cell-mediated immune modulation					
TDO2 (6999) 4q31-q32	tryptophan 2,3-dioxygenase	Heme-dependent dioxygenase that catalyzes the oxidative cleavage of the L-tryptophan (L-Trp) pyrrole ring and converts L-tryptophan to N-formyl-L-kynurenine. Catalyzes the oxidative cleavage of the indole moiety.<br>Catalytic Activity<br>L-tryptophan + O_2 = N-formyl-L-kynurenine1	• catalyzing the oxidation of L-tryptophan to formylkynurenine and controling the physiological flux of tryptophan into both the serotonergic and kynureninic pathways	++	++	+	+++	++
AOX1 (316) 2q33	aldehyde oxidase 1	Oxidase with broad substrate specificity, oxidizing aromatic azaheterocycles, such as N1-methylnicotinamide, N-methylphthalazinium and phthalazine, as well as aldehydes, such as benzaldehyde, retinal, pyridoxal, and vanillin. Plays a key role in the metabolism of xenobiotics and drugs containing aromatic azaheterocyclic substituents. Participates in the bioactivation of prodrugs such as famciclovir, catalyzing the oxidation step from 6-deoxypenciclovir to penciclovir, which is a potent antiviral agent. Is probably involved in the regulation of reactive oxygen species homeostasis. May be a prominent source of superoxide generation via the one-electron reduction of molecular oxygen. May also catalyze nitric oxide (NO)	• can catalyze the formation of superoxide	+++	++	+++	+++	+

		production via the reduction of nitrite to NO with NADH or aldehyde as electron donor. May play a role in adipogenesis.						
ALOX12 (239) 17p13.1	arachidonate 12-lipoxygenase, 12S type	Catalyzes the regio and stereo-specific incorporation of molecular oxygen into free and esterified polyunsaturated fatty acids generating lipid hydroperoxides that can be further reduced to the corresponding hydroxy species.<br>Mainly converts arachidonate ((5Z,8Z,11Z,14Z)-eicosatetraenoate) to the specific bioactive lipid (12S)-hydroperoxyeicosatetraenoate/(12S)-HPETE.<br>Through the production of bioactive lipids like (12S)-HPETE it regulates different biological processes including platelet activation. It can also catalyze the epoxidation of double bonds of polyunsaturated fatty acids such as (14S)-hydroperoxy-docosahexaenoate/(14S)-HPDHA resulting in the formation of (13S,14S)-epoxy-DHA. Furthermore, it may participate in the sequential oxidations of DHA ((4Z,7Z,10Z,13Z,16Z,19Z)-docosahexaenoate) to generate specialized pro-resolving mediators (SPMs) like resolvin D5 ((7S,17S)-diHPDHA) and (7S,14S)-diHPDHA, that actively down-regulate the immune response and have anti-aggregation properties with platelets (PubMed:32404334).<br>An additional function involves a multistep process by which it transforms leukotriene A4/LTA4 into the bioactive lipids lipoxin A4/LXA4 and lipoxin B4/LXB4, both are vasoactive and LXA4 may regulate neutrophil function via occupancy of specific recognition sites. Can also peroxidize linoleate ((9Z,12Z)-octadecadienoate) to (13S)-hydroperoxyoctadecadienoate/ (13S-HPODE).	• catalyzing the first step of leukotrienes biosynthesis from arachidonate<br>• lipid peroxidating enzyme implicated in the pathogenesis of inflammatory disorders as well as membrane remodeling<br>• carcinogenesis and atheroma formation<br>• cardiac ALOX12/ALOX15 is involved in the development of heart failure<br>• ALOX12/ALOX15 mediates early stages of adipose tissue inflammation and whole body insulin resistance induced by high fat feeding<br>• ALOX12/ALOX15 is required for the maintenance of long-term hematopoietic stem cells quiescence and number	++	++	+	++	+++

		Due to its role in regulating both the expression of the vascular endothelial growth factor (VEGF, an angiogenic factor involved in the survival and metastasis of solid tumors) and the expression of integrin beta-1 (known to affect tumor cell migration and proliferation), it can be regarded as protumorigenic. Important for cell survival, as it may play a role not only in proliferation but also in the prevention of apoptosis in vascular smooth muscle cells.						
MAOA<br>(4128)<br>Xp11.3	monoamine oxidase A	Catalyzes the oxidative deamination of primary and some secondary amine such as neurotransmitters, with concomitant reduction of oxygen to hydrogen peroxide and has important functions in the metabolism of neuroactive and vasoactive amines in the central nervous system and peripheral tissues.<br>Preferentially oxidizes serotonin.<br>Also catalyzes the oxidative deamination of kynuramine to 3-(2-aminophenyl)-3-oxopropanal that can spontaneously condense to 4-hydroxyquinoline<br>Catalytic Activity<br>a secondary aliphatic amine + H_2O + O_2 = a primary amine + an aldehyde + H_2O_2	• degrading amine neurotransmitters (such as dopamine, norepinephrin and serotonin)<br>• having important functions in the metabolism of neuroactive and vasoactive amines in the central nervous system and peripheral tissues<br>• MAOA, SLC6A2, SLC6A3 may play important roles in regulating maternal monoamine neurotransmitters transferred across the placenta to the fetus<br>• novel roles for MAOA, MAOB and serotonin in the regulation of intermediate progenitor cells proliferation in the developing brain<br>• MAOA and MAOB are a crucial pair of isoenzymes, which oxidatively deaminate monoamine neurotransmitters and dietary amines with a production of hydrogen peroxide<br>• MAOA, and MAOB are dimeric in their membrane-bound forms<br>• MAOA and MAOB are mitochondrial-bound proteins, catalyzing the oxidative deamination of monoamine neurotransmitters as well as xenobiotic amines<br>• primarily deaminates serotonin, norepinephrine, and dopamine and, therefore, is implicated in several psychiatric diseases	+++	++	+++	++	+
MAOB	monoamine oxidase	Catalyzes the oxidative deamination of	• degrading amine neurotransmitters	++	++	+	++	++

(4129)<br>Xp11.23	B	primary and some secondary amines such as neurotransmitters, and exogenous amines including the tertiary amine, neurotoxin 1-methyl-4-phenyl-1,2,3,6-tetrahydropyridine (MPTP), with concomitant reduction of oxygen to hydrogen peroxide and participates in the metabolism of neuroactive and vasoactive amines in the central nervous system and peripheral tissues. Preferentially degrades benzylamine and phenylethylamine.<br>Catalytic Activity<br>a secondary aliphatic amine + H_2O + O_2 = a primary amine + an aldehyde + H_2O_2	• catalyzing the oxidative deamination of biogenic and xenobiotic amines and playing an important role in the metabolism of neuroactive and vasoactive amines in the central nervous system and peripheral tissues<br>• novel roles for MAOA, MAOB and serotonin in the regulation of intermediate progenitor cells proliferation in the developing brain<br>• PARK2 suppresses the transcription of MAOA, MAOB to control oxidative stress induced by dopamine oxidation<br>• MAOA and MAOB are a crucial pair of isoenzymes, which oxidatively deaminate monoamine neurotransmitters and dietary amines with a production of hydrogen peroxide<br>• MAOA, and MAOB are dimeric in their membrane-bound forms<br>• MAOA and MAOB are mitochondrial-bound proteins, catalyzing the oxidative deamination of monoamine neurotransmitters as well as xenobiotic amines<br>• plays an important role in the metabolism of neuroactive and vasoactive amines in the central nervous system and peripheral tissues

[1] Human Gene Nomenclature Committee (https://www.genenames.org); [2] Entrez Gene (https://www.ncbi.nlm.nih.gov/gene) ; [3] UniProt (https://www.uniprot.org) ; [4] GENATLAS (http://genatlas.medecine.univ-paris5.fr/); [5] BioGPS (http://biogps.org). The number of the "+" symbols means the level of gene expression in the tissues/organs playing a role in the pathogenesis of type 2 diabetes: low (+), medium (++) and high (+++). "-" depicts that the gene expression is too low or not detected (<0.5 Me).
Low expression: >0.5 Me, <1 Me; medium expression: >1 Me, <10 Me; high expression: >10 Me.
