# Supplementary material for: Molecular Genetics of Abnormal Redox Homeostasis in Type 2 Diabetes Mellitus
Source: Int J Mol Sci. 2023 Mar 1;24(5):4738. doi: 10.3390/ijms24054738 (PMC10003739; doi:10.3390/ijms24054738)
Supplement: Supplementary file 1 [file ijms-24-04738-s001.zip › Supplementary Table S2.pdf]

Supplementary Table S2. eQTL-analysis of SNPs associated with T2D

Gene	SNP	Location	SNP type	Alleles	Ancestral	Assessed allele	eQTLGen (blood)		Pancreas	Liver	Adipose - Visceral	Skeletal muscle	Brain	Tibial nerve	Artery/ Aorta	Kidney	sQTLs
							Cis	*Trans*									
GCLC	rs17883901	6:53545239	intron variant	G/A	G	A	-	-	-	-	GCLC(↑)	-	-	--	-	-	
GCLC	rs761142	6:53527310	intron variant	A/C	C	C	GCLC (↓)	-	-	-	-	-	GCLC (↓	-	-	-	-
GCLM	rs7517826	1:93899778	intron variant	C/A	C	A	GCLM (↑)<br>DR1(↑)<br>DNTTIP2 (↓<br>BCAR3(↓)<br>TMED5(↓)	-	GCLM(↓<br>RP11-488P3,1(↓	-	GCLM(↓)<br>RP11-488P3,1(↓)	GCLM(↓)<br>RP11-488P3,1(↓)	GCLC (↓	GCLM(↓)	RP11-488P3,1(↓)<br>GCLM(↓)<br>RP11-488P3,1(↓)<br>GCLM(↓)<br>BCAR3(↓)	-	DNTTIP2(Arter
GCLM	rs41303970	1:93909753	TF binding site	G/A	G	A	BCAR3(↓)	-	GCLM (↓	-	GCLM (↓)	GCLM (↓)	GCLM (↓	GCLM(↓)	GCLM (↓)		-
GSS	rs6088660	20:34955093	5 prime UTR variant	C/T	C	T	GSS(↑)<br>TRPC4AP(↑<br>EDEM2(↑)<br>NCOA6(↑)<br>GGT7(↓)<br>MAP1LC3A (<br>EIF6 (↓)<br>TP53INP2 (↓<br>MYH7B (↓)<br>RP4-614O4,12(↓)	-	-	-	EDEM2(↑)	GSS(↑)<br>GGT7(↑)<br>NCOA6(↑	NCOA6(↑	GSS(↑)<br>TRPC4AP(<br>ACSS2(↑)<br>NCOA6 (↑<br>MYH7B(↓	NCOA6(↑<br>MYH7B(↓<br>TP53INP2(<br>GSS(↓)<br>PIGU(↓)<br>TRPC4AP(	-	TRPC4AP (Pancreas, Live Adipose – Visceral, Skelet muscle, Brain) PROCR Skeleta muscle
GSS	rs13041792	20:34957252	intergenic variant	G/A	G	A	MAP1LC3A(<br>MYH7B(↑)<br>EIF2S2(↑)<br>ITCH(↑)<br>RBM39(↑)<br>TRPC4AP(↓<br>EDEM2(↓)<br>UQCC(↓)<br>CPNE1(↓)<br>GGT7(↓)<br>RP4-614O4,12(↓)<br>PROCR(↓)<br>MMP24-AS1<br>CEP250(↓)	-	EDEM2(↑<br>PROCR(↑<br>MYH7B(↑	PROCR(↑<br>TRPC4AI ↓)<br>MYH7B(↑	MAP1LC3A ↑)<br>MYH7B(↑<br>EDEM2(↓)<br>TRPC4AP(	EDEM2(↓)<br>PROCR(↓)<br>GGT7(↑)<br>NFS1(↑)	MYH7B(↑<br>EDEM2(↓<br>MAP1LC A(↑)<br>MMP24-AS1(↓)<br>ACSS2(↑	-	EDEM2(↓)<br>MYH7B(↑<br>PROCR(↓)<br>MAP1LC3A ↑)	-	UQCC1 (Skeleta muscle)<br>TRPC4AP (Brai Artery)<br>PROCR (Artery nerve)
GGT1	rs4820599	22:24594246	intron variant	A/G	G	G	GGT1(↓)<br>FAM211B(↓<br>BCRP3(↓)	KIR2DL3(↓)	GGT1(↑	-	-	-	-	-	-	-	BCRP3(Brain)

GGT1	rs5751909	22:24617379	intron variant	G/A	G	A	GGT1(↓)<br>FAM211B(↓)<br>BCRP3(↓)<br>SGSM1(↓)	-	GGT1(↑)	-	-	-	-	BCRP3(↓)	-		BCRP3(Brain)
GGT1	rs5760489	22:24594679	intron variant	A/G	G	G	GGT1(↓)<br>FAM211B(↓)<br>BCRP3(↓)	-	GGT1(↑)	-	-	-	-	LRRC75B(↓	-	-	BCRP3(Brain)
GGT1	rs5760492	22:24599235	non coding transcrip t exon variant	G/A	G	A	GGT1(↓)<br>FAM211B(↓)<br>BCRP3(↓)<br>SGSM1(↓)	-	GGT1(↑)	GGT1(↑)	-	-	GGT1(↓)	GGT1(↓)<br>LRRC75B(↓	-	-	BCRP3(Brain)
GGT5	rs2275984	22:24226680	missens e variant	T/C?	C	C	GGT5(↓)<br>GGT1(↓)<br>GSTT1(↓)<br>UPB1(↓)<br>AP000355,2(↓<br>DDT(↓)<br>SUSD2(↓)<br>SPECC1L(↓)<br>ADORA2A(↑	-	POM121L<br>P(↓)	-	POM121L9I<br>↓)	POM121L9I<br>↓)	POM121L<br>P(↓)	POM121L9<br>UPB1	POM121L9I<br>↓)<br>SPECC1L(↑	-	-
GGT5	rs8140505	22:24220908	intron variant	A/G	G	G	GSTT1(↑)<br>SUSD2(↑)<br>DDT(↑)<br>AP000351,10(<br>AP000355,2(↓<br>UPB1(↓)	-	-	-	POM121L9I<br>↓)	AP000351,4(<br>)	-	POM121L9I<br>↓)	-	-	-
GGT5	rs4822473	22:24235823	intron variant	A/G	A	G	GSTT1(↑)<br>SUSD2(↑)<br>AP000351,10(<br>DDT(↑)<br>SPECC1L(↑)<br>AP000350,6(↑<br>ADORA2A(↑<br>UPB1(↓)<br>AP000355,2(↓	-	-	-	POM121L9I<br>↓)	GGT5(↑)	-	POM121L9I<br>↓)	POM121L9I<br>↓)	-	-
GGT6	rs11657054	17:4559728	missens e variant	G/A	G	G	CXCL16(↑)<br>MYBBP1A(↓	-	GGT6(↑)	GGT6(↑)	MYBBP1A(	-	-	MYBBP1A(	MYBBP1A(	-	-
GGT6	rs2100986	17:4562207	regulato ry region variant	C/G/T	T	C	CXCL16(↑)<br>ALOX15(↑)<br>PLD2(↓)<br>MYBBP1A(↓	-	GGT6(↑)	-	MYBBP1A(	-	-	MYBBP1A	MYBBP1A(	-	-
GGT7	rs11546155	20:34863345	synony mous variant	G/A	G	A	TRPC4AP(↑)<br>NCOA6(↑)<br>EDEM2(↑)<br>EIF6(↑)<br>GSS(↑)<br>PROCR(↑)	-	-	-	GGT7(↓)<br>NCOA6(↑)<br>MAP1LC3	PIGU(↑)<br>GSS(↑)	NCOA6(↑	NCOA6(↑)<br>MAP1LC3<br>↓)<br>FDX1P1(↓)	TP53INP2(↓<br>NCOA6<br>MAP1LC3<br>↓)<br>GGT7(↓)<br>PIGU		TRPC4AP (adipose)

							MAP1LC3A(<br>TP53INP2(↓<br>ITCH(↓)<br>RP4-<br>614O4,12(↓)										
GGT7	rs6119534	20:34853454	intron variant	C/G/T	C	T	MAP1LC3A(<br>ITCH(↑)<br>EIF2S2(↑)<br>MYH7B(↑)<br>EDEM2(↓)<br>GGT7(↓)<br>RP4-<br>614O4,12(↓)<br>UQCC(↓)<br>PROCR(↓)<br>RP5-<br>1125A11,1(↓<br>TRPC4AP(↓	-	EDEM2(↑<br>PROCR(↑<br>MYH7B(↑	MAP1LC<br>A(↑)	MAP1LC3A<br>↑)	GGT7(↑)<br>ACSS2 (↑)<br>MAP1LC3A<br>↑)<br>GSS(↑)<br>PROCR(↓)<br>EDEM2(↓)	EDEM2(↓<br>MYH7B(↑<br>MAP1LC<br>A(↑)<br>ACSS2(↑	MAP1LC3A<br>↑)<br>ACSS2(↑)<br>MYH7B(↑<br>FDX1P1(↑<br>EDEM2(↓)<br>RP5-<br>1125A11,7(<br>PROCR(↓)	MAP1LC3A<br>↑)<br>EDEM2(↓)<br>PROCR(↓)<br>TP53INP2(↑<br>NCOA6(↑		TRPC4AP (arter<br>adipose)<br>GSS (skeletal,<br>adipose)<br>MAP1LC3A(Bra<br>n)<br>GGT7 (skeletal
GGCT	rs6462210	7:30499979	intron variant	C/T	T	T	AC005154,8(↓<br>AC005154,6(↓	-	AC005154<br>(↓)	-	-	AC005154,6<br>)	-	AC005154,6<br>)	-	-	AC005154,6<br>(adipose)
GGCT	rs28679	7:30502181	intron variant	A/G	A	A	GGCT(↑)<br>AC005154,6(↑<br>AC005154,8(↑<br>CRHR2(↑)<br>GARS(↑)<br>AC005154,5(↓	-	GGCT(↓<br>AC005154<br>(↓)<br>AC005154<br>(↓)<br>AC005154<br>(↓)	AC005154<br>(↓)	AC005154,<br>(↓)<br>AC005154,7<br>)	GGCT(↓)<br>AC005154,6<br>)	GGCT(↓<br>AC005154<br>(↓)<br>AC005154<br>(↓)<br>CRHR2(↓	GGCT(↓)<br>AC005154,6<br>)<br>AC005154,8<br>)<br>RP4-<br>777O23,1(↓<br>AC005154,7<br>)	GGCT(↓)<br>AC005154,6<br>)<br>AC005154,7<br>)<br>AC005154,8<br>)<br>RP4-<br>777O23,1(↓	-	AC005154,6<br>(skeletal, adipos<br>artery, brain,<br>liver, pancreas<br>nerve)<br>GGCT (artery,<br>brain, pancreas<br>GGCT (nerve)
GSTP1	rs1695	11:67585218	missens e variant	A/G		G	-	-	DOC2GP(<br>)<br>NDUFV1(<br>)	NUDT8(↑<br>CDK2AP2<br>↑)<br>RPS6KB2(<br>)	GSTP1(↓)<br>RPS6KB2(↓<br>NDUFV1(↑	GSTP1(↓)<br>RPS6KB2(↓<br>AIP(↓)<br>DOC2GP (↓<br>NUDT8(↑)	GSTP1(↓<br>DOC2GP<br>(↓)	GSTP1(↓)<br>DOC2GP(↓<br>NDUFV1(↑<br>NUDT8(↑)	GSTP1(↓)<br>RPS6KB2(↓<br>DOC2GP(↑<br>NUDT8(↑)<br>NDUFV1(↑	-	DOC2GP (artery<br>NDUFV1<br>(skeletal,pancrea<br>RPS6KB2<br>(skeletal, artery<br>adipose)<br>NDUFV1 (Brain<br>artery, adipose
GSTP1	rs1138272	11:67586108	missens e variant	C/T		T)	DOC2GP(↑)<br>RPS6KB2(↑)<br>ANKRD13D(<br>C11orf80(↑)<br>LRFN4(↑)<br>CCS(↓)<br>CLCF1(↓)<br>SSH3(↓)<br>POLD4(↓)<br>AIP(↓)	-	DOC2GP(<br>)<br>ANKRD1<br>D(↑)	ANKRD1<br>D(↑)	ANKRD13D<br>↑)<br>DOC2GP(↓<br>NDUFV1(↓	ANKRD13D<br>↑)<br>PC(↑)<br>DOC2GP(↓<br>RPS6KB2(↓	DOC2GP(<br>)	CLCF1(↓)<br>DOC2GP(↓<br>RAD9A(↓)<br>NDUFV1(↑<br>ANKRD13D<br>↑)	GSTP1(↓)<br>RPS6KB2(↓<br>ANKRD13D<br>↑)	-	-

							CORO1B(↓)										
RAC1	rs10951982	7:6382925	intron variant	G/A/T	G	A	DAGLB(↑)<br>FAM220A(↑<br>ZDHHC4(↓)	-	DAGLB(↑	-	DAGLB(↑)	RAC1(↓)<br>DAGLB(↑)	DAGLB(↑	DAGLB (↑<br>RAC1(↑)	DAGLB(↑)	-	DAGLB (nerve<br>artery, brain)<br>DAGLB (adipos<br>pancreas, kidne<br>liver,)<br>RAC1 (artery)
RAC1	rs4724800	7:6370897	intergen ic variant	A/G	A	G	DAGLB(↑)<br>FAM220A(↑<br>CCZ1B(↑)<br>CCZ1(↓)	-	DAGLB(↑	-	DAGLB(↑)	DAGLB(↑)	DAGLB(↑	DAGLB(↑)	DAGLB(↑)	-	DAGLB (adipos<br>nerve, artery)<br>DAGLB (brain<br>kidney,<br>pancreas,<br>skeletal)<br>RAC1 (artery)
RAC1	rs7784465	7:6378644	intron variant	T/C	T	C	RAC1(↑)<br>KDELR2(↑)<br>FAM220A(↑<br>DAGLB(↓)<br>ZDHHC4(↓)	-	-	-	RAC1(↑)	-	RAC1(↑)	RAC1(↑)	-	-	-
RAC1	rs836478	7:6392059	intron variant	C/T	T	**T**	DAGLB(↑)<br>RAC1(↑)<br>FAM220A(↑<br>GRID2IP(↑)<br>AC073343,11(<br>CCZ1B(↓)	-	DAGLB(↑	-	DAGLB(↑)	-	DAGLB(↑	RAC1<br>DAGLB	DAGLB(↑)	-	RAC1 (skeletal<br>DAGLB (artery<br>brain, skeletal)
RAC1	rs9374	7:6402740	3 prime UTR variant	G/A?	G	A	-	-	DAGLB(↑	DAGLB(↑	DAGLB(↑)	RAC1(↓)<br>DAGLB(↑)	RAC1(↑)<br>DAGLB(↑	RAC1(↑)<br>DAGLB(↑)	DAGLB(↑)	-	DAGLB (artery<br>DAGLB (adipos<br>brain, kidney,<br>liver, skeletal,<br>pancreas)
RAC1	rs10238136	7:6383715	intron variant	A/T	A	T	RAC1(↑)<br>KDELR2(↓)<br>DAGLB(↓)	-	-	-	-	-	-	-	-	-	-
RAC2	rs2239774	22:37241613	synony mous variant	G/C/T	G	C	RAC2(↑)<br>RP1-<br>151B14,9(↑)<br>SSTR3(↑)	-	-	-	-	-	-	-	-	-	-
CYBA	rs9932581	16:88651945	3 prime UTR variant	C/T	C	T	MVD(↑)<br>RNF166(↑)<br>SNAI3-AS1(↑<br>GALNS(↓)<br>TRAPPC2L(↓	-		-	-	-	MVD(↑)	-	-	-	-
CYBA	rs4673	16:88646828	missens e variant	A/G/T	A	A	SNAI3-AS1(↑<br>RNF166(↓)<br>SNAI3(↓)<br>MVD(↓)	-	-	-	-	-	-	CYBA (G) (<br>SNAI3-<br>AS1(G)(↓)	CYBA (G)(↓<br>SNAI3-<br>AS1(G)(↓)	-	-

CYBB	rs5963327	X:37813966	intron variant	G/T	G	T	-	-	-	-	-	XK(↑)	DYNLT3(	CYBB(↑)	CYBB(↑)	-	-
CYBB	rs6610650	X:37777261	intron variant	G/A	A	A	-	-	-	-	-	XK (↑)<br>DYNLT3(↓	DYNLT3(	CYBB(↑)	-	-	
NCF2	rs3754515	1:183593647	intron variant	C/A	C	A	NCF2(↑)<br>ARPC5(↑)<br>SMG7(↑)<br>RGL1(↑)<br>RP1-127C7,6(	-	-	ARPC5(↑	ARPC5(↑)	ARPC5(↑)<br>NCF2(↓)	ARPC5(↑	ARPC5(↑)	ARPC5(↑)<br>NCF2(↓)	-	ARPC5 (artery
NCF2	rs789180	1:183556485	intron variant	A/T?	A	T	NCF2(↑)<br>ARPC5(↓)<br>RGL1(↓)	-	-	-	-	-	-	-	-	-	
NCF2	rs10911363	1:183580622	intron variant	G/T	G	T	NCF2(↑)<br>SMG7(↓)<br>ARPC5(↓)<br>RGL1(↓)<br>SMG7-AS1(↓	-	-	-	SMG7(↓)	NMNAT2(	SMG7(↓	ARPC5(↓)<br>SMG7(↓)	NCF2(↑)<br>NMNAT2(<br>SMG7(↓)	-	NMNAT2 (Brai
NCF2	rs17849502	1:183563445	missense variant	G/A/C/T	G	T	NCF2(↑)<br>SMG7(↓)<br>ARPC5(↓)<br>RGL1(↓)	-	-	-	-	-	-	-	-	-	-
NCF2	rs796860	1:183555720	3 prime UTR variant	C/A	A	C	SMG7(↑)<br>NCF2(↓)<br>ARPC5(↓)<br>RGL1(↓)	-	-	-	SMG7-AS1(	-	ARPC5(↑	SMG7(↓)<br>SMG7-AS1(	NMNAT2(	-	-
NCF4	rs2075938	22:36870280	non coding transcript exon variant	A/G	G	A	TST(↑)<br>PVALB(↑)<br>CSF2RB(↑)<br>MPST(↑)<br>CSF2RBP1(↑<br>IFT27(↑)<br>CTA-833B7,2(<br>snoU13(↑)	-	-	-	-	-	-	-	-	-	
NCF4	rs4821544	22:36862461	intron variant	T/A/C	C	C	PVALB(↓)<br>TST(↓)<br>NCF4(↓)	HNRNPA1P2<br>↑)<br>RTKN2(↑)	-	-	NCF4(↓)	-	-	NCF4(↓)	NCF4(↓)	-	-
NCF4	rs2075939	22:36875840	missense variant	T/C	C	T	PVALB(↑)<br>TST(↑)<br>CSF2RB(↑)<br>NCF4(↓)<br>MPST(↑)<br>IFT27(↑)<br>CSF2RBP1(↑<br>snoU13(↑)	-	-	-	-	-	-	-	-	-	-
NCF4	rs5995355	22:36855419	intron variant	A/G/T	G	G	PVALB(↓)<br>TST(↓)	-	-	-	-	-	-	-	-	-	-
NCF4	rs760519	22:36867664	intron	C/G/T	T	C	PVALB(↑)	-	-	-	-	-	-	-	-	-	-

			variant				TST(↑)<br>CSF2RB(↑)<br>IFT27(↑)<br>MPST(↑)<br>CSF2RBP1(↑<br>snoU13(↑)<br>CTA-833B7,2(										
NOXA1	rs28649497	9:137434689	3 prime UTR variant	C/T	T	T	NSMF(↑)<br>NOXA1(↑)<br>EXD3(↑)	-	NOXA1(↑<br>ENTPD8(	NOXA1(↑	NOXA1(↑	NOXA1(↑	NOXA1(↑	NOXA1(↑<br>ENTPD8(↓<br>EXD3(↓)	NOXA1(↑<br>ENTPD8(↓	-	NOXA1(brain, artery,pancreas skeletal, liver)<br>NOXA1(nerve)
NOXA1	rs6606588	9:137422367	intron variant	G/A?	G	A	NSMF(↑)<br>NOXA1(↑)<br>EXD3(↑)	-	NOXA1(↑<br>ENTPD8(	NOXA1(↑	NOXA1(↑	NOXA1(↑	NOXA1(↑	NOXA1(↑<br>ENTPD8(↓	NOXA1(↑<br>ENTPD8(↓	-	NOXA1 (artery pancreas, skeletal, liver, brain,adopose)<br>NOXA1(nerve)
NOX1	rs5921668	X:100847189	intron variant	G/A/C	A	A	-	-	SYTL4(↑	NOX1(↑	SYTL4(↑)<br>TSPAN6(↓	SYTL4(↑)<br>TSPAN6(↓	NOX1(↑<br>SYTL4(↑	NOX1(↑)<br>TSPAN6(↓	NOX1 (↑)<br>SYTL4(↑)<br>SRPX2(↓)<br>TSPAN6(↓	-	-
NOX1	rs5921682	X:100875448	intergenic variant	A/G?	A	G	-	-	SYTL4 (↑	-	-	-	NOX1(↑	-	SYTL4 (↑)	-	-
NOX4	rs2164521	11:89476817	intron variant	G/A	A	-	-	-	-	-	-	-	-	-	-	-	-
NOX4	rs2202150	11:89410138	intron variant	A/G	A	-	-	-	-	-	-	-	-	-	-	-	-
NOX5	rs438866	15:69023103	non coding transcript exon variant	C/T	C	-	-	-	-	-	-	-	-	-	-	-	-
NOX5	rs35672233	15:68930044	intron variant	C/T	C	-	-	-	-	-	-	-	-	-	-	-	-
BECN1	rs10512488	17:42811886	non coding transcript exon variant	G/A	G	A	NT5C3B(↑)<br>FAM134C(↑<br>NAGLU(↑)<br>TUBG2(↑)<br>NBR2(↑)<br>CTD-3199J23,6(↑)<br>HSD17B1P1(↓<br>BECN1(↓)	-	-	-	-	HSD17B1P1<br>↓)<br>CTD-3014M21,1(<br>HSD17B1(↓	-	NBR2(↑)<br>EZH1(↑)	RP11-400F19,18(↑<br>NBR2(↑)	-	MLX (skeletal l,<br>RAMP2-AS1(nerve,<br>TUBG1 (brain)<br>TUBG2 (nerve)
GLIS3	rs10758593	9:4292083	intron variant	G/A	A	-	-	-	-	-	-	-	-	-	-	-	-

GLIS3	rs10814916	9:4293150	intron variant	A/C	A	**C**	GLIS3(↓)	-	-	-	-	-	-	-	-	-	-
GYS2	rs10841855	12:21599174	intron variant	G/T	G	T	GOLT1B(↓)<br>C12orf39(↓)	-	-	-	-	-	-	PYROXD1(´	PYROXD1(´	-	-
GRK5	rs10886471	10:119389891	intron variant	C/T	C	T	GRK5(↓)	-	-	-	-	-	-	-	-	-	-
PPARG	rs11709077	3:12295008	intron variant	G/A	G	A	TSEN2(↑)<br>SYN2(↓)	-	-	-	-	SYN2(↑)	-	-	-	-	-
IGF2BP2	rs11927381	3:185790803	intron variant	T/C	C	C	LIPH(↑)<br>EHHADH(↓	-	-	-	-	-	-	-	-	-	-
KCNQ1	rs2237896	11:2837210	intron variant	G/A/T	G	A	CDKN1C(↓)<br>NAP1L4(↓)	-	-	-	-	-	-	TSPAN32(↓	-	-	-
DAAM1	rs35209784	14:59285112	intron variant	C/T	C	T	L3HYPDH(↑	-	-	-	-	-	-	DAAM1(↑	-	-	-
HNF1B	rs4430796	17:37738049,	intron variant	A/G	G	-	-	-	-	-	-	-	-	-	-	-	-
CDKAL1	rs4712524	6:20657634	intron variant	A/G	A	G	CDKAL1(↑)	-	-	-	-	-	-	-	-	-	-
CDKAL1	rs6931514	6:20703721	intron variant	A/G	A	G	CDKAL1(↑)	-	-	-	-	-	-	-	-	-	-
TCF7L2	rs7903146	10:112998590	intron variant	C/G/T	T	T	-	-	-	-	-	-	-	-	TCF7L2(↓	-	-
